# Companionship at hospital discharge and its association with subsequent delirium onset in older adults – the TRADE observational study

**DOI:** 10.1186/s12877-026-07194-3

**Published:** 2026-02-21

**Authors:** Simone Brefka, Judith Adamo, Christoph Leinert, Johanna Braisch, Genia Decker, Rainer Muche, Thomas Seufferlein, Jochen Klaus, Lena Schulte-Kemna, Gerhard Eschweiler, Florian Gebhard, Konrad Schuetze, Tobias Geisler, Anke Bahrmann, Hugo A. Katus, Norbert Frey, Natascha-Elisabeth Denninger, Martin Müller, Kathrin Pahmeier, Janine Biermann-Stallwitz, Juergen Wasem, Anna Lena Flagmeier, Petra Benzinger, Juergen Bauer, Michael Denkinger, Dhayana Dallmeier

**Affiliations:** 1 Institute for Geriatric Research, Ulm University Medical Center, Ulm, Germany; 2 Geriatric Center Ulm, Ulm, Germany; 3 Research Unit on Ageing, AGAPLESION Bethesda Hospital Ulm, Ulm, Germany; 4 Institute for Psychogerontology, Friedrich-Alexander University Erlangen-Nuremberg, Nuremberg, Germany; 5 Institute for Epidemiology and Medical Biometry, Ulm University, Ulm, Germany; 6 Department of Internal Medicine I, University Hospital Ulm, Ulm, Germany; 7 Geriatric Center at University Hospital Tuebingen, Tuebingen, Germany; 8 Department of Trauma-, Hand-, and Reconstructive Surgery, University Hospital Ulm, Ulm, Germany; 9 Department of Cardiology, University Hospital Tuebingen, Tuebingen, Germany; 10 Department of Cardiology, Angiology and Pneumology, University Hospital Heidelberg, Heidelberg, Germany; 11 Department for Primary Care and Health Services Research, Nursing Science and Interprofessional Care, Medical Faculty Heidelberg, Heidelberg University, Heidelberg, Germany; 12 International Graduate Academy, Institute for Health and Nursing Science, Medical Faculty, Martin Luther University Halle-Wittenberg, Halle (Saale), Germany; 13 Institute for Health Care Management and Research, University of Duisburg-Essen, Essen, Germany; 14 AOK – Allgemeine Ortskrankenkasse Baden-Wuerttemberg, Statutory Health Insurance Company, Stuttgart, Germany; 15 Geriatric Center, Medical Faculty Heidelberg, Heidelberg University, Heidelberg, Germany; 16 Institute of Health and Generations, Faculty of Social and Health Studies, University of Applied Sciences Kempten, Kempten, Germany; 17 Department of Epidemiology, Boston University School of Public Health, Boston, MA USA

**Keywords:** Older adults, Delirium, Companionship, Discharge, Transfer, Caregiver, Family

## Abstract

**Background:**

Little is known about the role of cross-sectoral companionship (CSC) at hospital discharge or transfer to another facility/department and subsequent delirium incidence in older adults. We aimed to identify predictors of optimal CSC, and to evaluate its association with delirium incidence after discharge/transfer in this population.

**Methods:**

Participants aged ≥ 70 years and their caregivers were recruited in four hospitals in Germany from 08/2019 to 02/2020, and asked standardized questionnaires prior to as well as 3, 7 and 90 days after discharge/transfer. CSC was classified according to its occurrence: *before*,* during and/or after* discharge/transfer, defined as optimal when reported *before and after* or *before*,* during and after*. Incident delirium was identified by confusion assessment method (CAM), family-CAM (FAM-CAM) and nursing delirium screening scale (Nu-DESC) (composite outcome). Logistic regression was performed to identify predictors of optimal CSC, and to evaluate the association between CSC and delirium.

**Results:**

Among 163 participants (median age 80.8 years, 55.8% women) 61 (37.4%) reported optimal CSC, with social contact with daughter and son-in-law and length of hospital stay identified as positive, and former alcohol consumption and transport by ambulance as negative predictors. Among 92 participants (median age 81.6 years, 56.5% women) with complete data on the presence or absence of delirium a 20.7% [95% CI 13.6; 30.0] 7-days delirium incidence proportion was observed. No association between optimal CSC and subsequent delirium onset was detected.

**Conclusion:**

Only one third of patients reported optimal CSC at discharge/transfer, with social contact and length of hospital stay as positive, and former alcohol consumption and the use of an ambulance as negative predictors for an optimal CSC. Although no association between CSC and subsequent delirium could be identified in the present study, the fact that one fifth of discharged patients developed delirium highlights the importance of delirium-preventing measures around the time of discharge.

**Trial registration:**

DRKS (Deutsches Register klinischer Studien) DRKS00017828. Registered on 17th September 2019.

**Supplementary Information:**

The online version contains supplementary material available at 10.1186/s12877-026-07194-3.

## Background

Delirium is a serious medical condition and may occur in every age group, with its frequency increasing with age. Its overall occurrence including prevalence at admission and incidence during hospital stay among older adults is reported to be 29–64% in general and geriatric wards, and 11–51% in surgical wards, while delirium incidence in intensive care units (ICU) amounts to up to 82% [1]. On admission to nursing homes, delirium has been diagnosed in 1 in 7 persons [2]. According to the Confusion Assessment Method (CAM), the diagnostic criteria for delirium include acute onset and/or fluctuating course of the symptoms, inattention, as well as disorganized thinking or altered level of consciousness [3]. Internal triggers such as acute infections, but also external factors that require attention, perception, and orientation, such as unknown surroundings, are known to contribute to the development of delirium. However, the exact pathophysiological mechanism behind the observed increased risk by external factors is not totally understood. An increased level of stress in the presence of reduced cognitive reserve has been proposed in this context [4, 5]. In this regard, limited data are available about delirium associated with a change of location, particularly in the context of hospital discharge or transfer to another institution. The few studies evaluating delirium occurrence with respect to room changes have shown an increased risk for delirium incidence as well as a higher delirium severity related to the number of room changes [6–8].

Fortunately, delirium is considered to be potentially preventable in about one third of cases and reversible [1, 9]. In several clinical studies and delirium prevention programs for inpatients, non-pharmacological preventive and therapeutic interventions for delirium have been evaluated [10–15], with companionship being one important intervention component. Studies on the companionship of older people in the context of discharge are often qualitative, interventional, with support provided by trained individuals and without a focus on the occurrence of delirium [16–18]. The importance of integrating family members or caregivers in the treatment and care of older inpatients is well-recognized [19, 20], as they are particularly concerned about patients’ well-being, have the best knowledge of their relatives, are close to them, and provide a feeling of trust and safety. The current German expert standard for “discharge management in nursing” generally recommends the involvement of relatives/caregivers in the discharge process to provide continuity of care [21]. In addition, companionship by relatives for the purpose of delirium prevention during hospital stay has been shown to be feasible and effective [22, 23]. The exchange with caregivers can comprise substantial delirium-preventive aspects, such as orientation of the older patients, for example by bringing a clock with a date display and placing it in a clearly visible position, or by providing orientation information about the time, place, and situation during conversations. In addition, the transfer of relevant information to follow-up care can also be carried on by caregivers, supporting patient’s management with respect to medication, among others.

All these together build the fundament of the TRAnsport and DElirium in older people (TRADE) project, which intends to evaluate the role of companionship by caregivers, defined as family members or other close relatives who care for or support the patient, at hospital discharge or transfer in the prevention of the subsequent onset of delirium, with the hypothesis that an intervention promoting companionship may reduce the delirium incidence in the settings of change of location at discharge or transfer [24, 25]. This article presents the results of the TRADE observational study, aiming to explore the current state with respect to companionship among older adults discharged from acute care hospitals identifying at the same time potential predictors of optimal cross-sectoral companionship (CSC) in an exploratory manner. Furthermore, the incidence of delirium as well as the association between optimal CSC and delirium incidence after discharge or transfer in older adults were evaluated.

## Methods

### Study population

All data collection in the TRADE observational study was carried out prospectively after the participants had been enrolled and given their informed consent. The study was conducted from August 2019 to February 2020 in four acute care hospitals in Baden-Wuerttemberg (southwest Germany): three university hospitals (Heidelberg, Tuebingen, Ulm) and one academic geriatric hospital (Heidelberg). In the university hospitals, the following departments were participating: cardiology (Heidelberg), cardiology and gastroenterology (Tuebingen), gastroenterology and trauma surgery (Ulm). All patients of the participating hospital wards aged 70 years or older without delirium and planned to be discharged or transferred within the next four days received information about the study contents and aims and were asked for participation together with their primary caregivers. Depending on patients’ capacity for consent, their legally authorized representatives were contacted, informed, and asked for the patient’s participation. Legally authorized representatives could be either legal guardians (often professional guardians) or persons with power of attorney (usually relatives). If no legally authorized representatives were available, patients had to be excluded from participation in the study. Further exclusion criteria were palliative therapy with an estimated survival time of less than three months and a too large distance between the study center and place of residence to be able to carry out the follow-up visits. In addition, potential participants were excluded if, in the opinion of the study staff and after consultation with the ward team, it would not have been possible to administer a standardized general questionnaire or the MoCA test because they were too cognitively impaired.

**Fig. 1:**
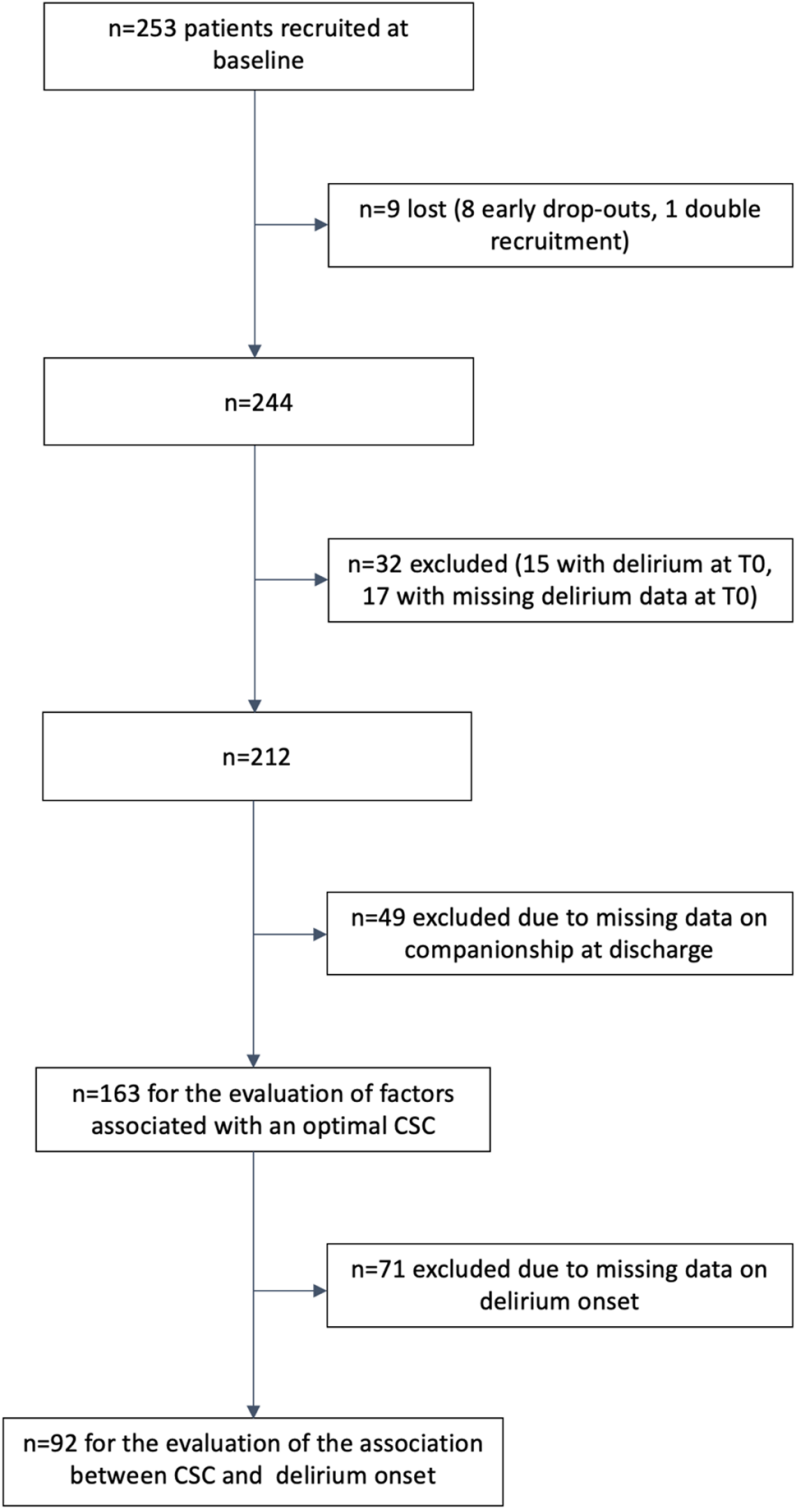
Flow chart of the study population

Initially, the recruitment of 600 participants was planned, which followed a case number calculation based on the discharge of ≥ 70-year-olds in the four recruiting centers during the previous year, leading to an expected number of 150 patients/study center for the scheduled period of 6 months. No pilot study was conducted prior to the start of the multicenter observational study. In the context of the onset of the coronavirus pandemic, a premature termination of recruitment in February 2020 was requested and approved by the sponsor. Overall, and based on the inclusion criteria, a total of 1,616 patients were considered for participation across the four study centers. Of these, *N* = 253 were eventually recruited for participation in the study. Reasons for non-inclusion in the study were the presence of exclusion criteria, or that potentially suitable patients refused to participate in the study. Less than 0.7% of the individuals screened could not be included due to cognitive impairment that was too severe to conduct a survey. Among the 253 participants, 8 became early drop-outs, and one had been recruited twice and therefore had to be excluded from the analyses. The presence of delirium at the time of enrollment (timepoint T0) defined by a positive evaluation through the International Classification of Diseases (ICD)-10 adapted Confusion Assessment Method-Severity (I-CAM-S) [3, 26] or the Nursing Delirium Screening Scale (Nu-DESC) [27, 28] led to exclusion from the analyses in 15 persons. In a further 17 persons, the information regarding the presence of delirium at the time of enrollment was incomplete, leading to their exclusion from the analyses, and leaving *n* = 212 participants. Of these, *n* = 49 had to be excluded due to missing data concerning information on companionship at hospital discharge or transfer, leading to a study population of *n* = 163 patients for the evaluation of the factors associated with CSC. Primarily due to the SARS-CoV-2 pandemic and the associated contact restrictions, which made it impossible to conduct home visits or personal questionings in rehabilitation clinics and nursing homes during several periods, no data with respect to the onset of delirium after discharge or transfer (timepoints T1/T2) could be collected for 71 patients, so that the association between CSC and the onset of delirium could be evaluated only among 92 patients (Fig. 1).

### Study design

**Fig. 2:**
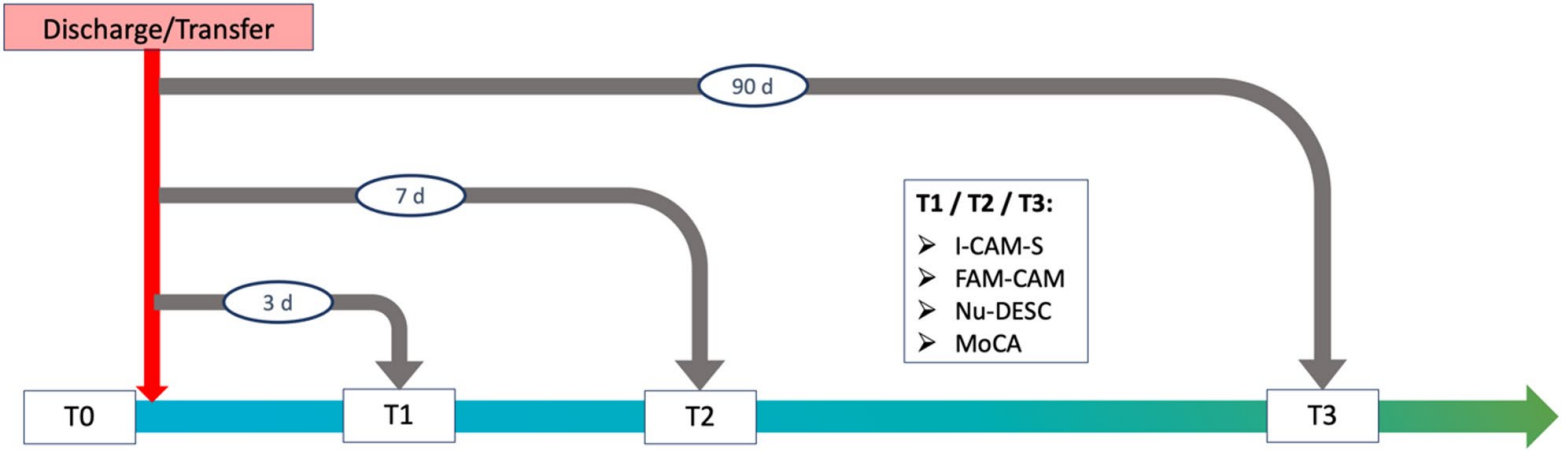
Study timepoints and instruments for delirium detection and cognitive assessment

The TRADE observational study was a prospective multicenter cohort study. All participants and their caregivers were examined using standardized questionnaires and tests at four different timepoints: the day before discharge or transfer (T0), and on days 3 (T1), 7 (T2) and 90 (T3) after discharge or transfer (Fig. 2). The questionnaires administered at time points T0, T1, T2, and T3 represent a mixture of validated instruments and self-developed, study-specific standardized questions and were used to assess companionship, delirium, and all covariates (Additional file 2). All follow-up examinations at T1 and T2 were conducted in person by home visits. After the start of the SARS-CoV-2 pandemic in 03/2020, the outstanding T3 examinations (*n* = 64) were carried out using a modified telephone T3 questionnaire in order to reduce personal contacts as recommended by the authorities. The caregivers were questioned by telephone at all three follow-up timepoints (T1, T2, T3). Nursing staff was questioned in person at timepoint T0, and at timepoints T1, T2 and T3, if present. The last follow-up examination took place in May 2020. Written informed consent was obtained from all participants (patients/legally authorized representatives as well as caregivers). The study was conducted according to the guidelines of the Declaration of Helsinki. The ethics commissions of Ulm (# 84/19), Heidelberg (# S-443/2019) and Tuebingen (# 352/2019BO2) universities provided ethical approval. Reporting was based on the Strengthening the Reporting of Observational studies in Epidemiology (STROBE) guidelines for epidemiological studies [29]. The completed STROBE checklist is available in the supplementary material (Table S1, Additional file 1).

### Evaluation of companionship

Companionship, defined as the presence of a familiar person (relative or caregiver) at the time of discharge or transfer, was evaluated by a questionnaire conducted three days after discharge from hospital or transfer to another institution or department. Participants as well as caregivers were asked about the presence, type and length of companionship by the patient’s primary caregiver or another person directly *before*,* during*,* and/or after* transport from the hospital to their home or another place they had been discharged or transferred to. Optimal companionship, allowing a seamless transition between health care sectors, was identified if the caregivers were present (i) *before and after* transport or (ii) *before*,* during and after* transport.

### Delirium assessment

Considering the fluctuating character of delirium and the challenges in diagnosis, delirium detection was based on direct evaluation by the trained assessors as well as on standardized questionnaires administered to caregivers and nursing staff, if available, at T1 and T2. For this purpose several instruments were used: The I-CAM-S [26] is a tool assessing acute onset and/or fluctuation of symptoms, attention, thinking ability, level of consciousness, and psychomotor activity. Delirium is suspected if there is at least (i) an acute onset and/or fluctuating course, (ii) a disturbance of attention and (iii) disorganized thinking or a change in consciousness. The interpretation thus follows the algorithm of the established CAM with a sensitivity of 0.94–1.00 and a specificity of 0.90–0.95 [3]. The Family Confusion Assessment Method (FAM-CAM) is a CAM-derived instrument consisting of 7–11 items in the form of a standardized questionnaire for use with primary caregivers, which comprises information on symptoms present during the period since the last questioning. As with I-CAM-S, delirium is suspected if the described CAM algorithm is fulfilled. For FAM-CAM the sensitivity, compared to the CAM, is 0.88, the specificity 0.98 [30]. The Nu-DESC is a five-item instrument rating nurses’ observations concerning disorientation, inappropriate behavior and communication, hallucinations and psychomotor retardation over a 24-hour period, indicating suspected delirium by a result of ≥ 2 points, which requires the presence of at least one of the five symptoms mentioned in severe form or at least two symptoms in moderate form. For Nu-DESC a sensitivity of 0.86 and a specificity of 0.87, compared to the CAM, have been reported [27]. The diagnostic accuracy and psychometric properties of delirium screening instruments, including CAM-based tools and Nu-DESC, have been summarized in recent systematic reviews and meta-analyses, supporting their validity and reliability in clinical settings [31, 32]. The FAM-CAM and Nu-DESC instruments offer the advantage of being focused on a period rather than a specific point in time, thus ensuring that any symptoms occurring between the follow-up appointments were identified. All instruments were applied by trained assessors during the questionings. The previous training course comprised 12 training hours. To ensure a standardized administration of the questionnaires, regular telephone and video conferences were held, with assessors from all centers participating.

### Delirium definition

Incident delirium seven days after discharge or transfer was defined by a positive evaluation through I-CAM-S or FAM-CAM or Nu-DESC at T1 or T2. No incident delirium after discharge or transfer could be assumed if at least FAM-CAM or Nu-DESC at T2 was completed and the available delirium assessments at T1 and T2 were negative.

### Covariates

The covariates listed are commonly used to characterize a population of older adults or were selected based on their theoretical relevance to social support and delirium risk. The following variables were evaluated as possible predictors for optimal CSC: age, sex, school education (≤ 10 years, > 10 years), native language (German, others), migration background (yes, no), body mass index, blood pressure, smoking status (non-smoker, ex-smoker, current smoker), alcohol consumption (never, formerly, currently); sociodemographic characteristics like marital status (married/living in partnership, single, divorced/separated, widowed), housing (own household, other household, assisted living), living alone (yes, no), children (yes, no), grandchildren (yes, no), social contacts (spouse, sister, brother, son, daughter, son-in-law, daughter-in-law, friends, neighbors), social support assessed by the Lubben Social Network Scale (LSNS)-6 [33]; physical function, including mobility assessed by the Rivermead Mobility Iindex [34], Basic and Instrumental Activities of Daily Living (BADL/IADL) assessed by the Barthel [35] and Lawton Index [36], respectively, subjective health status (poor/fair, good/very good/excellent), sensory function (hearing problems (yes, no), vision problems (yes, no) including visual acuity test [37]), falls during the last three months (yes, no), use of walking aids (yes, no), care level (yes, no), frailty assessed by the Canadian Study of Health and Aging (CSHA) Clinical Frailty Scale (CFS) [38]; depression/anxiety assessed by the Patient Health Questionnaire (PHQ)-4 [39]; comorbidities assessed by an extended Charlson Comorbidity Index (CCI) [40]; cognition assessed by Montreal Cognitive Assessment (MoCA) [41]; subjective memory impairment (yes, no, unknown) [42], history of previous delirium (yes, no, unknown); length of hospital stay; discharge setting defined as "known" if the patient had been living there for at least six months prior to hospitalization, otherwise "unknown"); mode of transportation at discharge/transfer (patient/disabled transport ambulance, car/taxi/bus/train/tram). A modified Delirium Risk Assessment Score (DRAS) [43], consisting of the variables age (≥ 75 years), alcohol consumption (daily), cognition (MoCA < 26 / blind version < 18), ADL/mobility (Barthel 1.-8. < 80 or use of walking aid), vision/hearing problems, number of comorbidities (> 2), previous delirium was also calculated for each participant.

### Statistical analysis

Descriptive statistics are provided for the overall study population as well as stratified according to the type of companionship (optimal versus not-optimal CSC). Univariate analysis allowed the identification of those variables associated with optimal CSC (p-value < 0.1). The use of *p* < 0.1 in the univariate preselection aimed at a sensitive, more inclusive selection for multivariable model building in an exploratory study. The level of correlation between the identified variables was evaluated in order to avoid collinearity. Collinearity was assumed if the correlation coefficient was < -0.3 or > 0.3 and the p-value was < 0.05. Variables that were more clinically relevant and/or statistically significant were retained in the selection, while correlating variables that were less clinically relevant/statistically significant were excluded. The number of variables to be considered as possible predictors for CSC was accordingly reduced. The 7-days incidence proportion and its 95% confidence interval (CI) were calculated. The number of missing data was listed in a corresponding table column (number of missings) in the descriptive analyses. Logistic regression analysis using stepwise backwards selection was performed for the identification of the final predictors for optimal CSC.

Lastly, logistic regression analysis was used to evaluate the association between optimal CSC and 7-days delirium incidence unadjusted (Model 1), as well as adjusted for study center and for the baseline delirium risk defined through the modified DRAS (Model 2), providing the odds ratios (OR) with their respective 95% CI. For the description of the predictive accuracy, the C-index was calculated for both models. A possible interaction between CSC and discharge environment was modelled and investigated applying an interaction term (p-value < 0.2). To account for potential sources of bias, comparisons of participant characteristics were stratified by the four recruiting centers, by included versus excluded participants, and by mode of transportation. Calculations were carried out using the statistical software SAS 9.4.

## Results

### Descriptive analyses of patient characteristics

Overall, 90 participants had to be excluded from the analyses for various reasons (Fig. 1). The remaining study population of 163 patients had a median age of 80.8 years (range 70.0–96.6 years), with 91 (55.8%) being female. Of these, 66 (40.5%) had been recruited at Ulm University Hospital, 38 (23.3%) at the geriatric Bethanien Hospital Heidelberg, 28 (17.2%) at Heidelberg University Hospital, and 31 (19.0%) at Tuebingen University Hospital. Further characteristics of the study population are shown in Table 1.

**Table 1:** Characteristics of study population with information on companionship (*n* = 163)

Variable	Study population with information on companionship (*n* = 163)	Number of Missings
Age (years), Median (Min, Q1, Q3, Max)	80.8 (70.0, 77.0, 84.2, 97.6)	0
Women, n (%)	91 (55.8)	0
School education, n (%)		0
≤ 10 years	112 (68.7)	
> 10 years	51 (31.3)	
Migration background, n (%)	20 (12.3)	0
Center		0
Ulm University Hospital	66 (40.5)	
Heidelberg Geriatrics	38 (23.3)	
Heidelberg University Hospital	28 (17.2)	
Tuebingen University Hospital	31 (19.0)	
Systolic blood pressure (mmHg) on admission, Median (Min, Q1, Q3, Max)	130 (86, 115, 145, 190)	9
Diastolic blood pressure (mmHg) on admission, Median (Min, Q1, Q3, Max)	70 (40, 65, 80, 109)	9
Smoking status, n (%)		0
non-smoker	85 (52.2)	
ex-smoker	73 (44.8)	
current smoker	5 (3.1)	
Alcohol consumption, n (%)		4
never	39 (24.5)	
formerly	33 (20.8)	
currently	87 (54.7)	
Marital status, n (%)		0
married/partnership	88 (54.0)	
single	10 (6.1)	
divorced/separated	5 (3.1)	
widowed	60 (36.8)	
Children, n (%)	146 (89.6)	0
Grandchildren, n (%)	119 (73.0)	0
Current housing situation, n (%)		0
own household	153 (93.9)	
household of children/ grandchildren/ other relatives	1 (0.6)	
assisted living	9 (5.5)	
Living alone, n (%)	74 (45.4)	0
Lubben Social Network Scale		10
Median (Min, Q1, Q3, Max)	16 (0, 11, 21, 30)	
social isolation (< 12), n (%)	40 (26.1)	
social support (≥ 12), n (%)	113 (73.9)	
Social contact with …, n (%)		
spouse	90 (55.2)	0
sister	69 (42.3)	0
brother	64 (39.5)	1
daughter	94 (57.7)	0
son	96 (58.9)	0
daughter-in-law	71 (43.6)	0
son-in-law	72 (44.2)	0
daughter and/or son-in-law		0
one of both	24 (14.7)	
both	71 (43.6)	
friend	141 (86.5)	0
neighbor	143 (89.4)	3
Barthel Index, Median (Min, Q1, Q3, Max)	85 (0, 57.5, 100, 100)	11
Hearing problems, n (%)	81 (49.7)	0
Vision problems, n (%)	94 (57.7)	0
Fall during last 3 months, n (%)	79 (48.5)	0
Walking aid, n (%)	85 (52.2)	0
CSHA Clinical Frailty Scale (1 = very fit, 9 = terminally ill), median (Min, Q1, Q3, Max)	4 (1, 3, 6, 8)	2
Subjective general health, n (%)		8
poor/fair	83 (53.6)	
good/very good/excellent	72 (46.5)	
Subjective mental health, n (%)		9
poor/fair	30 (19.5)	
good/very good/excellent	124 (80.5)	
Depression in PHQ-4, n (%)	41 (26.5)	8
Anxiety in PHQ-4, n (%)	27 (17.5)	9
Comorbidities		
Cardiovascular diseases (heart attack, coronary heart disease, valvular heart diseases, heart failure, cardiac arrhythmia, peripheral artery occlusive disease, other arterial disorders), n (%)	120 (73.6)	0
Hypertension, n (%)	119 (73.0)	0
Diabetes mellitus, n (%)	46 (28.2)	0
Chronic lung disease, n (%)	28 (17.2)	0
Sleep apnea syndrome, n (%)	9 (5.5)	0
Malignant tumor disease, n (%)	42 (25.8)	0
Depression, n (%)	36 (22.1)	0
Stroke, n (%)	28 (17.2)	0
Paralysis, n (%)	14 (8.6)	0
Traumatic brain injury, n (%)	15 (9.2)	0
Dementia, n (%)	15 (9.2)	0
Parkinson’s disease, n (%)	4 (2.5)	0
Cerebral hemorrhage, n (%)	6 (3.7)	0
CCI, Median (Min, Q1, Q3, Max)	2 (0, 1, 4, 10)	0
Relevant diseases (circulatory insufficiency or diabetes mellitus or blood cancer or epileptic seizures), n (%)	92 (56.4)	0
Number of medicines, Median (Min, Q1, Q3, Max)	9 (2, 7, 12, 20)	0
Polymedication (≥ 5 medicines), n (%)	152 (93.3)	0
Cognition		
MoCA		
Blind version, n (%)	5 (3.1)	
Sum score (*without* blind), Median (Min, Q1, Q3, Max)	22 (7, 19, 24, 29)	26
Sum score (*only* blind), Median (Min, Q1, Q3, Max)	17 (9, 13, 17, 22)	0
Conspicuously^1^ (incl. blind), n (%)	119 (86.9)	26
Subjective memory impairment, n(%)		0
yes	96 (58.9)	
no	64 (39.3)	
unknown	3 (1.8)	
History of previous delirium, n (%)		0
yes	24 (14.7)	
no	135 (82.8)	
unknown	4 (2.5)	
DRAS, Median (Min, Q1, Q3, Max)	7 (1, 5, 8, 12)	10
Discharge/transfer (T1)		
Length of hospital stay (days), Median (Min, Q1, Q3, Max)	8 (1, 4, 17, 61)	1
Discharge environment, n (%)		0
unknown (been there < 6 months)	53 (32.5)	
of these n (%) never been there	44 (83.0)	
known (been there ≥ 6 months)	110 (67.5)	
of these n (%) home	108 (98.2)	

^1^ Defined as sum < 26 (normal version) and sum < 18 (blind version)

Abbreviations: *CSHA* Canadian Study on Health and Aging, *PHQ-4* Patient Health Questionnaire-4, *CCI* Charlson Comorbidity Index, *MoCA* Montreal Cognitive Assessment, *DRAS* Delirium Risk Assessment Score

With regard to the discharge setting, 110 patients were discharged to a known environment and 53 patients to an unknown environment. Of the latter, 19 were transferred within the hospital (3 to other hospital departments, 16 to rehabilitation, which was located in the same building) and the remaining 34 to other institutions. The most considerable differences between the participants from the four centers were found in age, sex, social environment, functionality/frailty, comorbidities, cognition and length of hospital stay. Participants from the geriatric hospital were noted to be older on average, more often living alone and socially isolated, with the lowest indices for mobility and ADL, higher scores for frailty and CCI, a lower MoCA score, a higher risk for delirium according to the DRAS score, and a longer length of stay. The characteristics of the study population stratified for study centers are shown in detail in the supplementary material (see Table S2, Additional file 1). Compared to the study population (*n* = 163), those with missing data on companionship (*n* = 49) were noted to be more often single or divorced, used more often walking aids, reported more often a poor/fair health status, had less often children, and had a longer hospital stay (see supplementary Table S3, Additional file 1).

### Optimal versus not-optimal cross-sectoral companionship

Table 2 presents the types of companionship reported by the study population. Only three (1.8%) patients reported no companionship at all. Optimal CSC was observed among 61 (37.4%) patients. The descriptive analysis comparing the groups with optimal CSC versus not-optimal CSC showed the following differences: Those with optimal CSC were noted to be more often male, have been recruited in the study center Heidelberg University Hospital, be currently smoking and consuming alcohol, be married or living in a partnership, have children, grandchildren, social contact with their spouse, daughter, son-in-law and neighbors. On the other hand, those with no or not-optimal CSC were more likely to live alone, to show anxiety in the PHQ-4, and to have one or more of the four comorbidities circulatory insufficiency, diabetes mellitus, blood cancer and epileptic seizures. They had a shorter length of hospital stay, were more often discharged to an unknown environment, and more often transported by patient transport ambulance. Further details can be found in the supplementary material (see Table S4, Additional file 1).

**Table 2:**
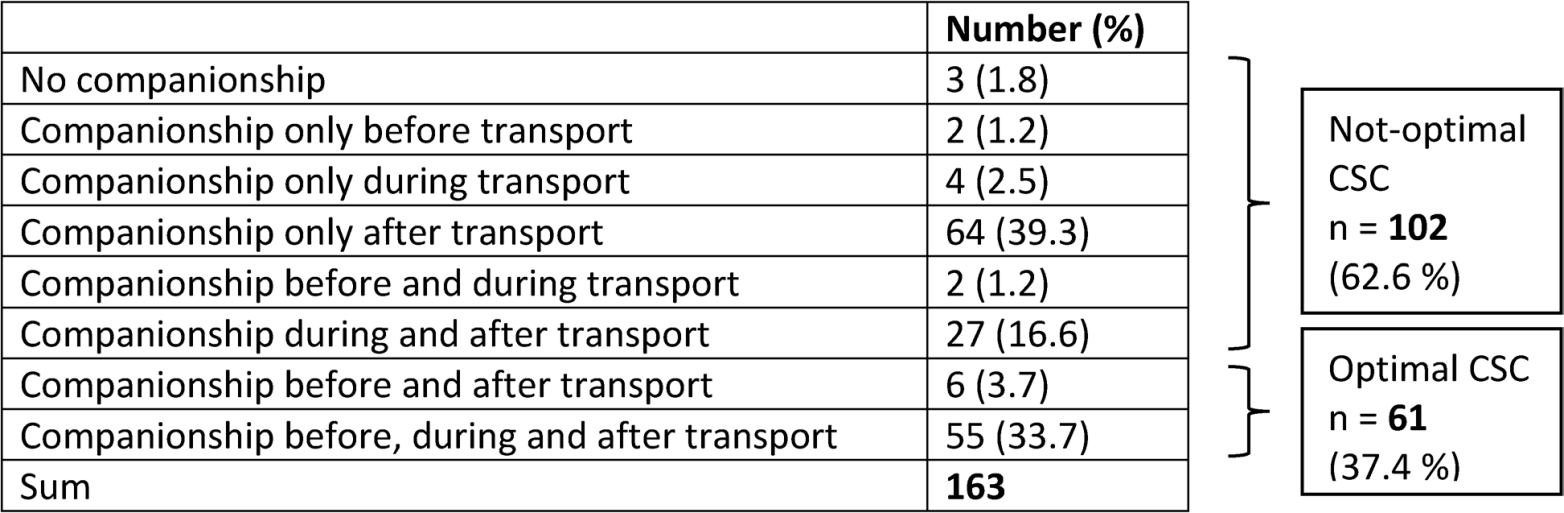
Type of companionship identified at the time of discharge/transfer

### Predictors for optimal cross-sectoral companionship (CSC)

The correlation analysis of the total of 23 variables with significant differences between the groups with optimal vs. not-optimal CSC yielded 23 statistically significant correlations. By deleting collinear variables, the number of variables to be considered as possible predictors for optimal CSC was reduced to the following: sex, center, smoking, alcohol consumption, living alone, social contact with daughter and/or son-in-law, social contact with neighbor, anxiety, length of hospital stay, and mode of transportation. Four comorbidities were associated with the type of CSC, both individually and collectively as the variable “relevant diseases”: poor circulation, diabetes mellitus, blood cancer, or epileptic seizures (see supplementary Table S5, Additional file 1). Due to missing values for the identified variables, the final data set comprised *n* = 146 (54 (37.0%) with optimal CSC, 92 (63.0%) with not-optimal CSC).

After performing backwards selection, the following four variables were identified as predictors for optimal CSC: Former alcohol consumption, social contact with daughter/son-in-law, length of hospital stay and mode of transportation. Former alcohol consumption and transport with ambulance reduced the odds for optimal CSC (OR 0.26 [95% CI 0.09; 0.78] and 0.25 [95% CI 0.10; 0.66], respectively), while social contact with daughter and son-in-law as well as longer length of hospital stay increased them (OR 3.37 [95% CI 1.44; 7.90] and 1.07 [95% CI 1.02; 1.12], respectively). The C-index was 0.743, indicating a good predictive accuracy of the model (Table 3).

**Table 3:** Final logistic regression model for predictors of optimal cross-sectoral companionship (*n* = 146)

Variable		OR [95% CI]
Alcohol consumption	currently	ref.
	formerly	**0.26 [0.09**;** 0.78]**
	never	0.59 [0.24; 1.45]
Social contact with daughter/son-in-law	no	ref.
	with one of both	2.01 [0.66; 6.06]
	with both	**3.37 [1.44**;** 7.90]**
Length of hospital stay		**1.07 [1.02; 1.12]**
Mode of transportation	car/taxi/bus/train/tram	ref.
	patient/disabled transport ambulance	**0.25 [0.10**;** 0.66]**
**C-index 0.743**		

### Secondary analysis

Since the mode of transportation proved to be a strong negative predictor for optimal CSC (OR 0.25 [95% CI 0.10; 0.66]), due to the fact that most patient transport companies generally refused to allow accompanying persons, especially during the SARS-CoV-2 pandemic, this aspect was examined in more detail. For this purpose, we analyzed predictors for optimal CSC among those not transported by ambulance or any other patient transport company. Of the *n* = 95 discharged or transferred by car, taxi or public transportation, 42 (44.2%) were identified having optimal CSC. Group differences compared to persons transported by ambulance can be found in Table S6, Additional file 1.

Former alcohol consumption reduced the odds for optimal CSC (OR 0.17 [95% CI 0.04; 0.75]), while social contact with daughter and son-in-law as well as longer length of hospital stay increased statistically significantly the odds of having an optimal CSC (OR 3.07 [95% CI 1.08; 8.74] and 1.13 [95% CI 1.04; 1.23], respectively). Living alone was identified as a further predictor indicating a reduction of the odds for optimal CSC, although it proved not to be significant (OR 0.41 [95% CI 0.14; 1.20]). The model showed a good predictive accuracy with a C-index of 0.765 (Table 4).

**Table 4:** Secondary analysis, final logistic regression model for predictors of optimal cross-sectoral companionship among those not transported by ambulance or any other patient transport company (*n* = 95)

Variable		OR [95% CI]
Alcohol consumption	currently	ref.
	formerly	**0.17 [0.04**;** 0.75]**
	never	0.77 [0.24; 2.46]
Social contact with daughter/son-in-law	no	ref.
	with one of both	2.27 [0.52; 9.90]
	with both	**3.07 [1.08**;** 8.74]**
Length of hospital stay		**1.13 [1.04**;** 1.23]**
Living alone		0.41 [0.14; 1.20]

**C-index 0.765**

### Association of companionship and delirium

Further 71 participants had to be excluded from the analyses due to missing delirium data (Fig. 1). Among 92 participants a total of 19 delirium cases could be detected (incidence proportion 20.7% [95% CI 13.6; 30.0]). Overall, 5 (26.3%) delirium cases were identified by I-CAM-S. The other 14 cases were detected by the FAM-CAM (*n* = 8), the Nu-DESC (*n* = 5), and the FAM-CAM plus Nu-DESC (*n* = 1) (see supplementary Table S7, Additional file 1). Details on the timing of the positive results and the respective decisive instruments can be found in the supplementary Table S8, Additional file 1. There was a higher delirium incidence proportion among those discharged to an unknown setting compared to those discharged to known settings (Table 5). A comparison of delirium incidence in the four study centers is included in the supplement (Table S9, Additional file 1), with the highest incidence observed among those patients discharged from the geriatric hospital.

**Table 5:** 7-days delirium incidence proportion (overall and stratified by discharge setting)

Study population with information on companionship and delirium (*n* = 92)	Delirium cases/*n*	7-days delirium incidence proportion (%) [95% CI]
**Overall**	19/92	20.7 [13.6; 30.0]
Discharge to unknown setting	12/44	27.3 [16.3; 41.8]
Discharge to known setting	7/48	14.6 [7.2; 27.2]

There was no statistically significant difference in the onset of delirium between those with optimal CSC versus those with not-optimal CSC (*n* = 8 (25.8%) versus *n* = 11 (18.0%)). The multivariable logistic regression showed a non-significant association between CSC and the onset of delirium after discharge/transfer even after adjustment for the modified DRAS [43], representing the baseline delirium risk, and center (OR 1.87 [95% CI 0.60; 5.85]) (see supplementary Table S10, Additional file 1). The examination of an interaction between CSC and discharge environment revealed no evidence of effect modification (*p*-value > 0.2).

## Discussion

The TRADE observational study is the first one evaluating the current state of companionship and incidence of delirium after discharge from hospital in German settings, as well as examining possible predictors of optimal cross-sectoral companionship (CSC) and its association with delirium incidence after discharge/transfer in hospitalized older adults in Germany. Our results showed a clinically relevant delirium incidence proportion with about one in five older adults experiencing delirium after discharge or transfer. An optimal CSC, where a cross-sectoral exchange of information is supported by the presence of relatives or other caregivers, could only be found in 37% of the patients. The identified predictors for an optimal companionship (former alcohol consumption, social contact with daughter and son-in-law, length of hospital stay and mode of transportation) were confirmed and expanded by the negative predictor “living alone” in the secondary analysis. This gives rise to various considerations. On the one hand, the social network plays an important role in determining whether or not someone receives optimal companionship upon discharge. Individuals with a history of regular alcohol consumption may have a less developed social network, resulting in a shortage of potential support persons. Contact with a daughter and/or son-in-law could indicate that, among family members, it is primarily daughters who are concerned and organize support. In addition, the length of the stay is related to the severity of the illness, which could also lead to family members being more likely to perceive the need for support. On the other hand, it is shown that transport by ambulance upon discharge is an obstacle to optimal companionship, representing a potentially modifiable predictor. The establishment and maintenance of social relationships should be given appropriate consideration. The fact that only about a third of patients (37%) received optimal CSC upon discharge or transfer indicates the need to develop strategies to inform everyone involved in patients’ care (hospital staff, relatives/caregivers) about the importance of accompanying and supporting older patients during this time. Companies that offer patient transport services could become more open to allowing accompanying persons to travel with patients, in the knowledge that this improves the situation for older patients.

The literature on the subject of companionship at discharge of older people is scarce. A qualitative study on older single people accompanied by volunteers after discharge found that companionship contributed to facilitating and enriching the older persons’ daily life [16]. An Australian research team conducted interviews with family caregivers and community-living, cognitively impaired older people whom they had taken care of around their discharge from hospital and found that caregivers were able to provide an important safety net by, inter alia, communicating discharge information and coordinating further care [17]. A recent study in a German trauma surgery department showed how volunteer transition companions successfully support older people prior to and after discharge in organizing their home environment and their social and medical care [18]. A systematic review evaluating the effectiveness of transitional care, which included Comprehensive Geriatric Assessment, home visits, or telephone calls, showed a reduction in readmission rates after 6 months. However, the interventions were carried out by healthcare professionals [44].

Our study showed a clinically relevant delirium incidence proportion with about one in five older adults experiencing delirium after discharge or transfer, highlighting the public health importance of these results. So far, there is a lack of information with respect to the incidence of delirium in older adults after discharge. Few articles discuss the issue of persistent delirium after discharge or delirium prevalence upon admission to a nursing home [45, 46], and also consider this in relation to the degree of frailty in the discharged persons [47]. In the context of transfers within the hospital it has been shown that the number of room transfers is associated with increased delirium incidence [7]. Consistent with our results, a randomized controlled trial examining delirium incidence during rehabilitation at home versus in-hospital rehabilitation after discharge from an acute hospital found lower odds of developing delirium during rehabilitation among those in the home group (OR 0.17 [95% CI 0.03; 0.65]) [48]. This points out the importance of considering the type of setting after discharge (known versus unknown) when estimating delirium risk.

### Strengths and limitations

The TRADE observational study has several strengths and limitations. Participants were recruited in four different settings in southern Germany, and the assessments carried out included numerous physical, functional, cognitive and social aspects that enabled a comprehensive characterization of the study population.

Overall, participants were quite heterogeneous between centers. In this context, no delirium was detected in Heidelberg University Hospital. A possible reason might be that the included wards represented highly selected patients undergoing predominantly elective invasive diagnostic procedures in the cardiology unit. These patients were among the physically and functionally fittest, mostly married or living in a partnership, and almost exclusively discharged to their homes (for details see supplementary Table S2, Additional file 1). Nevertheless, or precisely because of this, the TRADE study population adequately represented all older patients encountered in in-patient settings, from relatively fit to very frail older people. This could be seen as a strength, as it enabled CSC to be examined in various contexts.

The observed 7-days incidence of 20.7% can be explained by the use of a combined detection method, covering information over longer periods of time and thus better capturing potentially short-term occurrences of delirium symptoms and fluctuations, meaning that this strategy for detecting delirium in outpatient settings can be considered a strength. Due to the fluctuation of symptoms, the application of the I-CAM-S with a maximum duration of five minutes provides only a ‘snap-shot’ and therefore bears the risk of missing one or more of the CAM core criteria. This issue is corroborated by the fact that only a quarter of the delirium cases in this study were identified by I-CAM-S. The remaining three-quarters of the cases were detected by the FAM-CAM and the Nu-DESC, both instruments that cover a longer period of time. The triple methodology used in delirium assessment could have led to a better identification of delirium, and, as a result, the observed high incidence. The inclusion of geriatric patients with a high baseline risk for delirium could also be partly responsible for the high incidence of delirium.

A general limitation was the fact that the recruitment period was affected by the beginning of the SARS-CoV-2 pandemic, forcing the premature termination of recruitment in February 2020, 6 weeks earlier than planned, compromising the statistical power. In addition, there were further challenges such as a lack of communication regarding planned discharges, and a higher rejection rate than anticipated. Surveys with patients’ caregivers and nursing staff turned out to be a challenge, especially during the SARS-CoV-2 pandemic. Though repeated contact attempts were undertaken, in many cases the caregivers were unavailable or refused to answer questionnaires despite of their previous consent. The nursing staff at the hospital sometimes stated that they were unable to answer the Nu-DESC items due, for example, to frequent staff changes or assignments to other wards, or to insufficient knowledge about the patients to be able to provide information about the behaviors in question. In the case of those patients who were discharged home, nursing staff were often not present at the follow-up examination. These circumstances resulted in numerous missing questionnaire data, which significantly reduced the amount of data available for analysis. In addition, there were some differences between the participants who were included in the analyses and those who were excluded due to missing data, with those included noted to be fitter with shorter lengths of stay. This should therefore be considered a potential source of attrition and sampling bias. The exclusion of individuals with severe cognitive impairment also does not allow to evaluate the research question in this high risk group, and may introduce a source of further bias. These limitations may have affected the estimation of delirium incidence and the analysis of the association between optimal CSC and the occurrence of delirium in older people discharged from hospital.

Overall, and looking ahead, it can be said that the concept and types of companionship are complex. While qualitative interviews with hospital and transport staff were conducted in parallel to the TRADE observational study and provided contextual insights into discharge and transport processes [25], systematic qualitative exploration of patients’ and caregivers’ perspectives was not part of the present analysis. Future qualitative studies focusing explicitly on these perspectives could therefore provide highly interesting additional insights. The variable “feeling ready for discharge”, reflecting the subjective patient’s view, could also be an interesting predictor, but was not systematically collected in TRADE. For future studies, collecting this aspect would certainly be recommended as a valuable addition. Further research should also examine the influence of clearly defined companionship measures on the incidence of delirium. Readmissions were not specifically recorded as a delirium risk factor in this study. However, they were assessed in the follow-up survey after three months and could therefore be examined as a secondary endpoint in future analyses.

## Conclusion

The data obtained show that optimal cross-sectoral companionship occurs only in about one third of discharges or transfers of hospitalized older adults. Contact with daughter and son-in-law and length of hospital stay are positive predictors that increase the odds for optimal cross-sectoral companionship, while former alcohol consumption and transportation by patient transport ambulance decrease them. Among those without utilization of ambulance services, “living alone” was identified as a possible additional negative predictor of optimal cross-sectoral companionship. A 7-days delirium incidence proportion of public health significance was observed, affecting an average of one in five discharged patients, with a higher incidence proportion when patients are discharged/transferred to an unknown setting. Unfortunately, the statistical power was not sufficient to appropriately examine the association between cross-sectoral companionship and 7-days delirium incidence. Nevertheless, our results strengthen the possibility of implementing caregiver-delivered delirium-preventive measures in the context of discharge or transfer, which has been examined in the TRADE pilot intervention study. Furthermore, they can contribute to promoting social contacts, raising awareness of the importance of supporting older people during discharge, and ultimately improving continuity of care during the transition from inpatient to outpatient care.

## Supplementary Information

Supplementary Material 1: Additional file 1. Table S1. STROBE checklist. Table S2. Characteristics of study population stratified by study centers (n=163). Table S3. Characteristics of included vs. excluded participants (available vs. missing information on companionship during discharge/transfer). Table S4. Characteristics of study population (n=163) stratified according to the type of CSC. Table S5. Selected and excluded variables for logistic regression. Table S6. Characteristics of study population stratified according to mode of transportation (car/taxi/bus/train/tram vs. patient/disabled transport ambulance) (n=146). Table S7. I-CAM-S, FAM-CAM and Nu-DESC in the total study population (n=212). Table S8. Delirium detection at T1 and T2 with different instruments. Table S9. 7-days delirium incidence proportion stratified by study centers. Table S10. Logistic regression evaluating the association between companionship and 7-days delirium incidence.

Supplementary Material 2: Additional file 2. Study questionnaire.

## Data Availability

The data supporting the conclusions of this article are included within the article or its additional file 1. The datasets generated and analyzed which are not included in this article or the additional file 1 are available from the corresponding author on reasonable request.

## Abbreviations

ADL: Activities of Daily Living
BADL: Basic Activities of Daily Living
CAM: Confusion Assessment Method
CCI: Charlson Comorbidity Index
C-index: Concordance index
CFS: Clinical Frailty Scale
CI: Confidence interval
CSC: Cross-sectoral companionship
CSHA: Canadian Study of Health and Aging
DNQP: Deutsches Netzwerk für Qualitätsentwicklung in der Pflege (German network for quality development in nursing)
DRAS: Delirium Risk Assessment Score
DRKS: Deutsches Register klinischer Studien (German Clinical Trials Register)
FAM-CAM: Family confusion assessment method
I-CAM-S: International classification of diseases-10 adapted Confusion Assessment Method-Severity
IADL: Instrumental Activities of Daily Living
ICU: Intensive care unit
LSNS: Lubben Social Network Scale
MoCA: Montreal Cognitive Assessment
Nu-DESC: Nursing Delirium Screening Scale
OR: Odds ratio
PHQ-4: Patient Health Questionnaire-4
SARS-CoV-2: Severe acute respiratory syndrome coronavirus type 2
SAS: Statistical analysis system
STROBE: STrengthening the Reporting of OBservational studies in Epidemiology
T0/1/2/3: Timepoint 0/1/2/3
