# Supplementary material for: Companionship at hospital discharge and its association with subsequent delirium onset in older adults – the TRADE observational study
Source: BMC Geriatr. 2026 Feb 21;26:370. doi: 10.1186/s12877-026-07194-3 (PMC12997767; doi:10.1186/s12877-026-07194-3)
Supplement: Supplementary file 1 — Supplementary Material 1: Additional file 1. Table S1. STROBE checklist. Table S2. Characteristics of study population stratified by study centers (n=163). Table S3. Characteristics of included vs. excluded participants (available vs. missing information on companionship during discharge/transfer). Table S4. Characteristics of study population (n=163) stratified according to the type of CSC. Table S5. Selected and excluded variables for logistic regression. Table S6. Characteristics of study population stratified according to mode of transportation (car/taxi/bus/train/tram vs. patient/disabled transport ambulance) (n=146). Table S7. I-CAM-S, FAM-CAM and Nu-DESC in the total study population (n=212). Table S8. Delirium detection at T1 and T2 with different instruments. Table S9. 7-days delirium incidence proportion stratified by study centers. Table S10. Logistic regression evaluating the association between companionship and 7-days delirium incidence. [file 12877_2026_7194_MOESM1_ESM.pdf]

**Companionship at Hospital Discharge And Its Association With Subsequent Delirium Onset in Older Adults – The TRADE Observational Study.**

## Authors

Simone Brefka[1,2,3], Judith Adamo[4], Christoph Leinert[1,2,3], Johanna Braisch[5], Genia Decker[1], Rainer Muche[5], Thomas Seufferlein[6], Jochen Klaus[6], Lena Schulte-Kemna[6], Gerhard Eschweiler[7], Florian Gebhard[8], Konrad Schuetze[8], Tobias Geisler[9], Anke Bahrmann[10], Hugo A. Katus[10], Norbert Frey[10], Natascha-Elisabeth Denninger[11,12], Martin Mueller[11], Kathrin Pahmeier[13], Janine Biermann-Stallwitz[13], Juergen Wasem[13], Anna Lena Flagmeier[14], Petra Benzinger[15,16], Juergen Bauer[15], Michael Denkinger[1,2,3], Dhayana Dallmeier[3,17]

## Authors' institutional addresses

[1] Institute for Geriatric Research, University Hospital Ulm, Ulm, Germany
[2] Geriatric Center Ulm, Ulm, Germany
[3] Research Unit on Ageing, AGAPLESION Bethesda Hospital Ulm, Ulm, Germany
[4] Institute for Psychogerontology, Friedrich-Alexander University Erlangen-Nuremberg, Nuremberg, Germany
[5] Institute for Epidemiology and Medical Biometry, Ulm University, Ulm, Germany
[6] Department of Internal Medicine I, University Hospital Ulm, Ulm, Germany
[7] Geriatric Center at University Hospital Tuebingen, Tuebingen, Germany
[8] Department of Trauma-, Hand-, and Reconstructive Surgery, University Hospital Ulm, Ulm, Germany
[9] Department of Cardiology, University Hospital Tuebingen, Tuebingen, Germany
[10] Department of Cardiology, Angiology and Pneumology, University Hospital Heidelberg, Heidelberg, Germany
[11] Department for Primary Care and Health Services Research, Nursing Science and Interprofessional Care, Medical Faculty Heidelberg, Heidelberg University, Heidelberg, Germany
[12] Martin Luther University Halle-Wittenberg, International Graduate Academy, Institute for Health and Nursing Science, Medical Faculty, Halle (Saale), Germany
[13] Institute for Health Care Management and Research, University of Duisburg-Essen, Essen, Germany
[14] AOK – Allgemeine Ortskrankenkasse Baden-Wuerttemberg, Statutory Health Insurance Company, Stuttgart, Germany
[15] Geriatric Center, Medical Faculty Heidelberg, Heidelberg University, Heidelberg, Germany
[16] Institute of Health and Generations, Faculty of Social and Health Studies, University of Applied Sciences Kempten, Kempten, Germany
[17] Department of Epidemiology, Boston University School of Public Health, Boston, Massachusetts, USA

## Additional file 1 (supplementary material):

Table S1: STROBE checklist
Table S2: Characteristics of study population stratified by study centers (n=163)
Table S3: Characteristics of included vs. excluded participants (available vs. missing information on companionship during discharge/transfer)
Table S4: Characteristics of study population (n=163) stratified according to the type of CSC
Table S5: Selected and excluded variables for logistic regression
Table S6: Characteristics of study population stratified according to mode of transportation (car/taxi/bus/train/tram vs. patient/disabled transport ambulance) (n=146)
Table S7: I-CAM-S, FAM-CAM and Nu-DESC in the total study population (n=212)
Table S8: Delirium detection at T1 and T2 with different instruments
Table S9: 7-days delirium incidence proportion stratified by study centers
Table S10: Logistic regression evaluating the association between companionship and 7-days-delirium incidence

| **Table S1:** STROBE Statement for the TRADE observational study — Checklist of items that should be included in reports of cohort studies | | | |
|---|---|---|---|
| | **Item No** | **Recommendation** | **Page No** |
| **Title and abstract** | 1 | (*a*) Indicate the study's design with a commonly used term in the title or the abstract<br>(*b*) Provide in the abstract an informative and balanced summary of what was done and what was found | 1<br>3-4 |
| **Introduction** | | | |
| Background/ rationale | 2 | Explain the scientific background and rationale for the investigation being reported | 5-6 |
| Objectives | 3 | State specific objectives, including any prespecified hypotheses | 6-7 |
| **Methods** | | | |
| Study design | 4 | Present key elements of study design early in the paper | 7, 10-11 |
| Setting | 5 | Describe the setting, locations, and relevant dates, including periods of recruitment, exposure, follow-up, and data collection | 7-11 |
| Participants | 6 | (*a*) Give the eligibility criteria, and the sources and methods of selection of participants. Describe methods of follow-up<br>(*b*) For matched studies, give matching criteria and number of exposed and unexposed | 7-11<br>n.a. |
| Variables | 7 | Clearly define all outcomes, exposures, predictors, potential confounders, and effect modifiers. Give diagnostic criteria, if applicable | 11-14 |
| Data sources/ measurement | 8* | For each variable of interest, give sources of data and details of methods of assessment (measurement). Describe comparability of assessment methods if there is more than one group | 11-14 |
| Bias | 9 | Describe any efforts to address potential sources of bias | 14-15 |
| Study size | 10 | Explain how the study size was arrived at | 7-9 |
| Quantitative variables | 11 | Explain how quantitative variables were handled in the analyses. If applicable, describe which groupings were chosen and why | 11-14 |
| Statistical methods | 12 | (*a*) Describe all statistical methods, including those used to control for confounding<br>(*b*) Describe any methods used to examine subgroups and interactions<br>(*c*) Explain how missing data were addressed<br>(*d*) If applicable, explain how loss to follow-up was addressed<br>(*e*) Describe any sensitivity analyses | 14-15<br>14-15<br>8, 15<br>n.a.<br>n.a. |
| **Results** | | | |
| Participants | 13* | (a) Report numbers of individuals at each stage of study—eg numbers potentially eligible, examined for eligibility, confirmed eligible, included in the study, completing follow-up, and analysed<br>(b) Give reasons for non-participation at each stage<br>(c) Consider use of a flow diagram | 8-9<br>8-9<br>9 |
| Descriptive data | 14* | (a) Give characteristics of study participants (eg demographic, clinical, social) and information on exposures and potential confounders<br>(b) Indicate number of participants with missing data for each variable of interest<br>(c) Summarise follow-up time (eg, average and total amount) | 15-16, Table 1, Tables S2-S4, Table S6<br>n.a. |
| Outcome data | 15* | Report numbers of outcome events or summary measures over time | 16-20 |
| Main results | 16 | (*a*) Give unadjusted estimates and, if applicable, confounder-adjusted estimates and their precision (eg, 95% confidence interval). Make clear which confounders were adjusted for and why they were included<br>(*b*) Report category boundaries when continuous variables were categorized<br>(*c*) If relevant, consider translating estimates of relative risk into absolute risk for a meaningful time period | 17-20<br>n. a.<br>n. a. |

| | | | |
|---|---|---|---|
| Other analyses | 17 | Report other analyses done—eg analyses of subgroups and interactions, and sensitivity analyses | 18-19 |
| **Discussion** | | | |
| Key results | 18 | Summarise key results with reference to study objectives | 21-23 |
| Limitations | 19 | Discuss limitations of the study, taking into account sources of potential bias or imprecision. Discuss both direction and magnitude of any potential bias | 23-25 |
| Interpretation | 20 | Give a cautious overall interpretation of results considering objectives, limitations, multiplicity of analyses, results from similar studies, and other relevant evidence | 22-23, 25 |
| Generalisability | 21 | Discuss the generalisability (external validity) of the study results | 23-25 |
| **Other information** | | | |
| Funding | 22 | Give the source of funding and the role of the funders for the present study and, if applicable, for the original study on which the present article is based | 28 |
| *Give information separately for exposed and unexposed groups. | | | |

| **Table S2:** Characteristics of study population stratified by study centers | | | | | | | | | | |
|---|---|---|---|---|---|---|---|---|---|---|
| | **Total (N=163)** | **Number of missings** | **Heidelberg - Geriatrics (N=38)** | **Number of missings** | **Heidelberg – University Hospital (N=28)** | **Number of missings** | **Tuebingen (N=31)** | **Number of missings** | **Ulm (N=66)** | **Number of missings** |
| **Age (years)**, Median (Min, Q1, Q3, Max) | 80.8 (70.0,77.0,84.2,97.6) | 0 | 84.2 (72.8,80.4,89.4,96.6) | 0 | 79.7 (71.2,76.8,83.7,90.2) | 0 | 79.8 (70,76,82,88.4) | 0 | 80.8 (70.2,75,83.8,97.6) | 0 |
| **Women,** n (%) | 91 (55.8) | 0 | 23 (60.5) | 0 | 9 (32.1) | 0 | 21 (67.7) | 0 | 38 (57.6) | 0 |
| **School education,** n (%) | | | | | | | | | | |
| ≤ 10 years | 112 (68.7) | 0 | 22 (57.9) | 0 | 18 (64.3) | 0 | 20 (64.5) | 0 | 52 (78.8) | 0 |
| > 10 years | 51 (31.3) | | 16 (42.1) | | 10 (35.7) | | 11 (35.5) | | 14 (21.2) | |
| **Migration background,** n (%) | 20 (12.3) | 0 | 3 (7.9) | 0 | 2 (7.1) | 0 | 5 (16.1) | 0 | 10 (15.2) | 0 |
| **Systolic blood pressure (mmHg) on admission,** Median (Min, Q1, Q3, Max) | 130 (86,115,145,190) | 9 | 130 (86,118,149,180) | 1 | 130 (110,120,150,170) | 2 | 130 (100,115,140,190) | 0 | 130 (90,110,145,180) | 7 |
| **Diastolic blood pressure (mmHg) on admission,** Median (Min, Q1, Q3, Max) | 70 (40,65,80,109) | 9 | 70 (40,60,80,109) | 1 | 70 (60,70,80,90) | 2 | 75 (60,70,80,100) | 0 | 70 (45,65,80,95) | 7 |
| **Smoking status,** n (%) | | | | | | | | | | |
| non-smoker | 85 (52.2) | 0 | 20 (52.6) | 0 | 13 (46.4) | 0 | 13 (41.9) | 0 | 39 (59.1) | 0 |
| ex-smoker | 73 (44.8) | | 16 (42.1) | | 14 (50.0) | | 17 (54.8) | | 26 (39.4) | |
| current smoker | 5 (3.1) | | 2 (5.3) | | 1 (3.6) | | 1 (3.2) | | 1 (1.5) | |
| **Alcohol consumption,** n (%) | | | | | | | | | | |
| never | 39 (24.5) | 4 | 8 (22.9) | 3 | 5 (17.9) | 0 | 16 (53.3) | 1 | 10 (15.2) | 0 |
| formerly | 33 (20.8) | | 10 (28.6) | | 5 (17.9) | | 4 (13.3) | | 14 (21.2) | |
| currently | 87 (54.7) | | 17 (48.6) | | 18 (64.3) | | 10 (33.3) | | 42 (63.6) | |
| **Marital status,** n (%) | | | | | | | | | | |
| married/partnership | 88 (54.0) | 0 | 14 (36.8) | 0 | 22 (78.6) | 0 | 14 (45.2) | 0 | 38 (57.6) | 0 |
| single | 10 (6.1) | | 0 | | 1 (3.6) | | 3 (9.7) | | 6 (9.1) | |
| divorced/separated | 5 (3.1) | | 4 (10.5) | | 0 | | 0 | | 1 (1.5) | |
| widowed | 60 (36.8) | | 20 (52.6) | | 5 (17.9) | | 14 (45.2) | | 21 (31.8) | |
| **Children,** n (%) | 146 (89.6) | 0 | 37 (97.4) | 0 | 24 (85.7) | 0 | 29 (93.6) | 0 | 56 (84.9) | 0 |
| **Grandchildren,** n (%) | 119 (73.0) | 0 | 30 (79.0) | 0 | 19 (67.9) | 0 | 26 (83.9) | 0 | 44 (66.7) | 0 |
| **Current housing situation,** n (%) | | | | | | | | | | |
| own household | 153 (93.9) | 0 | 36 (94.7) | 0 | 28 (100.0) | 0 | 30 (96.8) | 0 | 59 (89.4) | 0 |
| household of children/ grandchildren/ other relatives | 1 (0.6) | | 0 | | 0 | | 0 | | 1 (1.5) | |
| assisted living | 9 (5.5) | | 2 (5.3) | | 0 | | 1 (3.2) | | 6 (9.1) | |
| Living alone, n (%) | 74 (45.4) | 0 | 23 (60.5) | 0 | 6 (21.4) | 0 | 17 (54.8) | 0 | 28 (42.4) | 0 |
| **Lubben** | | | | | | | | | | |

**Table S2:** Characteristics of study population stratified by study centers

| | Total (N=163) | Number of missings | Heidelberg - Geriatrics (N=38) | Number of missings | Heidelberg – University Hospital (N=28) | Number of missings | Tuebingen (N=31) | Number of missings | Ulm (N=66) | Number of missings |
|---|---|---|---|---|---|---|---|---|---|---|
| Median (Min, Q1, Q3, Max) | 16 (0,11,21,30) | | 13 (0,10,18,30) | | 16 (1,12,21,23) | | 16 (5,10,22,30) | | 17 (5,15,21,28) | |
| social isolation (< 12) , n (%) | 40 (26.1) | 10 | 16 (43.2) | 1 | 4 (16.0) | 3 | 9 (30.0) | 1 | 11 (18.0) | 5 |
| social support (≥ 12), n (%) | 113 (73.9) | | 21 (56.8) | | 21 (84.0) | | 21 (70.0) | | 50 (82.0) | |
| **Social contact with …,** n (%) | | | | | | | | | | |
| **Spouse** | 90 (55.2) | 0 | 14 (36.8) | 0 | 22 (78.6) | 0 | 14 (45.2) | 0 | 40 (60.6) | 0 |
| **Sister** | 69 (42.3) | 0 | 14 (36.8) | 0 | 12 (42.9) | 0 | 10 (32.3) | 0 | 33 (50.0) | 0 |
| **Brother** | 64 (39.5) | 1 | 14 (36.8) | 0 | 12 (44.4) | 1 | 10 (32.3) | 0 | 28 (42.4) | 0 |
| **Daughter** | 94 (57.7) | 0 | 22 (57.9) | 0 | 17 (60.7) | 0 | 18 (58.1) | 0 | 37 (56.1 | 0 |
| **Son** | 96 (58.9) | 0 | 26 (68.4) | 0 | 15 (53.6) | 0 | 19 (61.3) | 0 | 36 (54.6) | 0 |
| **Daughter-in-law** | 71 (43.6) | 0 | 21 (55.3) | 0 | 10 (35.7) | 0 | 17 (54.8) | 0 | 23 (34.9) | 0 |
| **Son-in-law** | 72 (44.2) | 0 | 15 (39.5) | 0 | 12 (42.9) | 0 | 15 (48.4) | 0 | 30 (45.5) | 0 |
| **Daughter and/or son-in-law** | | | | | | | | | | |
| one of both | 24 (14.7) | 0 | 9 (23.7) | 0 | 5 (17.9) | 0 | 3 (9.7) | 0 | 7 (10.6) | 0 |
| both | 71 (43.6) | | 14 (36.8) | | 12 (42.9) | | 15 (48.4) | | 30 (45.5) | |
| **Friend** | 141 (86.5) | 0 | 27 (71.1) | 0 | 26 (92.9) | 0 | 28 (90.3) | 0 | 60 (90.9) | 0 |
| **Neighbor** | 143 (89.4) | 3 | 29 (76.3) | 0 | 27 (96.4) | 0 | 24 (85.7) | 3 | 63 (95.5) | 0 |
| **Barthel Index**, Median (Min, Q1, Q3, Max) | 85 (0,57.5,100,100) | 11 | 70 (20,55,90,100) | 0 | 95 (45,60,100,100) | 1 | 97.5 (45,77.5,100,100) | 7 | 85 (0,55,100,100) | 3 |
| **Hearing problems,** n (%) | 81 (49.7) | 0 | 21 (55.3) | 0 | 14 (50.0) | 0 | 18 (58.1) | 0 | 28 (42.4) | 0 |
| **Vision problems,** n (%) | 94 (57.7) | 0 | 16 (42.1) | 0 | 14 (50.0) | 0 | 19 (61.3) | 0 | 45 (68.2) | 0 |
| **Fall during last 3 months,** n (%) | 79 (48.5) | 0 | 21 (55.3) | 0 | 8 (28.6) | 0 | 10 (32.3) | 0 | 40 (60.6) | 0 |
| **Walking aid,** n (%) | 85 (52.2) | 0 | 26 (68.4) | 0 | 11 (39.3) | 0 | 16 (51.6) | 0 | 32 (48.5) | 0 |
| **CSHA Clinical Frailty Scale** (1=very fit, 9=terminally ill), median (Min, Q1, Q3, Max) | 4 (1,3,6,8) | 2 | 6 (2,4,6,7) | 2 | 3 (1,2.5,4.5,7) | 0 | 4 (2,3,6,7) | 0 | 3.5 (1,3,5,8) | 0 |
| **Subjective general health,** n (%) | | | | | | | | | | |
| poor/fair | 83 (53.6) | 8 | 18 (56.3) | 6 | 16 (57.1) | 0 | 17 (54.8) | 0 | 32 (50.0) | 2 |
| good/very good/excellent | 72 (46.5) | | 14 (43.8) | | 12 (42.9) | | 14 (45.2) | | 32 (50.0) | |
| **Subjective mental health,** n (%) | | | | | | | | | | |
| poor/fair | 30 (19.5) | 9 | 8 (25.8) | 7 | 3 (10.7) | 0 | 4 (13.3) | 1 | 15 (23.1) | 1 |

| **Table S2:** Characteristics of study population stratified by study centers | | | | | | | | | | |
|---|---|---|---|---|---|---|---|---|---|---|
| | **Total (N=163)** | **Number of missings** | **Heidelberg - Geriatrics (N=38)** | **Number of missings** | **Heidelberg – University Hospital (N=28)** | **Number of missings** | **Tuebingen (N=31)** | **Number of missings** | **Ulm (N=66)** | **Number of missings** |
| good/very good/excellent | 124 (80.5) | | 23 (74.2) | | 25 (89.3) | | 26 (86.7) | | 50 (76.9) | |
| **Depression in PHQ-4,** n (%) | 41 (26.5) | 8 | 13 (37.1) | 3 | 5 (19.2) | 2 | 5 (16.1) | 0 | 18 (28.6) | 3 |
| **Anxiety in PHQ-4,** n (%) | 27 (17.5) | 9 | 8 (22.9) | 3 | 3 (11.5) | 2 | 7 (23.3) | 1 | 9 (14.3) | 3 |
| **Comorbidities** | | | | | | | | | | |
| **Cardiovascular diseases** (heart attack, coronary heart disease, valvular heart diseases, heart failure, cardiac arrhythmia, peripheral artery occlusive disease, other arterial disorders), n (%) | 120 (73.6) | 0 | 29 (76.3) | 0 | 28 (100) | 0 | 26 (83.9) | 0 | 37 (56.1) | 0 |
| **Hypertension,** n (%) | 119 (73.0) | 0 | 33 (86.8) | 0 | 21 (75.0) | 0 | 28 (90.3) | 0 | 37 (56.1) | 0 |
| **Diabetes mellitus,** n (%) | 46 (28.2) | 0 | 11 (29.0) | 0 | 7 (25.0) | 0 | 11 (35.5) | 0 | 17 (25.8) | 0 |
| **Chronic lung disease,** n (%) | 28 (17.2) | 0 | 9 (23.7) | 0 | 6 (21.4) | 0 | 8 (25.8) | 0 | 5 (7.6) | 0 |
| **Sleep apnea syndrome,** n (%) | 9 (5.5) | 0 | 0 | 0 | 1 (3.6) | 0 | 3 (9.7) | 0 | 5 (7.6) | 0 |
| **Malignant tumor disease,** n (%) | 42 (25.8) | 0 | 11 (29.0) | 0 | 6 (21.4) | 0 | 12 (38.7) | 0 | 13 (19.7) | 0 |
| **Depression,** n (%) | 36 (22.1) | 0 | 13 (34.2) | 0 | 2 (7.1) | 0 | 5 (16.1) | 0 | 16 (24.2) | 0 |
| **Stroke,** n (%) | 28 (17.2) | 0 | 9 (23.7) | 0 | 2 (7.1) | 0 | 8 (25.8) | 0 | 9 (13.6) | 0 |
| **Paralysis,** n (%) | 14 (8.6) | 0 | 4 (10.5) | 0 | 0 | 0 | 2 (6.5) | 0 | 8 (12.1) | 0 |
| **Traumatic brain injury,** n (%) | 15 (9.2) | 0 | 5 (13.2) | 0 | 1 (3.6) | 0 | 2 (6.5) | 0 | 7 (10.6) | 0 |
| **Dementia,** n (%) | 15 (9.2) | 0 | 6 (15.8) | 0 | 1 (3.6) | 0 | 3 (9.7) | 0 | 5 (7.6) | 0 |
| **Parkinson's disease,** n (%) | 4 (2.5) | 0 | 1 (2.6) | 0 | 1 (3.6) | 0 | 1 (3.2) | 0 | 1 (1.5) | 0 |
| **Cerebral hemorrhage,** n (%) | 6 (3.7) | 0 | 1 (2.6) | 0 | 0 | 0 | 2 (6.5) | 0 | 3 (4.6) | 0 |
| **CCI,** Median (Min, Q1, Q3, Max) | 2 (0,1,4,10) | 0 | 2.5 (0,1,5,8) | 0 | 1.5 (0,0.5,4,9) | 0 | 2 (0,1,4,7) | 0 | 1 (0,1,3,10) | 0 |
| **Relevant diseases** (poor circulation or diabetes mellitus or blood cancer or epileptic seizures), n (%) | 92 (56.4) | 0 | 21 (55.3) | 0 | 13 (46.4) | 0 | 24 (77.4) | 0 | 34 (51.5) | 0 |
| **Number of medicines,** Median (Min, Q1, Q3, Max) | 9 (2,7,12,20) | 0 | 10.5 (3,7,13,18) | 0 | 9 (4,6,12.5,19) | 0 | 10 (3,7,11,20) | 0 | 8.5 (2,6,11,18) | 0 |

| **Table S2:** Characteristics of study population stratified by study centers | | | | | | | | | | |
|---|---|---|---|---|---|---|---|---|---|---|
| | **Total (N=163)** | **Number of missings** | **Heidelberg - Geriatrics (N=38)** | **Number of missings** | **Heidelberg – University Hospital (N=28)** | **Number of missings** | **Tuebingen (N=31)** | **Number of missings** | **Ulm (N=66)** | **Number of missings** |
| **Polymedication (≥ 5 medicines),** n (%) | 152 (93.3) | 0 | 37 (97.4) | 0 | 27 (96.4) | 0 | 27 (87.1) | 0 | 61 (92.4) | 0 |
| **Cognition** | | | | | | | | | | |
| **MoCA** | | | | | | | | | | |
| Blind version, n (%) | 5 (3.1) | | 1 (2.6) | | 0 | | 1 (3.2) | 4 | 3 (4.5) | |
| Sum score (without blind), Median (Min, Q1, Q3, Max) | 22 (7,19,24,29) | 26 | 20 (7,17,24,26) | 7 | 22 (13,21,23,29) | 3 | 24 (16,21,25,29) | 3 | 21.5 (13,19,24,28) | 13 |
| Sum score (only blind), Median (Min, Q1, Q3, Max) | 17 (9,13,17,22) | 0 | 22 (22,22,22,22) | 0 | - | - | 17 (17,17,17,17) | 0 | 13 (9,9,17,17) | 0 |
| Conspicuously[1] (incl. blind), n (%) | 119 (86.9) | 26 | 29 (93.6) | 7 | 22 (88.0) | 3 | 23 (82.1) | 3 | 45 (84.9) | 13 |
| **Subjective memory impairment,** n(%) | | | | | | | | | | |
| yes | 96 (58.9) | 0 | 22 (57.9) | 0 | 18 (64.3) | 0 | 22 (71.0) | 0 | 34 (51.5) | 0 |
| no | 64 (39.3) | | 16 (42.1) | | 10 (35.7) | | 8 (25.8) | | 30 (45.5) | |
| unknown | 3 (1.8) | | 0 | | 0 | | 1 (3.2) | | 2 (3.0) | |
| **History of previous delirium,** n (%) | | | | | | | | | | |
| yes | 24 (14.7) | 0 | 10 (26.3) | 0 | 3 (10.7) | 0 | 4 (12.9) | 0 | 7 (10.6) | 0 |
| no | 135 (82.8) | | 28 (73.7) | | 24 (85.7) | | 25 (80.7) | | 58 (87.9) | |
| unknown | 4 (2.5) | | 0 | | 1 (3.6) | | 2 (6.5) | | 1 (1.5) | |
| **DRAS,** Median (Min, Q1, Q3, Max) | 7 (1,5,8,12) | 10 | 8 (4,7,8,12) | 0 | 7 (2,5,8,10) | 2 | 6 (2,5,8,9) | 5 | 6 (1,4,8,11) | 3 |
| **Discharge/transfer (T1)** | | | | | | | | | | |
| **Length of hospital stay (days)**, Median (Min, Q1, Q3, Max) | 8 (1,4,17,61) | 1 | 18.5 (2,16,22,61) | 0 | 7 (1,4.5,10,35) | 0 | 2.5 (1,2,6,25) | 1 | 8 (1,4,12,37) | 0 |
| **Discharge environment**, n (%) | | | | | | | | | | |
| **unknown** (been there < 6 months) | 53 (32.5) | 0 | 19 (50.0) | 0 | 2 (7.1) | 0 | 7 (22.6) | 0 | 25 (37.9) | 0 |
| of these n (%) never been there | 44 (83.0) | | 18 (94.7) | | 2 (100) | | 4 (57.1) | | 20 (80.0) | |
| **known** (been there ≥ 6 months) | 110 (67.5) | | 19 (50.0) | | 26 (92.9) | | 24 (77.4) | | 41 (62.1) | |
| of these n (%) home | 108 (98.2) | | 19 (100) | | 26 (100) | | 23 (95.8) | | 40 (97.6) | |

[1] Defined as sum < 26 (normal version) and sum < 18 (blind version)

| **Table S2:** Characteristics of study population stratified by study centers | | | | | | | | | | |
|---|---|---|---|---|---|---|---|---|---|---|
| | **Total (N=163)** | **Number of missings** | **Heidelberg - Geriatrics (N=38)** | **Number of missings** | **Heidelberg – University Hospital (N=28)** | **Number of missings** | **Tuebingen (N=31)** | **Number of missings** | **Ulm (N=66)** | **Number of missings** |
| Abbreviations: CSHA = Canadian Study on Health and Aging; PHQ-4 = Patient Health Questionnaire-4, CCI = Charlson Comorbidity Index, MoCA = Montreal Cognitive Assessment, DRAS = Delirium Risk Assessment Score | | | | | | | | | | |

| **Table S3:** Characteristics of included vs. excluded participants (available vs. missing information on companionship during discharge/transfer) | | | | | | |
|---|---|---|---|---|---|---|
| **Variable** | **Total (n=212)** | **Number of missings** | **Study population with information on companionship (n=163)** | **Number of missings** | **Study population without information on companionship (n=49)** | **Number of missings** |
| **Age (years)**, Median (Min, Q1, Q3, Max) | 80.8 (70.0,76.8,84.6,97.6) | 0 | 80.8 (70.0,77.0,84.2,97.6) | 0 | 80.6 (70.2,76.0,84.8,91.0) | 0 |
| **Women,** n (%) | 115 (54.3) | 0 | 91 (55.8) | 0 | 24 (49.0) | 0 |
| **School education,** n (%) | | 0 | | 0 | | 0 |
| ≤ 10 years | 148 (69.8) | | 112 (68.7) | | 36 (73.5) | |
| > 10 years | 64 (30.2) | | 51 (31.3) | | 13 (26.5) | |
| **Migration background,** n (%) | 27 (12.7) | 0 | 20 (12.3) | 0 | 7 (14.3) | 0 |
| **Center** | | 0 | | 0 | | 0 |
| Ulm University Hospital | 79 (37.3) | | 66 (40.5) | | 13 (26.5) | |
| Heidelberg Geriatrics | 51 (24.1) | | 38 (23.3) | | 13 (26.5) | |
| Heidelberg University Hospital | 46 (21.7) | | 28 (17.2) | | 18 (36.7) | |
| Tuebingen University Hospital | 36 (17.0) | | 31 (19.0) | | 5 (10.2) | |
| **Systolic blood pressure (mmHg) on admission,** Median (Min, Q1, Q3, Max) | 130 (86,115,145,190) | 26 | 130 (86,115,145,190) | 9 | 137.5 (100,112.5,149.5,185) | 17 |
| **Diastolic blood pressure (mmHg) on admission,** Median (Min, Q1, Q3, Max) | 70 (40,65,80,109) | 26 | 70 (40,65,80,109) | 9 | 72.5 (50,65,80,100) | 17 |
| **Smoking status,** n (%) | | 1 | | 0 | | 1 |
| non-smoker | 114 (54.0) | | 85 (52.2) | | 29 (60.4) | |
| ex-smoker | 89 (42.2) | | 73 (44.8) | | 16 (33.3) | |
| current smoker | 8 (3.8) | | 5 (3.1) | | 3 (6.3) | |
| **Alcohol consumption,** n (%) | | 7 | | 4 | | 3 |
| never | 47 (22.9) | | 39 (24.5) | | 8 (17.4) | |
| formerly | 47 (22.9) | | 33 (20.8) | | 14 (30.4) | |
| currently | 111 (54.2) | | 87 (54.7) | | 24 (52.2) | |
| **Marital status,** n (%) | | 0 | | 0 | | 0 |
| married/partnership | 116 (54.7) | | 88 (54.0) | | 28 (57.1) | |
| single | 16 (7.6) | | 10 (6.1) | | 6 (12.2) | |
| divorced/separated | 10 (4.7) | | 5 (3.1) | | 5 (10.2) | |
| widowed | 70 (33.0) | | 60 (36.8) | | 10 (20.4) | |
| **Children,** n (%) | 184 (86.8) | 0 | 146 (89.6) | 0 | 38 (77.6) | 0 |
| **Grandchildren,** n (%) | 152 (71.7) | 0 | 119 (73.0) | 0 | 33 (67.4) | 0 |
| **Current housing situation,** n (%) | | 0 | | 0 | | 0 |
| own household | 198 (93.4) | | 153 (93.9) | | 45 (91.8) | |
| household of children/ grandchildren/ other relatives | 1 (0.5) | | 1 (0.6) | | 0 (0.0) | |
| assisted living | 13 (6.1) | | 9 (5.5) | | 4 (8.2) | |
| Living alone, n (%) | 94 (44.3) | 0 | 74 (45.4) | 0 | 20 (40.8) | 0 |
| **Lubben** | | 12 | | 10 | | 2 |
| Median (Min, Q1, Q3, Max) | 16 (0,11,20,30) | | 16 (0,11,21,30) | | 14 (2,10,19,25) | |
| social isolation (< 12) , n (%) | 54 (27.0) | | 40 (26.1) | | 14 (29.8) | |
| social support (≥ 12), n (%) | 146 (73.0) | | 113 (73.9) | | 33 (70.2) | |
| **Social contact with …,** n (%) | | | | | | |
| Spouse | 120 (56.6) | 0 | 90 (55.2) | 0 | 30 (61.2) | 0 |
| Sister | 87 (41.0) | 0 | 69 (42.3) | 0 | 18 (36.7) | 0 |
| Brother | 81 (38.4) | 1 | 64 (39.5) | 1 | 17 (34.7) | 0 |
| Daughter | 126 (59.4) | 0 | 94 (57.7) | 0 | 32 (65.3) | 0 |

**Table S3:** Characteristics of included vs. excluded participants (available vs. missing information on companionship during discharge/transfer)

| Variable | Total (n=212) | Number of missings | Study population with information on companionship (n=163) | Number of missings | Study population without information on companionship (n=49) | Number of missings |
|---|---|---|---|---|---|---|
| Son | 121 (57.1) | 0 | 96 (58.9) | 0 | 25 (51.0) | 0 |
| Daughter-in-law | 86 (40.6) | 0 | 71 (43.6) | 0 | 15 (30.6) | 0 |
| Son-in-law | 98 (46.2) | 0 | 72 (44.2) | 0 | 26 (53.1) | 0 |
| Social contact: Daughter and/or son-in-law | | 0 | | 0 | | 0 |
| one of both | 32 (15.1) | | 24 (14.7) | | 8 (16.3) | |
| both | 96 (45.3) | | 71 (43.6) | | 25 (51.0) | |
| Friend | 184 (86.8) | 0 | 141 (86.5) | 0 | 43 (87.8) | 0 |
| Neighbor | 185 (88.9) | 4 | 143 (89.4) | 3 | 42 (87.5) | 1 |
| **Barthel Index**, Median (Min, Q1, Q3, Max) | 80 (0,55,100,100) | 14 | 85 (0,57.5,100,100) | 11 | 70 (35,50,90,100) | 3 |
| **Hearing problems,** n (%) | 108 (50.9) | 0 | 81 (49.7) | 0 | 27 (55.1) | 0 |
| **Vision problems,** n (%) | 127 (59.9) | 0 | 94 (57.7) | 0 | 33 (67.4) | 0 |
| **Fall during last 3 months,** n (%) | 106 (50.0) | 0 | 79 (48.5) | 0 | 27 (55.1) | 0 |
| **Walking aid,** n (%) | 119 (56.1) | 0 | 85 (52.2) | 0 | 34 (69.4) | 0 |
| **CSHA Clinical Frailty Scale** (1=very fit, 9=terminally ill), median (Min, Q1, Q3, Max) | 4 (1,3,6,8) | 2 | 4 (1,3,6,8) | 2 | 4 (1,3,6,8) | 0 |
| **Subjective general health,** n (%) | | 10 | | 8 | | 2 |
| poor/fair | 117 (57.9) | | 83 (53.6) | | 34 (72.3) | |
| good/very good/excellent | 85 (42.1) | | 72 (46.5) | | 13 (27.7) | |
| **Subjective mental health,** n (%) | | 11 | | 9 | | 2 |
| poor/fair | 40 (19.9) | | 30 (19.5) | | 10 (21.3) | |
| good/very good/excellent | 161 (80.1) | | 124 (80.5) | | 37 (78.7) | |
| **Depression in PHQ-4,** n (%) | 55 (27.2) | 10 | 41 (26.5) | 8 | 14 (29.8) | 2 |
| **Anxiety in PHQ-4,** n (%) | 36 (17.9) | 11 | 27 (17.5) | 9 | 9 (19.2) | 2 |
| **Comorbidities** | | | | | | |
| **Cardiovascular diseases** (heart attack, coronary heart disease, valvular heart diseases, heart failure, cardiac arrhythmia, peripheral artery occlusive disease, other arterial disorders), n (%) | 155 (73.1) | 0 | 120 (73.6) | 0 | 35 (71.4) | 0 |
| **Hypertension,** n (%) | 157 (74.1) | 0 | 119 (73.0) | 0 | 38 (77.6) | 0 |
| **Diabetes mellitus,** n (%) | 60 (28.3) | 0 | 46 (28.2) | 0 | 14 (28.6) | 0 |
| **Chronic lung disease,** n (%) | 39 (18.4) | 0 | 28 (17.2) | 0 | 11 (22.5) | 0 |
| **Sleep apnea syndrome,** n (%) | 11 (5.2) | 0 | 9 (5.5) | 0 | 2 (4.1) | 0 |
| **Malignant tumor disease,** n (%) | 55 (25.9) | 0 | 42 (25.8) | 0 | 13 (26.5) | 0 |
| **Depression,** n (%) | 45 (21.2) | 0 | 36 (22.1) | 0 | 9 (18.4) | 0 |
| **Stroke,** n (%) | 35 (16.5) | 0 | 28 (17.2) | 0 | 7 (14.3) | 0 |
| **Paralysis,** n (%) | 20 (9.4) | 0 | 14 (8.6) | 0 | 6 (12.2) | 0 |
| **Traumatic brain injury,** n (%) | 21 (9.9) | 0 | 15 (9.2) | 0 | 6 (12.2) | 0 |
| **Dementia,** n (%) | 18 (8.5) | 0 | 15 (9.2) | 0 | 3 (6.1) | 0 |
| **Parkinson's disease,** n (%) | 6 (2.8) | 0 | 4 (2.5) | 0 | 2 (4.1) | 0 |
| **Cerebral hemorrhage,** n (%) | 8 (3.8) | 0 | 6 (3.7) | 0 | 2 (4.1) | 0 |

**Table S3:** Characteristics of included vs. excluded participants (available vs. missing information on companionship during discharge/transfer)

| Variable | Total (n=212) | Number of missings | Study population with information on companionship (n=163) | Number of missings | Study population without information on companionship (n=49) | Number of missings |
|---|---|---|---|---|---|---|
| **CCI,** Median (Min, Q1, Q3, Max) | 2 (0,1,4,10) | 0 | 2 (0,1,4,10) | 0 | 2 (0,1,4,8) | 0 |
| **Relevant diseases** (poor circulation or diabetes mellitus or blood cancer or epileptic seizures), n (%) | 116 (54.7) | 0 | 92 (56.4) | 0 | 24 (49.0) | 0 |
| **Number of medicines,** Median (Min, Q1, Q3, Max) | 9.5 (2,7,12,20) | 2 | 9 (2,7,12,20) | 0 | 10 (3,7,12,20) | 2 |
| **Polymedication (≥ 5 medicines),** n (%) | 195 (92.9) | 2 | 152 (93.3) | 0 | 43 (91.5) | 2 |
| **Cognition** | | | | | | |
| **MoCA** | | | | | | |
| Blind version, n (%) | 8 (3.8) | | 5 (3.1) | | 3 (6.1) | |
| Sum score (without blind), Median (Min, Q1, Q3, Max) | 21 (7,19,24,29) | 43 | 22 (7,19,24,29) | 26 | 20 (12,18,22,27) | 17 |
| Sum score (only blind), Median (Min, Q1, Q3, Max) | 15 (9,13,17,22) | 2 | 17 (9,13,17,22) | 0 | 13 (13,13,13,13) | 2 |
| Conspicuously[1] (incl. blind), n (%) | 146 (87.4) | 45 | 119 (86.9) | 26 | 27 (90.0) | 19 |
| **Subjective memory impairment,** n(%) | | 0 | | 0 | | 0 |
| yes | 120 (56.6) | | 96 (58.9) | | 24 (49.0) | |
| no | 87 (41.0) | | 64 (39.3) | | 23 (46.9) | |
| unknown | 5 (2.4) | | 3 (1.8) | | 2 (4.1) | |
| **History of previous delirium,** n (%) | | 0 | | 0 | | 0 |
| yes | 30 (14.2) | | 24 (14.7) | | 6 (12.2) | |
| no | 176 (83.0) | | 135 (82.8) | | 41 (83.7) | |
| unknown | 6 (2.8) | | 4 (2.5) | | 2 (4.1) | |
| **DRAS**, Median (Min, Q1, Q3, Max) | 7 (1,5,8,12) | 12 | 7 (1,5,8,12) | 10 | 6 (3,5,7,11) | 2 |
| **Discharge/transfer (T1)** | | | | | | |
| **Length of hospital stay (days)**, Median (Min, Q1, Q3, Max) | 10 (1,5,17,61) | 1 | 8 (1,4,17,61) | 1 | 12 (3,8,19,47) | 0 |
| **Discharge environment**, n (%) | | 44 | | 0 | | 44 |
| **unknown** (been there < 6 months) | 57 (33.9) | | 53 (32.5) | | 4 (80.0) | |
| of these n (%) never been there | 48 (84.2) | | 44 (83.0) | | 4 (100.0) | |
| **known** (been there ≥ 6 months) | 111 (66.1) | | 110 (67.5) | | 1 (20.0) | |
| of these n (%) home | 109 (98.2) | | 108 (98.2) | | 1 (100.0) | |

[1] Defined as sum < 26 (normal version) and sum < 18 (blind version)
Abbreviations: CSHA = Canadian Study on Health and Aging; PHQ-4 = Patient Health Questionnaire-4, CCI = Charlson Comorbidity Index, MoCA = Montreal Cognitive Assessment, DRAS = Delirium Risk Assessment Score

| **Table S4:** Characteristics of study population (n=163) stratified according to the type of CSC | | | | | | | |
|---|---|---|---|---|---|---|---|
| **Variable** | **Total (n=163)** | **Number of Missings** | **Optimal CSC (n=61)** | **Number of Missings** | **No/not-optimal CSC (n=102)** | **Number of Missings** | **p-value** |
| **Age (years)**, Median (Min, Q1, Q3, Max) | 80.8 (70.0,77.0,84.2,97.6) | 0 | 80.8 (70.2,77.4,85.2,97.6) | 0 | 80.9 (70.0,76.6,84.0,96.6) | 0 | 0.468 |
| **Women,** n (%) | 91 (55.8) | 0 | 29 (47.5) | 0 | 62 (60.8) | 0 | **0.099** |
| **School education,** n (%) | | 0 | | 0 | | 0 | 0.467 |
| ≤ 10 years | 112 (68.7) | | 44 (72.1) | | 68 (66.7) | | |
| > 10 years | 51 (31.3) | | 17 (27.9) | | 34 (33.3) | | |
| **Migration background,** n (%) | 20 (12.3) | 0 | 8 (13.1) | 0 | 12 (11.8) | 0 | 0.799 |
| **Center** | | 0 | | 0 | | 0 | **0.024** |
| Ulm University Hospital | 66 (40.5) | | 25 (41.0) | | 41 (40.2) | | |
| Heidelberg Geriatrics | 38 (23.3) | | 16 (26.2) | | 22 (21.6) | | |
| Heidelberg University Hospital | 28 (17.2) | | 15 (24.6) | | 13 (12.8) | | |
| Tuebingen University Hospital | 31 (19.0) | | 5 (8.2) | | 26 (25.5) | | |
| **Systolic blood pressure (mmHg) on admission,** Median (Min, Q1, Q3, Max) | 130 (86,115,145,190) | 9 | 130 (86,120,145,170) | 4 | 130 (89,115,145,190) | 5 | 0.578 |
| **Diastolic blood pressure (mmHg) on admission,** Median (Min, Q1, Q3, Max) | 70 (40,65,80,109) | 9 | 70 (40,69,81,90) | 4 | 70 (40,65,80,109) | 5 | 0.536 |
| **Smoking status,** n (%) | | 0 | | 0 | | 0 | **0.074** |
| non-smoker | 85 (52.2) | | 34 (55.7) | | 51 (50.0) | | |
| ex-smoker | 73 (44.8) | | 23 (37.7) | | 50 (49.0) | | |
| current smoker | 5 (3.1) | | 4 (6.6) | | 1 (1.0) | | |
| **Alcohol consumption,** n (%) | | 4 | | 2 | | 2 | **0.049** |
| never | 39 (24.5) | | 13 (22.0) | | 26 (26.0) | | |
| formerly | 33 (20.8) | | 7 (11.9) | | 26 (26.0) | | |
| currently | 87 (54.7) | | 39 (66.1) | | 48 (48.0) | | |
| **Marital status,** n (%) | | 0 | | 0 | | 0 | **0.048** |
| married/partnership | 88 (54.0) | | 38 (62.3) | | 50 (49.0) | | |
| single | 10 (6.1) | | 0 (0.0) | | 10 (9.8) | | |
| divorced/separated | 5 (3.1) | | 1 (1.6) | | 4 (3.9) | | |
| widowed | 60 (36.8) | | 22 (36.1) | | 38 (37.3) | | |
| **Children,** n (%) | 146 (89.6) | 0 | 58 (95.1) | 0 | 88 (86.3) | 0 | **0.075** |
| **Grandchildren,** n (%) | 119 (73.0) | 0 | 45 (73.8) | 0 | 74 (72.6) | 0 | **0.087** |
| **Current housing situation,** n (%) | | 0 | | 0 | | 0 | 0.134 |
| own household | 153 (93.9) | | 55 (90.2) | | 98 (96.1) | | |
| household of children/ grandchildren/ other relatives | 1 (0.6) | | 0 (0.0) | | 1 (1.0) | | |
| assisted living | 9 (5.5) | | 6 (9.8) | | 3 (2.9) | | |
| Living alone, n (%) | 74 (45.4) | 0 | 21 (34.4) | 0 | 53 (52.0) | 0 | **0.030** |
| **Lubben** | | 10 | | 4 | | 6 | |
| Median (Min, Q1, Q3, Max) | 16 (0,11,21,30) | | 17 (5,12,19,26) | | 16 (0,11,21,30) | | 0.504 |
| social isolation (< 12) , n (%) | 40 (26.1) | | 14 (24.6) | | 26 (27.1) | | 0.731 |
| social support (≥ 12), n (%) | 113 (73.9) | | 43 (75.4) | | 70 (72.9) | | |
| **Social contact with …,** n (%) | | | | | | | |
| Spouse | 90 (55.2) | 0 | 39 (63.9) | 0 | 51 (50.0) | 0 | **0.083** |
| Sister | 69 (42.3) | 0 | 28 (45.9) | 0 | 41 (40.2) | 0 | 0.476 |
| Brother | 64 (39.5) | 1 | 27 (45.0) | 1 | 37 (36.3) | 0 | 0.273 |
| Daughter | 94 (57.7) | 0 | 42 (68.9) | 0 | 52 (51.0) | 0 | **0.025** |
| Son | 96 (58.9) | 0 | 33 (54.1) | 0 | 63 (61.8) | 0 | 0.336 |
| Daughter in law | 71 (43.6) | 0 | 26 (42.6) | 0 | 45 (44.1) | 0 | 0.852 |

| Table S4: Characteristics of study population (n=163) stratified according to the type of CSC | | | | | | | |
|---|---|---|---|---|---|---|---|
| **Variable** | **Total (n=163)** | **Number of Missings** | **Optimal CSC (n=61)** | **Number of Missings** | **No/not-optimal CSC (n=102)** | **Number of Missings** | **p-value** |
| Son-in-law | 72 (44.2) | 0 | 34 (55.7) | 0 | 38 (37.3) | 0 | **0.022** |
| Daughter and/or son-in-law | | 0 | | 0 | | 0 | **0.046** |
| one of both | 24 (14.7) | | 10 (16.4) | | 14 (13.7) | | |
| both | 71 (43.6) | | 33 (54.1) | | 38 (37.3) | | |
| Friend | | 0 | | 0 | | 0 | 0.559 |
| Neighbor | 143 (89.4) | 3 | 57 (95.0) | 1 | 86 (86.0) | 2 | **0.074** |
| **Barthel Index**, Median (Min, Q1, Q3, Max) | 85 (0,57.5,100,100) | 11 | 90 (0,60,100,100) | 2 | 80 (20,55,100,100) | 9 | 0.578 |
| **Hearing problems,** n (%) | 81 (49.7) | 0 | 30 (49.2) | 0 | 51 (50.0) | 0 | 0.919 |
| **Vision problems,** n (%) | 94 (57.7) | 0 | 36 (59.0) | 0 | 58 (56.9) | 0 | 0.788 |
| **Fall during last 3 months,** n (%) | 79 (48.5) | 0 | 28 (45.9) | 0 | 51 (50.0) | 0 | 0.612 |
| **Walking aid,** n (%) | 85 (52.2) | 0 | 27 (44.3) | 0 | 58 (56.9) | 0 | 0.119 |
| **CSHA Clinical Frailty Scale** (1=very fit, 9=terminally ill), median (Min, Q1, Q3, Max) | 4 (1,3,6,8) | 2 | 3 (1,3,5,7) | 0 | 4 (1,3,6,8) | 2 | 0.207 |
| **Subjective general health,** n (%) | | 8 | | 3 | | 5 | 0.309 |
| poor/fair | 83 (53.6) | | 28 (48.3) | | 55 (56.7) | | |
| good/very good/excellent | 72 (46.5) | | 30 (51.7) | | 42 (43.3) | | |
| **Subjective mental health,** n (%) | | 9 | | 4 | | 5 | 0.965 |
| poor/fair | 30 (19.5) | | 11 (19.3) | | 19 (19.6) | | |
| good/very good/excellent | 124 (80.5) | | 46 (80.7) | | 78 (80.4) | | |
| **Depression in PHQ-4,** n (%) | 41 (26.5) | 8 | 15 (26.3) | 4 | 26 (26.5) | 4 | 0.977 |
| **Anxiety in PHQ-4,** n (%) | 27 (17.5) | 9 | 6 (10.7) | 5 | 21 (21.4) | 4 | **0.093** |
| **Comorbidities** | | | | | | | |
| **Cardiovascular diseases** (heart attack, coronary heart disease, valvular heart diseases, heart failure, cardiac arrhythmia, peripheral artery occlusive disease, other arterial disorders), n (%) | 120 (73.6) | 0 | 44 (72.1) | 0 | 76 (74.5) | 0 | 0.739 |
| **Hypertension,** n (%) | 119 (73.0) | 0 | 42 (68.9) | 0 | 77 (75.5) | 0 | 0.356 |
| **Poor circulation,** n (%) | 50 (30.7) | 0 | 12 (19.7) | 0 | 38 (37.3) | 0 | **0.019** |
| **Diabetes mellitus,** n (%) | 46 (28.2) | 0 | 12 (19.7) | 0 | 34 (33.3) | 0 | **0.061** |
| **Chronic lung disease,** n (%) | 28 (17.2) | 0 | 9 (14.8) | 0 | 19 (18.6) | 0 | 0.526 |
| **Sleep apnea syndrome,** n (%) | 9 (5.5) | 0 | 2 (3.3) | 0 | 7 (6.9) | 0 | 0.486 |
| **Malignant tumor disease,** n (%) | 42 (25.8) | 0 | 20 (32.8) | 0 | 22 (21.6) | 0 | 0.113 |
| **Blood cancer** | 5 (3.1) | 0 | 4 (6.6) | 0 | 1 (1.0) | 0 | **0.066** |
| **Depression,** n (%) | 36 (22.1) | 0 | 13 (21.3) | 0 | 23 (22.6) | 0 | 0.854 |
| **Stroke,** n (%) | 28 (17.2) | 0 | 8 (13.1) | 0 | 20 (19.6) | 0 | 0.288 |
| **Paralysis,** n (%) | 14 (8.6) | 0 | 4 (6.6) | 0 | 10 (9.8) | 0 | 0.573 |
| **Traumatic brain injury,** n (%) | 15 (9.2) | 0 | 5 (8.2) | 0 | 10 (9.8) | 0 | 0.731 |
| **Dementia,** n (%) | 15 (9.2) | 0 | 7 (11.5) | 0 | 8 (7.8) | 0 | 0.438 |
| **Parkinson's disease,** n (%) | 4 (2.5) | 0 | 0 (0.0) | 0 | 4 (3.9) | 0 | 0.298 |
| **Cerebral hemorrhage,** n (%) | 6 (3.7) | 0 | 2 (3.3) | 0 | 4 (3.9) | 0 | 1.000 |
| **Epileptic seizures,** n (%) | 8 (4.9) | 0 | 6 (9.8) | 0 | 2 (2.0) | 0 | **0.053** |
| **CCI,** Median (Min, Q1, Q3, Max) | 2 (0,1,4,10) | 0 | 2 (0,1,4,9) | 0 | 2 (0,1,4,10) | 0 | 0.718 |

| **Table S4:** Characteristics of study population (n=163) stratified according to the type of CSC | | | | | | | |
|---|---|---|---|---|---|---|---|
| **Variable** | **Total (n=163)** | **Number of Missings** | **Optimal CSC (n=61)** | **Number of Missings** | **No/not-optimal CSC (n=102)** | **Number of Missings** | **p-value** |
| **Relevant diseases** (poor circulation or diabetes mellitus or blood cancer or epileptic seizures), n (%) | 92 (56.4) | 0 | 27 (44.3) | 0 | 65 (63.7) | 0 | **0.015** |
| **Number of relevant diseases** | | 0 | | 0 | | 0 | **0.033** |
| **1** | 77 (47.2) | | 22 (36.1) | | 55 (53.9) | | |
| **2** | 14 (8.6) | | 4 (6.6) | | 10 (9.8) | | |
| **3** | 0 (0.0) | | 0 (0.0) | | 0 (0.0) | | |
| **4** | 1 (0.6) | | 1 (1.6) | | 0 (0.0) | | |
| **Number of medicines,** Median (Min, Q1, Q3, Max) | 9 (2,7,12,20) | 0 | 8 (2,6,11,19) | 0 | 10 (3,7,12,20) | 0 | 0.213 |
| **Polymedication (≥ 5 medicines),** n (%) | 152 (93.3) | 0 | 57 (93.4) | 0 | 95 (93.1) | 0 | 0.940 |
| **Cognition** | | | | | | | |
| **MoCA** | | | | | | | |
| Blind version, n (%) | 5 (3.1) | | 3 (4.9) | | 2 (2.0) | | |
| Sum score (without blind), Median (Min, Q1, Q3, Max) | 22 (7,19,24,29) | 26 | 21 (7,19,24,28) | 8 | 22 (10,19,25,29) | 18 | 0.978 |
| Sum score (only blind), Median (Min, Q1, Q3, Max) | 17 (9,13,17,22) | 0 | 13 (9,9,17,17) | 0 | 19.5 (17,17,22,22) | 0 | 0.139 |
| Conspicuously[1] (incl. blind), n (%) | 119 (86.9) | 26 | 47 (88.7) | 8 | 72 (85.7) | 18 | 0.617 |
| **Subjective memory impairment,** n(%) | | 0 | | 0 | | 0 | 0.600 |
| yes | 96 (58.9) | | 33 (54.1) | | 63 (61.8) | | |
| no | 64 (39.3) | | 27 (44.3) | | 37 (36.3) | | |
| unknown | 3 (1.8) | | 1 (1.6) | | 2 (2.0) | | |
| **History of previous delirium,** n (%) | | 0 | | 0 | | 0 | 0.799 |
| yes | 24 (14.7) | | 8 (13.1) | | 16 (15.7) | | |
| no | 135 (82.8) | | 51 (83.6) | | 84 (82.4) | | |
| unknown | 4 (2.5) | | 2 (3.3) | | 2 (2.0) | | |
| **DRAS,** Median (Min, Q1, Q3, Max) | 7 (1,5,8,12) | 10 | 7 (1,6,8,11) | 3 | 7 (2,5,8,12) | 7 | **0.031** |
| **Discharge/transfer (T1)** | | | | | | | |
| **Length of hospital stay (days)**, Median (Min, Q1, Q3, Max) | 8 (1,4,17,61) | 1 | 10 (1,5,17,61) | 0 | 8 (1,3,17,35) | 1 | **0.064** |
| **Discharge environment**, n (%) | | 0 | | 0 | | 0 | **0.095** |
| **unknown** (been there < 6 months) | 53 (32.5) | | 15 (24.6) | | 38 (37.3) | | |
| of these n (%) never been there | 44 (83.0) | | 11 (73.3) | | 33 (86.8) | | |
| **known** (been there ≥ 6 months) | 110 (67.5) | | 46 (75.4) | | 64 (62.8) | | |
| of these n (%) home | 108 (98.2) | | 45 (97.8) | | 63 (98.4) | | |
| **Mode of transportation, n (%)** | | 4 | | 2 | | 2 | **0.005** |
| ambulance/patient transport | 54 (34.0) | | 12 (20.3) | | 42 (42.0) | | |
| car/taxi/bus/train/tram | 105 (66.0) | | 47 (79.7) | | 58 (58.0) | | |

[1] Defined as sum < 26 (normal version) and sum < 18 (blind version)

Abbreviations: CSHA = Canadian Study on Health and Aging; PHQ-4 = Patient Health Questionnaire-4, CCI = Charlson Comorbidity Index, MoCA = Montreal Cognitive Assessment, DRAS = Delirium Risk Assessment Score

| **Table S5:** Selected and excluded variables for logistic regression | |
|---|---|
| **Selected variables** | **Excluded variables** |
| Sex | |
| Center | |
| Smoking status | |
| Alcohol consumption | |
| Living alone | Marital status |
| | Social contact with spouse |
| Social contact with daughter and/or son-in-law | Children |
| | Grandchildren |
| | Social contact with daughter |
| | Social contact with son-in-law |
| Social contact with neighbor | |
| Anxiety in PHQ-4 | |
| Relevant diseases (poor circulation or diabetes mellitus or blood cancer or epileptic seizures) | Poor circulation |
| | Diabetes mellitus |
| | Blood cancer |
| | Epileptic seizures |
| | Number of relevant diseases |
| Length of hospital stay | |
| Mode of transportation | Discharge environment |

**Table S6:** Characteristics of study population stratified according to mode of transportation (car/taxi/bus/train/tram vs. patient/disabled transport ambulance) (n=146)

| Variable | Total (n=146) | Number of missings | Study population transported by car (n=95) | Number of missings | Study population transported by ambulance (n=51) | Number of missings |
|---|---|---|---|---|---|---|
| **Age (years)**, Median (Min, Q1, Q3, Max) | 80.7 (70.0,76.6,84.2,97.6) | 0 | 80.0 (70.0,75.2,83.2,94.6) | 0 | 82.4 (70.2,78.2,86.8,97.6) | 0 |
| **Women,** n (%) | 83 (56.9) | 0 | 51 (53.7) | 0 | 32 (62.8) | 0 |
| **School education,** n (%) | | 0 | | 0 | | 0 |
| ≤ 10 years | 100 (68.5) | | 69 (72.6) | | 31 (60.8) | |
| > 10 years | 46 (31.5) | | 26 (27.4) | | 20 (39.2) | |
| **Migration background,** n (%) | 17 (11.6) | 0 | 11 (11.6) | 0 | 6 (11.8) | 0 |
| **Center** | | 0 | | 0 | | 0 |
| Ulm University Hospital | 63 (43.2) | | 36 (37.9) | | 27 (52.9) | |
| Heidelberg Geriatrics | 31 (21.2) | | 14 (14.7) | | 17 (33.3) | |
| Heidelberg University Hospital | 26 (17.8) | | 24 (25.3) | | 2 (3.9) | |
| Tuebingen University Hospital | 26 (17.8) | | 21 (22.1) | | 5 (9.8) | |
| **Systolic blood pressure (mmHg) on admission,** Median (Min, Q1, Q3, Max) | 130 (86,115,145,190) | 9 | 130 (90,117.5,147,180) | 7 | 130 (86,115,145,190) | 2 |
| **Diastolic blood pressure (mmHg) on admission,** Median (Min, Q1, Q3, Max) | 70 (40,65,80,109) | 9 | 70 (40,70,80,100) | 7 | 70 (40,60,80,109) | 2 |
| **Smoking status,** n (%) | | 0 | | 0 | | 0 |
| non-smoker | 76 (52.1) | | 52 (54.7) | | 24 (47.1) | |
| ex-smoker | 67 (45.9) | | 41 (43.2) | | 26 (51.0) | |
| current smoker | 3 (2.1) | | 2 (2.1) | | 1 (2.0) | |
| **Alcohol consumption,** n (%) | | 0 | | 0 | | 0 |
| never | 36 (24.7) | | 26 (27.4) | | 10 (19.6) | |
| formerly | 30 (20.6) | | 14 (14.7) | | 16 (31.4) | |
| currently | 80 (54.8) | | 55 (57.9) | | 25 (49.0) | |
| **Marital status,** n (%) | | 0 | | 0 | | 0 |
| married/partnership | 78 (53.4) | | 55 (57.9) | | 23 (45.1) | |
| single | 10 (6.9) | | 5 (5.3) | | 5 (9.8) | |
| divorced/separated | 5 (3.4) | | 3 (3.2) | | 2 (3.9) | |
| widowed | 53 (36.3) | | 32 (33.7) | | 21 (41.2) | |
| **Children,** n (%) | 131 (89.7) | 0 | 86 (90.5) | 0 | 45 (88.2) | 0 |
| **Grandchildren,** n (%) | 106 (72.6) | 0 | 70 (73.7) | 0 | 36 (70.6) | 0 |
| **Current housing situation,** n (%) | | 0 | | 0 | | 0 |
| own household | 136 (93.2) | | 92 (96.8) | | 44 (86.3) | |
| household of children/ grandchildren/ other relatives | 1 (0.7) | | 0 | | 1 (2.0) | |
| assisted living | 9 (6.2) | | 3 (3.2) | | 6 (11.8) | |
| Living alone, n (%) | 68 (46.6) | 0 | 39 (41.1) | 0 | 29 (56.9) | 0 |
| **Lubben** | | 10 | | 7 | | 3 |
| Median (Min, Q1, Q3, Max) | 16 (0,11,20.5,30) | | 17 (5,12.5,21,30) | | 14.5 (0,9,19,28) | |
| social isolation (< 12) , n (%) | 36 (26.5) | | 17 (19.3) | | 19 (39.6) | |
| social support (≥ 12), n (%) | 100 (73.5) | | 71 (80.7) | | 29 (60.4) | |
| **Social contact with …,** n (%) | | | | | | |
| Spouse | 80 (54.8) | 0 | 56 (59.0) | 0 | 24 (47.1) | 0 |
| Sister | 61 (41.8) | 0 | 45 (47.4) | 0 | 16 (31.4) | 0 |
| Brother | 56 (38.6) | 1 | 32 (34.0) | 1 | 24 (47.1) | 0 |
| Daughter | 85 (58.2) | 0 | 57 (60.0) | 0 | 28 (54.9) | 0 |
| Son | 86 (58.9) | 0 | 54 (56.8) | 0 | 32 (62.8) | 0 |

| **Table S6:** Characteristics of study population stratified according to mode of transportation (car/taxi/bus/train/tram vs. patient/disabled transport ambulance) (n=146) | | | | | | |
|---|---|---|---|---|---|---|
| **Variable** | **Total (n=146)** | **Number of missings** | **Study population transported by car (n=95)** | **Number of missings** | **Study population transported by ambulance (n=51)** | **Number of missings** |
| Daughter-in-law | 63 (43.2) | 0 | 42 (44.2) | 0 | 21 (41.2) | 0 |
| Son-in-law | 64 (43.8) | 0 | 47 (49.5) | 0 | 17 (33.3) | 0 |
| Daughter and/or son-in-law | | 0 | | 0 | | 0 |
| one of both | 23 (15.8) | | 12 (12.6) | | 11 (21.6) | |
| both | 63 (43.2) | | 46 (48.4) | | 17 (33.3) | |
| Friend | 129 (88.4) | 0 | 87 (91.6) | 0 | 42 (82.4) | 0 |
| Neighbor | 131 (89.7) | 0 | 88 (92.6) | 0 | 43 (84.3) | 0 |
| **Barthel Index**, Median (Min, Q1, Q3, Max) | 85 (0,60,100,100) | 9 | 100 (25,80,100,100) | 6 | 55 (0,45,65,100) | 3 |
| **Hearing problems,** n (%) | 69 (47.3) | 0 | 48 (50.5) | 0 | 21 (41.2) | 0 |
| **Vision problems,** n (%) | 88 (60.3) | 0 | 62 (65.3) | 0 | 26 (51.0) | 0 |
| **Fall during last 3 months,** n (%) | 72 (49.3) | 0 | 36 (37.9) | 0 | 36 (70.6) | 0 |
| **Walking aid,** n (%) | 74 (50.7) | 0 | 35 (36.8) | 0 | 39 (76.5) | 0 |
| **CSHA Clinical Frailty Scale** (1=very fit, 9=terminally ill), median (Min, Q1, Q3, Max) | 4 (1,3,5,7) | 1 | 3 (1,3,4,7) | 0 | 5 (2,4,6,7) | 1 |
| **Subjective general health,** n (%) | | 3 | | 2 | | 1 |
| poor/fair | 77 (53.9) | | 47 (50.5) | | 30 (60.0) | |
| good/very good/excellent | 66 (46.2) | | 46 (49.5) | | 20 (40.0) | |
| **Subjective mental health,** n (%) | | 4 | | 3 | | 1 |
| poor/fair | 27 (19.0) | | 15 (16.3) | | 12 (24.0) | |
| good/very good/excellent | 115 (81.0) | | 77 (83.7) | | 38 (76.0) | |
| **Depression in PHQ-4,** n (%) | 38 (26.2) | 1 | 20 (21.1) | 0 | 18 (36.0) | 1 |
| **Anxiety in PHQ-4,** n (%) | 24 (16.4) | 0 | 13 (13.7) | 0 | 11 (21.6) | 0 |
| **Comorbidities** | | | | | | |
| **Cardiovascular diseases** (heart attack, coronary heart disease, valvular heart diseases, heart failure, cardiac arrhythmia, peripheral artery occlusive disease, other arterial disorders), n (%) | 106 (72.6) | 0 | 71 (74.7) | 0 | 35 (68.6) | 0 |
| **Hypertension,** n (%) | 105 (71.9) | 0 | 74 (77.9) | 0 | 31 (60.8) | 0 |
| **Diabetes mellitus,** n (%) | 39 (26.7) | 0 | 30 (31.6) | 0 | 9 (17.7) | 0 |
| **Chronic lung disease,** n (%) | 24 (16.4) | 0 | 14 (14.7) | 0 | 10 (19.6) | 0 |
| **Sleep apnea syndrome,** n (%) | 9 (6.2) | 0 | 5 (5.3) | 0 | 4 (7.8) | 0 |
| **Malignant tumor disease,** n (%) | 38 (26.0) | 0 | 25 (26.3) | 0 | 13 (25.5) | 0 |
| **Depression,** n (%) | 33 (22.6) | 0 | 15 (15.8) | 0 | 18 (35.3) | 0 |
| **Stroke,** n (%) | 25 (17.1) | 0 | 14 (14.7) | 0 | 11 (21.6) | 0 |
| **Paralysis,** n (%) | 9 (6.2) | 0 | 5 (5.3) | 0 | 4 (7.8) | 0 |
| **Traumatic brain injury,** n (%) | 12 (8.2) | 0 | 8 (8.4) | 0 | 4 (7.8) | 0 |
| **Dementia,** n (%) | 11 (7.5) | 0 | 6 (6.3) | 0 | 5 (9.8) | 0 |
| **Parkinson's disease,** n (%) | 4 (2.7) | 0 | 2 (2.1) | 0 | 2 (3.9) | 0 |
| **Cerebral hemorrhage,** n (%) | 6 (4.1) | 0 | 5 (5.3) | 0 | 1 (2.0) | 0 |
| **CCI,** Median (Min, Q1, Q3, Max) | 2 (0,1,4,10) | 0 | 2 (0,1,4,10) | 0 | 2 (0,1,4,8) | 0 |

| **Table S6:** Characteristics of study population stratified according to mode of transportation (car/taxi/bus/train/tram vs. patient/disabled transport ambulance) (n=146) | | | | | | |
|---|---|---|---|---|---|---|
| **Variable** | **Total (n=146)** | **Number of missings** | **Study population transported by car (n=95)** | **Number of missings** | **Study population transported by ambulance (n=51)** | **Number of missings** |
| **Relevant diseases** (poor circulation or diabetes mellitus or blood cancer or epileptic seizures), n (%) | 81 (55.5) | 0 | 51 (53.7) | 0 | 30 (58.8) | 0 |
| **Number of medicines,** Median (Min, Q1, Q3, Max) | 9 (2,6,12,20) | 0 | 9 (2,6,11,20) | 0 | 11 (5,7,13,18) | 0 |
| **Polymedication (≥ 5 medicines),** n (%) | 135 (92.5) | 0 | 84 (88.4) | 0 | 51 (100) | 0 |
| **Cognition** | | | | | | |
| **MoCA** | | | | | | |
| Blind version, n (%) | 4 (2.7) | | 3 (3.2) | | 1 (2.0) | |
| Sum score (<u>without</u> blind), Median (Min, Q1, Q3, Max) | 22 (10,19,24,29) | 22 | 22 (13,20,25,29) | 9 | 20 (10,17,24,29) | 13 |
| Sum score (<u>only</u> blind), Median (Min, Q1, Q3, Max) | 15 (9,11,19.5,22) | 0 | 13 (9,9,17,17) | 0 | 22 (22,22,22,22) | 0 |
| Conspicuously[1] (incl. blind), n (%) | 106 (85.5) | 22 | 72 (83.7) | 9 | 34 (89.5) | 13 |
| **Subjective memory impairment,** n(%) | | | | | | |
| yes | 84 (57.5) | 0 | 52 (54.7) | 0 | 32 (62.8) | 0 |
| no | 59 (40.4) | | 41 (43.2) | | 18 (35.3) | |
| unknown | 3 (2.1) | | 2 (2.1) | | 1 (2.0) | |
| **History of previous delirium,** n (%) | | | | | | |
| yes | 21 (14.4) | 0 | 10 (10.5) | 0 | 11 (21.6) | 0 |
| no | 122 (83.6) | | 82 (86.3) | | 40 (78.4) | |
| unknown | 3 (2.1) | | 3 (3.2) | | 0 | |
| **DRAS**, Median (Min, Q1, Q3, Max) | 7 (1,5,8,12) | 8 | 6 (1,5,8,12) | 6 | 7 (2,6,8,11) | 2 |
| **Discharge/transfer (T1)** | | | | | | |
| **Length of hospital stay (days)**, Median (Min, Q1, Q3, Max) | 8 (1,4,15,61) | 0 | 6 (1,3,11,26) | 0 | 15 (2,8,22,61) | 0 |
| **Discharge environment**, n (%) | | | | | | |
| **unknown** (been there < 6 months) | 46 (31.5) | | 4 (4.2) | | 42 (82.4) | |
| of these n (%) never been there | 39 (84.8) | 0 | 3 (75.0) | 0 | 36 (85.7) | 44 |
| **known** (been there ≥ 6 months) | 100 (68.5) | | 91 (95.8) | | 9 (17.7) | |
| of these n (%) home | 98 (98.0) | | 90 (98.9) | | 8 (88.9) | |

[1] Defined as sum < 26 (normal version) and sum < 18 (blind version)

Abbreviations: CSHA = Canadian Study on Health and Aging; PHQ-4 = Patient Health Questionnaire-4, CCI = Charlson Comorbidity Index, MoCA = Montreal Cognitive Assessment, DRAS = Delirium Risk Assessment Score

| **Table S7:** I-CAM-S, FAM-CAM and Nu-DESC in the total study population (n=212) | | | | |
|---|---|---|---|---|
| **instrument** | **available, n** | **positive, n (%)** | **missing, n** | **possibly missed delirium cases, n** |
| **I-CAM-S** | 175 | 5 (2.9) | 37 | 1 |
| **FAM-CAM** | 78 | 9 (11.5) | 134 | 15 |
| **Nu-DESC** | 60 | 6 (10) | 152 | 15 |

| **Table S8:** Delirium detection at T1 and T2 with different instruments | | | | | |
|---|---|---|---|---|---|
| **Delirium positive at timepoint** | **Delirium cases/n** | **I-CAM-S** | **FAM-CAM** | **Nu-DESC** | **FAM-CAM + Nu-DESC** |
| T1 only | 8 | 2 | 4 | 2 | |
| T2 only | 5 | 2 | 2 | 1 | |
| T1 + T2 | 2 | | 1 | | 1 |
| T1 (missing data at T2) | 1 | 1 | | | |
| T2 (missing data at T1) | 3 | | 1 | 2 | |

| **Table S9:** 7-days delirium incidence proportion stratified by study centers | | |
|---|---|---|
| **Study population with information on companionship and delirium (n=92)** | **Delirium cases/n** | **7-days delirium incidence proportion (%) [95 % CI]** |
| Ulm University Hospital | 6/30 | 20.0 [9.5; 37.3] |
| Heidelberg Geriatrics | 9/28 | 32.1 [17.9; 50.7] |
| Heidelberg University Hospital | 0/14 | 0.0 [0.0; 19.3] |
| Tuebingen University Hospital | 4/20 | 20.0 [8.1; 41.6] |

| **Table S10:** Logistic regression evaluating the association between companionship and 7-days delirium incidence | | | |
|---|---|---|---|
| | | **Model 1 unadjusted OR [95% CI]** | **Model 2 adjusted for the DRAS and center OR [95% CI]** |
| **Cross-sectoral companionship** | not-optimal | Ref. | Ref. |
| | optimal | 1.58 [0.56; 4.46] | 1.87 [0.60; 5.85] |
| **C-index** | | 0.553 | 0.684 |
