# Supplementary material for: Companionship at hospital discharge and its association with subsequent delirium onset in older adults – the TRADE observational study
Source: BMC Geriatr. 2026 Feb 21;26:370. doi: 10.1186/s12877-026-07194-3 (PMC12997767; doi:10.1186/s12877-026-07194-3)
Supplement: Supplementary file 2 — Supplementary Material 2: Additional file 2. Study questionnaire. [file 12877_2026_7194_MOESM2_ESM.pdf]

**Companionship at Hospital Discharge And Its Association With Subsequent Delirium Onset in Older Adults – The TRADE Observational Study.**


## Authors

Simone Brefka[1,2,3], Judith Brenneisen[4], Christoph Leinert[1,2,3], Johanna Braisch[5], Genia Decker[1], Rainer Muche[5], Thomas Seufferlein[6], Jochen Klaus[6], Lena Schulte-Kemna[6], Gerhard Eschweiler[7], Florian Gebhard[8], Konrad Schuetze[8], Tobias Geisler[9], Anke Bahrmann[10], Hugo A. Katus[10], Norbert Frey[10], Natascha-Elisabeth Denninger[11,12], Martin Müller[11], Kathrin Pahmeier[13], Janine Biermann-Stallwitz[13], Juergen Wasem[13], Anna Lena Flagmeier[14], Petra Benzinger[15,16], Juergen Bauer[15], Michael Denkinger[1,2,3], Dhayana Dallmeier[3,17]

## Authors' institutional addresses

1 Institute for Geriatric Research, University Hospital Ulm, Ulm, Germany
2 Geriatric Center Ulm, Ulm, Germany
3 Research Unit on Ageing, AGAPLESION Bethesda Hospital Ulm, Ulm, Germany
4 Institute for Psychogerontology, Friedrich-Alexander University Erlangen-Nuremberg, Nuremberg, Germany
5 Institute for Epidemiology und Medical Biometry, Ulm University, Ulm, Germany
6 Department of Internal Medicine I, University Hospital Ulm, Ulm, Germany
7 Geriatric Center at University Hospital Tuebingen, Tuebingen, Germany
8 Department of Trauma-, Hand-, and Reconstructive Surgery, University Hospital Ulm, Ulm, Germany
9 Department of Cardiology, University Hospital Tuebingen, Tuebingen, Germany
10 Department of Cardiology, Angiology and Pneumology, University Hospital Heidelberg, Heidelberg, Germany
11 Department for Primary Care and Health Services Research, Nursing Science and Interprofessional Care, Medical Faculty Heidelberg, Heidelberg University, Heidelberg, Germany
12 Martin Luther University Halle-Wittenberg, International Graduate Academy, Institute for Health and Nursing Science, Medical Faculty, Halle (Saale), Germany
13 Institute for Health Care Management and Research, University of Duisburg-Essen, Essen, Germany
14 AOK – Allgemeine Ortskrankenkasse Baden-Wuerttemberg, Statutory Health Insurance Company, Stuttgart, Germany
15 Center for Geriatric Medicine, AGAPLESION Bethanien Hospital Heidelberg, Heidelberg University Hospital, Heidelberg, Germany
16 Institute of Health and Generations, Faculty of Social and Health Studies, University of Applied Sciences Kempten, Kempten, Germany
17 Department of Epidemiology, Boston University School of Public Health, Boston, Massachusetts, USA


## Additional file 2 (supplementary material):

Study questionnaire

TRADE

# TRADE observational study

# Survey dates T0-T3

**Status: 28/03/2023**

| Form | | T0 | Discharge | T1 | T2 | T3 | Page |
|---|---|---|---|---|---|---|---|
| | Day of the visit | 0 | | 3 | 7 | 90 | |
| | at intervals after | Inclusion | | Discharge | Discharge | Discharge | |
| | Possible deviation in days | max. 4 days before discharge | | max. 1 earlier, max. 1 later | max. 1 earlier, max. 1 later | max. 7 earlier, max. 7 later | |
| **A.** | **Inclusion** | **before T0** | | | | | 4 |

| **Visiting plan** | | **T0** | **Discharge** | **T1** | **T2** | **T3** | |
|---|---|---|---|---|---|---|---|
| **1** | **General information about the visit** | **X** | **X** | **X** | **X** | **X** | 5 |
| **B** | **Questions for the study participant** | | | | | | 8 |
| **General information** | | | | | | | 8 |
| 2. | Socio-demographic baseline survey | **X** | | | | | 8 |
| 3. | Lubben-6 Social Network Scale | **X** | | | | | 10 |
| 4. | Mobility, hearing and vision | **X** | | | | | 11 |
| 5. | Visual acuity test | **X** | | | | | 12 |
| 6. | Subjective memory performance and delirium | **X** | | | | | 12 |
| 7. | Comorbidities | **X** | | | | | 13 |
| 8. | Consumption of addictive substances | **X** | | | | | 14 |
| 9. | Hand strength with dynamometer | **X** | | | | | 16 |
| Montreal Cognitive Assessment (MoCA) | | | | | | | 17 |
| 10. | Montreal Cognitive Assessment (MoCA) - initial examination | **X** | | | | | 17 |
| 11. | Montreal Cognitive Assessment (MoCA) - second examination | | | **X** | | | 17 |
| 12. | Montreal Cognitive Assessment (MoCA) - third examination | | | | | **until 16.03.20 X** | 17 |
| 13. | Telephone Montreal Cognitive Assessment (T-MoCA) | | | | | **from 16.03.20 X** | 18 |
| 14. | ICD-10 adapted Confusion Assessment Method Severity Score (I-CAM-S) | **X** | | **X** | **X** | **X** | 18 |
| 15. | Rivermead Mobility Index | **X** | | **X** | **X** | **X** | 18 |
| 16. | Patient Health Questionnaire 4 (PHQ-4) | **X** | | | | | 19 |
| 17. | Awareness of Age Related Change (AARC) | **X** | | | | | 19 |
| 18. | General Self-Efficacy Short Scale (Allgemeine Selbstwirksamkeit Kurzskala, ASKU) | **X** | | | | | 19 |
| 19. | Simplified nutritional appetite questionnaire (SNAQ) | **X** | | | **X** | **X** | 19 |
| 20. | Pittsburgh Sleep Quality Index (PSQI Basic) | **X** | | | | | 20 |
| Follow-up questionnaire - participant | | | | | | | 20 |
| 21. | Follow-up questions participant - discharge and new place of residence | | | **X** | | | 24 |
| 22. | Follow-up questions participant – health status | | | **X** | **X** | **X** | 24 |

TRADE

Project: TRADE observational study Date ___.___.______

Timepoint: O T0 O T1 O T2 O T3 Interviewer code number: _____

Centre: _______ Participant ID: _____

| | | T0 | Dismissal | T1 | T2 | T3 | Page |
|---|---|---|---|---|---|---|---|
| **C.** | **Questions for the caregiver** | | | | | | 27 |
| 23. | Instrumental activities of daily living according to Lawton (IADL) | X | | | | X | 27 |
| 24. | Follow-up questions caregiver - discharge and new place of residence | | | X | | | 28 |
| 25. | Follow-up questions caregiver - health status | | | X | X | X | 32 |
| 26. | Family CAM (FAM-CAM) | | | X | X | X | 34 |
| 27. | Informant Questionnaire on COgnitive Decline in the Elderly (IQCODE) | | | X | | | 34 |
| **D.** | **Data - Nurse/Medical file** | | | | | | 35 |
| 28. | Nursing Delirium Screening Scale (Nu-DESC) | X | | X | X | X | 35 |
| 29. | Barthel Index (according to the Hamburg Manual) | X | | | | X | 35 |
| 30. | Frailty scale (CSHA Clinical Frailty Scale) | X | | | | | 35 |
| 31. | Follow-up questions for nurses - new location | | | X | | | 36 |
| 32. | Follow-up questions for nurses – health status | | | X | X | X | 37 |
| 33. | Vital signs | X | | | | | 38 |
| 34. | Blood values | X | | | | | 39 |
| 35. | Medication list | X | | X | X | X | 41 |
| 36. | Diagnoses | X | | | | | 42 |
| **E.** | **Data - Interviewer** | | | | | | 43 |
| 37. | Interviewer's assessment | X | | | | | 43 |
| **F.** | **Drop-out form** | **in the case of drop-out** | | | | | **44** |
| **G.** | **Adverse events** | **if required** | | | | | **45** |

TRADE

Project: TRADE observational study | Date ___.___.______

Timepoint: O T0 O T1 O T2 O T3 | Interviewer code number: _____

Centre: _______ | Participant ID: _____

# A. Inclusion [before start of study/T0]

## Review of the inclusion and exclusion criteria

| | Yes | No |
|---|---|---|
| **1. Please confirm: The patient is not in the immediate process of dying and is expected to survive the next 3 months according to the interviewer's and study doctor's evaluation?** | O | O |
| **2. Please confirm: The patient has sufficient German language skills (no clear language barrier)** | O | O |
| **3. Is the patient capable of giving consent?**<br>If no, is there a written declaration of consent from the authorised representative or carer? | O<br>O | O<br>O |
| **4. Is the patient at least 70 years old?** | O | O |
| **5. Is the patient being treated as an inpatient?** | O | O |
| **6. Is a transfer/discharge planned in the next few days?**<br>If yes, where to?<br>**O New discharge environment**<br>**O Old discharge environment** | O | O |
| **7. Are there clinical/anamnestic/external anamnestic indications that the patient is or was cognitively impaired?** | O | O |
| **8. Is there a written declaration of consent from a caregiver?** | O | O |
| ***Attention! The person to be informed must indicate the date (DD.MM.YYYY, e.g. 01.07.2019) and their name and render their signature by their own hand. Otherwise the consent is invalid and the person may not participate in the study!*** | | |
| ***Please include the patient and, if applicable, authorised representative and caregiver in the "Study participant identification list"*** *(available for download on the homepage)* | | |
| **9. Has the patient and, if applicable, the authorised representative and caregiver been included in the "Study participant identification list", including name, address, telephone, patient ID, etc.?** | O | O |
| **10. Is the patient insured with AOK Baden-Württemberg?**<br>If yes, is the declaration of consent for the transmission of routine data available?<br>**Health insurance number:** (10 digits with capital letters in the first position, followed by 9 digits)<br>___ ___ ___ ___ ___ ___ ___ ___ ___ ___ ___ | O<br>O | O<br>O |

## 1. General information [T0, T1, T2, T3]

[T0] <u>Only at timepoint T0</u>
**Has the inclusion form been completed in full**
O Yes O No => Attention: If the inclusion form is not completed in full, all forms will be blocked.

[T1, T2, T3] **Can the interview take place?**
O Yes, personal interview with the participant
O Yes, telephone interview with the participant
O No

[T1, T2, T3] **If interview is conducted by telephone or not at all, please state the reason**
O Telephone interview necessary (coronavirus situation)
O Participant has moved too far away (> 50 km)
O Participant refuses to be interviewed
O Participant is not able to be interviewed
O Appointment could not be arranged for this period
O Participant cannot be reached (please document the 5 contact attempts)
O Participant deceased
O Other/comment: _________________

[T3] ***T3 - Important notes for telephone interviews:***
**- For both the MoCA and the CAM-S, parts that cannot be collected by telephone are missing in this version (the images in the MoCA, the questions on acute/fluctuating course and disturbance of consciousness in the CAM-S).**
**- Please inform the participants at the beginning that the telephone interview will take about 30 minutes.**
**- Before the interview begins, ask the participants to insert any hearing aids they may have (and to switch them on).**
**- Tell the participant the name of the hospital and the city from which you are calling (even if calling from the "home office", state the name and city of the hospital). Remind them that they were at this hospital and that the first interviews also took place there.**

O Ulm - Gastroenterology
O Ulm - Trauma Surgery
O Heidelberg - Geriatrics
O Heidelberg - Cardiology, Angiology and Pneumology
O Tuebingen - Gastroenterology
O Tuebingen - Cardiology
O Tuebingen - Geriatrics
O Other centre/department: _________________

**Interviewer**
O 1 O 2 O 3 O 4 O 5 O 6 O 7 O 8 O 9 O 10 O Other: _______________

**Date of the survey**

| | Start date | Start time | End time |
|---|---|---|---|
| Elevation | _._.__ | _:_ | _:_ |
| [T0] possibly 2nd survey | _._.__ | _:_ | _:_ |
| [T0] 2nd survey - from assessment tool/questionnaire: | | ________________ * | |
| [T0] Other/Comment: | _________________ | | |

*

- Socio-demographic baseline survey
- Mobility, hearing and vision
- Visual acuity test
- Subjective memory performance and delirium
- Comorbidities
- Consumption of addictive substances

- Hand strength with dynamometer
- Montreal Cognitive Assessment (MoCA) - initial examination
- Confusion Assessment Method Severity Score (CAM-S)
- Charité Mobility Index (CHARMI)
- Rivermead Mobility Index
- Patient Health Questionnaire 4 (PHQ-4)
- Lubben-6 Social Network Scale
- Awareness of Age Related Change (AARC)
- General Self-Efficacy Short Scale (ASKU)
- Simplified nutritional appetite questionnaire (SNAQ)
- Pittsburgh Sleep Quality Index (PSQI Basic)

**Place of the survey**
O Acute care hospital
O Daycare
O At home
O Nursing home
O Geriatric rehabilitation
O Other location or other rehabilitation: _____________

**Conditions during the survey**

**Exact location**
O Patient room
O Separate examination room
O Ward/corridor
O Other/comment: ________

**Disturbances**
O No disturbances
O Slight disturbances (e.g. room neighbour talks quietly during examination)
O Severe interference (e.g. constant background noise)
O Other/comment: ________

**Other persons**
O No other persons
O Caregiver
O Room neighbour
O Hospital staff
O Other/comment: ___________

**[Discharge]**

***Note: after T0 and before T1***

***=> If discharge is postponed by more than 4 days after the start of the T0 survey, the study is cancelled***

**Was the date of discharge from hospital postponed by more than 4 days after the start of the T0 survey?**
O Yes => drop-out
O No
***Please complete the drop-out form in the case of drop-out***

**[T1] How far is the current location from the hospital where T0 was performed?**
(Record the distance in kilometres via Google Maps or similar)
_______ Kilometres

**[T1, T2]** ***Attention: If the interview with the caregiver on iADL has not yet been carried out, please carry it out here.***

**[T2]** ***Attention: If the second MoCA examination was not performed at T1, please perform it here.***

**[T1, T2, T3]** ***Attention: If the interview with the caregiver (follow-up questions on discharge and new place of residence / IQCODE) has not yet been conducted, please conduct it here.***

**[T1, T2, T3] If a caregiver is available and could not be reached for the interview, please indicate the 5 contact attempts:**

| Date and time | Type | Comment |
|---|---|---|
| 1. ___.___.______ ___:___ | O Mobile phone O Landline O Mail | O Other/comment: _______________ |
| 2. ___.___.______ ___:___ | O Mobile phone O Landline O Mail | O Other/comment: _______________ |
| 3. ___.___.______ ___:___ | O Mobile phone O Landline O Mail | O Other/comment: _______________ |
| 4. ___.___.______ ___:___ | O Mobile phone O Landline O Mail | O Other/comment: _______________ |
| 5. ___.___.______ ___:___ | O Mobile phone O Landline O Mail | O Other/comment: _______________ |

**[T3, as of 16 March 2020] If a participant could not be reached for the interview, please indicate the 5 contact attempts:**

| Date and time | Type | Comment |
|---|---|---|
| 1. ___.___.______ ___:___ | O Mobile phone O Landline O Mail | O Other/comment: _______________ |
| 2. ___.___.______ ___:___ | O Mobile phone O Landline O Mail | O Other/comment: _______________ |
| 3. ___.___.______ ___:___ | O Mobile phone O Landline O Mail | O Other/comment: _______________ |
| 4. ___.___.______ ___:___ | O Mobile phone O Landline O Mail | O Other/comment: _______________ |
| 5. ___.___.______ ___:___ | O Mobile phone O Landline O Mail | O Other/comment: _______________ |

# B. Questions for the STUDY PARTICIPANT

## 2. Socio-demographic baseline survey [T0]

1. **What is your current marital status?**
O Married/partnered
O Single
O Divorced or separated
O Widowed

2. **Do you have children (incl. adopted/step/foster children)**
O Yes O No
**If yes, how many?**
___ son/sons and ___ daughter/daughters

3. **Do you have grandchildren?**
O Yes O No
**If yes, how many?**
___ grandson/grandsons and ___ granddaughter/granddaughters

4. **What is your current living situation?**
O Own household
O Household of children/grandchildren/other relatives or family members
O Shared flat
O Nursing home/retirement home
O Multi-generation house
O Assisted living
O Homeless

5. **Who do you live with? (multiple answers possible)**
O Alone
O Spouse; partner
O Child(ren) **=> If with child(ren), number:** ___ child(ren)
O Grandchild(ren) **=> If with grandchild(ren), number:** ___ grandchild(ren)

O Other family members
O Shared flat
O Carer lives in the household

6. **What is your mother tongue?**
O German
O English
O French
O Italian
O Spanish
O Greek
O Croatian
O Russian
O Polish
O Turkish
O Other: ________________

7. **Did you, your parents or your grandparents grow up in a country other than Germany?**
O Yes O No
**If yes, who?**
O Self O Parents' generation O Grandparents' generation

**8. What is your highest general school-leaving qualification?**
O No school-leaving certificate
O Left school without a secondary school-leaving certificate (Volksschulabschluss) - maximum 8 years
O Completion of polytechnic secondary school 10th grade or before 1965: 8th grade - 8 years
O Secondary school-leaving certificate (Volksschulabschluss) - 9 years
O Secondary school-leaving certificate (intermediate school leaving certificate) - 10 years
O Entrance qualification for universities of applied sciences, completion of specialised secondary school - 12 years
O General or subject-specific higher education entrance qualification/Abitur (Gymnasium or EOS, also EOS with apprenticeship) - 13 years
O Other school-leaving qualification: ______________

**Number of school years:** Actual number, regardless of school-leaving qualification (repeated school years are not counted more than once)
___ school years

**9. Do you have vocational training?**
O Yes O No
***If yes, which one?***
O Apprenticeship (e.g. cook etc.) or school education (e.g. physiotherapy school etc.)
O (FH) degree programme
**Duration of apprenticeship/school education or (FH) degree programme in years**
___ years

**10. What was your main occupation?**
______________
**Duration of professional activity (total, also for different professions):**
___ years
**Age at retirement:**
___ years

**11. Do you have a care level?**
O Yes O No
**If yes, which one?**
O Care level 1 O Care level 2 O Care level 3 O Care level 4 O Care level 5
**If no, is an application for care grading currently being submitted?**
O Yes O No

**12. Do you have a living will?**
O Yes O No
**If yes, where do you keep it?** ___________________

**13. Do you have a health care proxy?**
O Yes O No
**If yes, where do you keep it?** ___________________

**14. The following questions ask about your social contacts: With whom and how often do you socialise in your familiar surroundings?**

| | Not available | Daily | Several times a week | Once a week | Several times a month | Once a month | Rarer |
|---|---|---|---|---|---|---|---|
| Partner | O | O | O | O | O | O | O |
| Sister | O | O | O | O | O | O | O |
| Brother | O | O | O | O | O | O | O |
| Daughter | O | O | O | O | O | O | O |
| Son | O | O | O | O | O | O | O |
| Daughter-in-law | O | O | O | O | O | O | O |
| Son-in-law | O | O | O | O | O | O | O |
| Granddaughter | O | O | O | O | O | O | O |
| Grandson | O | O | O | O | O | O | O |
| Niece | O | O | O | O | O | O | O |
| Nephew | O | O | O | O | O | O | O |
| Friend | O | O | O | O | O | O | O |
| Neighbour | O | O | O | O | O | O | O |
| Other | O | O | O | O | O | O | O |

If other or more, please specify person(s) and frequency:
______________________________________________________
______________________________________________________

**Questions were answered by/collected from:**
O Participant
O Partially participant and authorised representative or caregiver
O Only authorised representative or caregiver
O Partially participant and information from medical file
O Partially authorised representative or caregiver and details from medical file
O Information from medical file

## 3. Lubben-6 Social Network Scale [T0]

**Status**
O Questionnaire completely answered
O Questionnaire partially answered or survey cancelled
O Participant refuses to answer the questions
O Questions could not be answered
O Other/comment: ________________

***Details on the Lubben-6 Social Network Scale, and the questions and instructions can be found in references (1), (2) and (3).***

## 4. Mobility, hearing and vision [T0]

### Mobility

1. **Have you fallen in the last 3 months?**
   Note: A fall is defined as unintentionally coming to ground (on the floor, on a low level, the corridor, etc.).
   O Yes O No
   ***If yes, how often?***
   ___ times O Don't know (no answer)

2. **Have you almost fallen in the last 3 months?**
   O Yes O No
   ***If yes, how often?***
   ___ times O Don't know (no answer)

3. **Do you use a walking aid?**
   O Yes O No
   ***If yes, which one***
   O Walking stick O Crutches O Rollator/walking frame O Wheelchair O Other/comment: ________________

### Hearing

4. **Do you have hearing problems?**
   O Yes O No
   ***If yes, how pronounced would you say your problem is***
   Please rate on a scale from 0 to 10: 0= hardly any hearing problems, 10= very severe hearing problems
   Note: Show visual scale no. 1
   O 0 O 1 O 2 O 3 O 4 O 5 O 6 O 7 O 8 O 9 O 10

5. **Do you use a hearing aid?**
   O Yes O No

### Vision

6. **Do you have problems with your vision?**
   O Yes O No
   ***If yes, how pronounced would you say your problem is***
   Please rate on a scale from 0 to 10: 0= hardly any visual problems, 10= very severe visual problems
   Note: Show visual scale no. 1
   O 0 O 1 O 2 O 3 O 4 O 5 O 6 O 7 O 8 O 9 O 10

7. **Do you use a visual aid?**
   O Yes O No

8. **Do you have problems reading the headlines of the daily newspaper**
   O Yes O No

**Questions were answered by/collected from:**
O Participant
O Partially participant and authorised representative or caregiver
O Only authorised representative or caregiver
O Partially participant and information from medical file
O Partially authorised representative or caregiver and information from medical file
O Information from medical file

## 5. Visual acuity test [T0]

**Status**
O Visual acuity test performed
O Visual acuity test cancelled
O Participant refuses visual acuity test
O Visual acuity test not possible for participant
O Other/comment: ________________

1. **Does the participant use a visual aid?**
   O Yes O No

2. ***"I now ask you to read out the third line."***
   Is it possible to correctly recognise or read the entire line?
   O Yes O No

3. ***"I now have another question about the development of your eyesight. Has your vision deteriorated recently?"***
   O Yes O No

## 6. Subjective memory performance and delirium [T0]

1. **Do you have the feeling that your memory is getting worse?**
   O Yes O No O Don't know (no answer)

   **If yes, does that worry you?**
   O Yes, that worries me
   O Yes, that worries me a lot
   O No
   O Don't know (no answer)

2. **Have you already experienced delirium (an acute state of confusion, e.g. after an operation)?**
   O Yes O No O Don't know (no answer)

   **If yes, how often?**
   ___ times

   **Last in the year:**
   _____ (YYYY)

   **Brief description of the incident:**
   ________________________________________________________
   ________________________________________________________
   ________________________________________________________

**Questions were answered by/collected from:**
O Participant
O Partially participant and authorised representative or caregiver
O Only authorised representative or caregiver
O Partially participant and information from medical file
O Partially authorised representative or caregiver and information from medical file
O Information from medical file

## 7. Comorbidities [T0]

***In the case of severe cognitive impairment, the questions must be completed with the support of the authorised representative or caregiver.***

**Questioning the participant about previous illnesses that are particularly relevant to delirium:**
Please first enter the comorbidities from the medical file and then compare them with the study participant.
***"I will now ask you about the number of illnesses you have had. It is only an illness if the symptoms have lasted for at least 3 months or if you are being treated for the illness or if a doctor has been treating you for a longer period of time."***
***"Has a doctor ever told you that you have …?"*** (name the respective illness)

| | Yes | No | White Not |
|---|---|---|---|
| **- Eye diseases with severe visual impairment** | O | O | O |
| **- Circulatory weakness with fainting, light-headedness, blackness before the eyes or circulatory collapse (incl. (pre-)syncope, orthostatic hypotension)** | O | O | O |
| **- Stroke (incl. cerebral ischaemia, apoplexy, transient ischaemic attack (TIA))** | O | O | O |
| **- Brain haemorrhage** | O | O | O |
| **- Craniocerebral trauma (incl. cerebral concussion, skull contusion, head laceration)** | O | O | O |
| **- Inflammation of the brain and meninges (incl. meningitis, encephalitis)** | O | O | O |
| **- Persistent signs of paralysis** | O | O | O |
| **- Epileptic seizures (NOT: (convulsive) syncope)** | O | O | O |
| **- Parkinson's disease** | O | O | O |
| **- Dementia** | O | O | O |
| **- Mood disorders and depression** | O | O | O |
| **- Other mental illnesses (incl. schizophrenia, hallucinations, delusions)** | O | O | O |
| **- Tube and catheter systems** | O | O | O |
| **- Miscellaneous** | O | O | O |

If other, please specify

____________________________________________________________

**Questions were answered by/collected from:**
O Participant
O Partially participant and authorised representative or caregiver
O Only authorised representative or caregiver
O Partially participant and information from medical file
O Partially authorised representative or caregiver and information from medical file
O Information from medical file

## 8. Consumption of addictive substances [T0]

***In the case of severe cognitive impairment, the questions must be completed with the support of the authorised representative or caregiver.***

**Status**
O Questionnaire completely answered
O Questionnaire partially answered or survey cancelled
O Participant refuses to answer the questions
O Questions could not be answered
O Other/comment: ________________

### Smoking behaviour

1. **Are you a smoker?**
   O Yes O No

   **If yes, how many cigarettes, cigars or pipes do you currently smoke?**
   ___ (number)
   O per day
   O per week
   O per month
   O per year

   **If you do not currently smoke: Did you smoke in the past?**
   O Yes O No

   **How old were you when you started smoking?**
   ___ years

   **If a former smoker: How many years/months ago did you stop smoking?**
   ___ years and/or
   ___ months

### Alcohol consumption

"Next, I would like to ask you a few questions about your alcohol consumption. This question is very important for us because alcohol affects your state of health. And the state of health can change after discharge."

2. **Do you drink alcohol**
   O Yes O No

   **If no, did you drink alcohol in the past?**
   O Yes O No => please go to the section "Questions were answered by/collected from"

   **When did you stop drinking alcohol? Since ...**
   ___ years and/or
   ___ months

**3. If yes, how often do you drink alcoholic beverages?**

| | Never | Less than once a month | 1-3 times a month | 1-4 times per week | 5-6 times per week | Daily |
|---|---|---|---|---|---|---|
| **Beer** | O | O | O | O | O | O |
| **Wine/sparkling wine** | O | O | O | O | O | O |
| **Spirits** | O | O | O | O | O | O |

**4. How many glasses do you drink in a normal week?**
___ glasses/week

**5. How often do you drink 3 or more alcoholic drinks at a time**
O Never
O Less than once a month
O 1-3 times a month
O 1-4 times a week
O 5-6 times a week
O Daily

**6. How long has the above-mentioned drinking behaviour existed?**
___ years and/or
___ months

**Questions were answered by/collected from:**
O Participant
O Partially participant and authorised representative or caregiver
O Only authorised representative or caregiver
O Partially participant and information from medical file
O Partially authorised representative or caregiver and information from medical file
O Information from medical file

## 9. Hand strength with dynamometer [T0]

**Status**
O Dynamometer completely performed
O Dynamometer partially performed or cancelled
O Participant refuses dynamometer
O Dynamometer not possible for participant
O Other/comment: ________________

**Instructions:**
***"I would like to measure your hand strength with this device. Take it in your right hand. Is it comfortable in your hand?"***
***"Please bend your right arm at a 90 degree angle."***

**Note:** Participant sits on the chair, arms not propped up, shoulders not raised, no "equalising movements". If necessary, measure while lying down, here arm bent in the same 90° position.

***"When I say go, squeeze the handle as hard as you can." => "Go!"***
Repeat accordingly with the other measurements

1. **Measurement - Right hand**
   **1st attempt** ___ kg
   **2nd attempt** ___ kg

   **Measurement takes place**
   O Sitting
   O Lying down

   **Remark**
   O No comment
   O Disability in the respective arm
   O Participant refuses measurement
   O Other/comment: _______________

2. **Measurement - Left hand**
   **1st attempt** ___ kg
   **2nd attempt** ___ kg

   **Measurement takes place**
   O Sitting
   O Lying down

   **Remark**
   O No comment
   O Disability in the respective arm
   O Participant refuses measurement
   O Other/comment: _______________

3. **Which is your dominant hand?**
   **(e.g. which hand do you use to comb your hair, brush your teeth, ...?)**
   O Right hand
   O Left hand
   O Both about the same

## 10. Montreal Cognitive Assessment (MoCA) - initial examination [T0]

**Status MoCA examination**
O MoCA - Initial examination completed
O MoCA - Initial investigation partially completed or cancelled
O Participant refuses MoCA
O MoCA not possible for participant
O Other/comment: _____________

☐ **Version for severely impaired/absent vision used?**
**(please start at section 'Memory')**

***Details on the MoCA, the test version 7.1 and the corresponding instructions can be found in references (4) and (5).***

## 11. Montreal Cognitive Assessment (MoCA) - second examination [T1]

**Status MoCA examination**
O MoCA - Second examination completed
O MoCA - Second examination partially completed or cancelled
O Participant refuses MoCA
O MoCA not possible for participant
O Other/comment: _____________

☐ **Version for severely impaired/absent vision used?**
**(please start at section 'Memory')**

**If not performed at T1, date and time of performance:**
___.___.______ _____:_____ dd.mm.yyyy hh:mm

***Details on the MoCA, the test version 7.2 and the corresponding instructions can be found in references (4) and (5).***

## 12. Montreal Cognitive Assessment (MoCA) - third examination [T3, until 16 March 2020]

**Status MoCA examination**
O MoCA - Third examination completed
O MoCA - Third examination partially completed or cancelled
O Participant refuses MoCA
O MoCA not possible for participant
O Other/comment: _____________

☐ **Version for severely impaired/absent vision used?**
**(please start at section 'Memory')**

***Details on the MoCA, the test version 7.3 and the corresponding instructions can be found in references (4) and (5).***

## 13. Telephone Montreal Cognitive Assessment (T-MoCA) [T3, from 16.03.2020]

**Status**
O T-MoCA - completed
O T-MoCA – partially completed or cancelled
O Participant refuses T-MoCA
O T-MoCA not possible for participant
O Other/comment: _________________

***Important note: Please speak particularly slowly, clearly and loudly so that everything is clearly understood, and pay particular attention when registering the tapping noises in the letter list!***

***Details on the T-MoCA, the test version 7.3 and the corresponding instructions can be found in references (6), (7) and (5).***

## 14. ICD-10 adapted Confusion Assessment Method Severity Score (I-CAM-S)

### (I-CAM including severity classification of delirium) [T0, T1, T2, T3]

**Status**
O I-CAM-S carried out completely
O I-CAM-S partially performed or cancelled
O Participant refuses I-CAM-S
O I-CAM-S not possible for participant
O Other/comment: _____________

***Details on the I-CAM-S can be found in references (8), (9), (10) and (11).***

**Questions 3-5 were answered by:**
O Nurse
O Authorised representative or caregiver

## 15. Rivermead Mobility Index [T0, T1, T2, T3]

**Status**
O Questionnaire completely answered
O Questionnaire partially answered or survey cancelled
O Participant refuses to answer the questions
O Questions could not be answered
O Other/comment: ________________

***The Rivermead Mobility Index questions and instructions can be found in references (12) and (13).***

**Questions were answered by:**
O Participant
O Partially participant and authorised representative or caregiver
O Only authorised representative or caregiver

## 16. Patient Health Questionnaire 4 (PHQ-4) [T0]

**Status**
O Questionnaire completely answered
O Questionnaire partially answered or survey cancelled
O Participant refuses to answer the questions
O Questions could not be answered
O Other/comment: _________________

***The PHQ-4 questions and instructions can be found in references (14) and (15).***

## 17. Awareness of Age Related Change (AARC) [T0]

**Status**
O Questionnaire completely answered
O Questionnaire partially answered or survey cancelled
O Participant refuses to answer the questions
O Questions could not be answered
O Other/comment: _________________

***The AARC questions and instructions can be found in reference (16).***

## 18. General Self-Efficacy Short Scale (Allgemeine Selbstwirksamkeit Kurzskala, ASKU) [T0]

**Status**
O Questionnaire completely answered
O Questionnaire partially answered or survey cancelled
O Participant refuses to answer the questions
O Questions could not be answered
O Other/comment: _________________

***The ASKU questions and instructions can be found in reference (17).***

## 19. Simplified nutritional appetite questionnaire (SNAQ) [T0, T2, T3]

**Status**
O Questionnaire completely answered
O Questionnaire partially answered or survey cancelled
O Participant refuses to answer the questions
O Questions could not be answered
O Other/comment: _________________

***The SNAQ questions and instructions can be found in reference (18).***

## 20. Pittsburgh Sleep Quality Index (PSQI Basic) [T0]

***In the case of severe cognitive impairment, the questions must be completed with the support of the authorised representative or caregiver.***

**Status**
O Questionnaire completely answered
O Questionnaire partially answered or survey cancelled
O Participant refuses to answer the questions
O Questions could not be answered
O Other/comment: ________________

***The PSQI questions and instructions can be found in reference (19) (TRADE used items #1, #2, #3, #4, #6, #7).***

**Questions were answered by:**
O Participant
O Partially participant and authorised representative or caregiver
O Only authorised representative or caregiver

## 21. Follow-up questions for participants - Discharge and new place of residence [T1]

**Status**
O Questionnaire completely answered
O Questionnaire partially answered or survey cancelled
O Participant refuses to answer the questions
O Questions could not be answered
O Other/comment: ________________

1. **Did you know where you were being transferred to?**
   O Yes O No

2. **When did you know the discharge date (date and whether discharge is in the morning, at midday, in the afternoon or in the evening)?**
   O Not at all
   O Very shortly before < 30 minutes
   O A few hours before
   O 1 day before
   O Several days before

3. **Was anything lost or not taken with you during the transfer?**
   O Yes O No
   If yes, what? ____________________________________________

4. **Were you spoken to in the last few days before discharge about your discharge and further care?**
   O Yes O No

   *If yes, when were you spoken to?*
   _____ days before
   _____ hours before
   *If yes, who did you speak to? (multiple answers possible)*
   O Discharge manager
   O Case manager
   O Nurse
   O Doctor

O Other professional group: ________________________________________

5. **How well did you feel about your discharge from hospital?**
Please indicate on a scale from 0 to 10: 0= very bad, 10= very good
**Note**: Please show visual scale no. 2.
O 0 O 1 O 2 O 3 O 4 O 5 O 6 O 7 O 8 O 9 O 10
**What did you find particularly good? Please briefly describe the most important point.**
______________________________________________________________________________
______________________________________________________________________________
**What did you find particularly bad? Please briefly describe the most important point.**
______________________________________________________________________________
______________________________________________________________________________

6. **Which means of transport did you use to get from the hospital to your new current location (or home)?**
O Ambulance O Taxi O Car (with relatives) O Car (self-driven) O Bus/train
O Transport for disabled persons O Other: ______________________________

7. **Was someone with you <u>during discharge</u> (before transport) from hospital?** O Yes
O No
***If yes, who was present?***

O Partner
O Sister
O Brother
O Daughter
O Son
O Daughter-in-law
O Son-in-law
O Granddaughter
O Grandson
O Niece
O Nephew
O Friend
O Neighbour
O Other: ______________________

8. **Was it possible to be accompanied <u>during the transport</u>?**
O Yes O No
**Did anyone accompany you?**

O No accompaniment
O Partner
O Sister
O Brother
O Daughter
O Son
O Daughter-in-law
O Son-in-law
O Granddaughter
O Grandson
O Niece
O Nephew
O Friend
O Neighbour
O Other: ______________________

***If there was no accompaniment during the transport, what was the reason?***
O No time due to work
O Information about discharge was too short notice
O Too far away from the hospital
O Not aware of the possibility of an accompaniment
O Other: ______________________

Project: TRADE observational study | Date ___.___.______

Timepoint: O T0 O T1 O T2 O T3 | Interviewer code number: _____

Centre: _______ | Participant ID: _____

**9. Has anyone been with you since your admission to this current facility (or home)?**
O Yes O No

***If yes, who and for how long have the person(s) been with you in the last few days?***

| | Not available/ was not there | Very short, < 30 min. | 30 min. to 2 hours | 2 to 4 hours | 4 to 8 hours | 8 to 24 hours | over 24 hours | Was there, time unclear |
|---|---|---|---|---|---|---|---|---|
| Partner | O | O | O | O | O | O | O | O |
| Sister | O | O | O | O | O | O | O | O |
| Brother | O | O | O | O | O | O | O | O |
| Daughter | O | O | O | O | O | O | O | O |
| Son | O | O | O | O | O | O | O | O |
| Daughter-in-law | O | O | O | O | O | O | O | O |
| Son-in-law | O | O | O | O | O | O | O | O |
| Granddaughter | O | O | O | O | O | O | O | O |
| Grandson | O | O | O | O | O | O | O | O |
| Niece | O | O | O | O | O | O | O | O |
| Nephew | O | O | O | O | O | O | O | O |
| Friend | O | O | O | O | O | O | O | O |
| Neighbour | O | O | O | O | O | O | O | O |
| Other | O | O | O | O | O | O | O | O |

If other or more, please specify person(s) and duration:

____________________________________________________________________

***If yes, at what time of day did the person(s) named above visit you?***

| | Not avai-lable/ was not there | Before noon | Lunch-time | After-noon | Evening / At night | Almost always during the day | Almost always at night | Almost always days and at night | Was there, time unclear |
|---|---|---|---|---|---|---|---|---|---|
| Partner | O | O | O | O | O | O | O | O | O |
| Sister | O | O | O | O | O | O | O | O | O |
| Brother | O | O | O | O | O | O | O | O | O |
| Daughter | O | O | O | O | O | O | O | O | O |
| Son | O | O | O | O | O | O | O | O | O |
| Daughter-in-law | O | O | O | O | O | O | O | O | O |
| Son-in-law | O | O | O | O | O | O | O | O | O |
| Granddaughter | O | O | O | O | O | O | O | O | O |
| Grandson | O | O | O | O | O | O | O | O | O |
| Niece | O | O | O | O | O | O | O | O | O |
| Nephew | O | O | O | O | O | O | O | O | O |
| Friend | O | O | O | O | O | O | O | O | O |
| Neighbour | O | O | O | O | O | O | O | O | O |
| Other | O | O | O | O | O | O | O | O | O |

If other or more, please specify person(s) and time period:

____________________________________________________________________

**10. Where were you discharged to?**
O Home
O To a known facility where you have previously been for at least 6 months
O To a known facility where you have previously been for less than 6 months
O To a new, unknown facility where you have never been before

11. **How welcome did you feel in the environment you were discharged to?** Please indicate on a scale from 0 to 10: 0= not at all welcome, 10= very welcome
    **Note**: Please show visual scale no. 2.
    O 0 O 1 O 2 O 3 O 4 O 5 O 6 O 7 O 8 O 9 O 10

12. **How well do you find your way around here?**
    Please indicate on a scale from 0 to 10: 0= not at all, 10 = very good
    **Note**: Please show visual scale no. 2.
    O 0 O 1 O 2 O 3 O 4 O 5 O 6 O 7 O 8 O 9 O 10

13. **Do you have the impression that the most important medical information (doctor's letter, medication, prescriptions for aids/medication, etc.) was given to you on discharge?**
    O Yes O No
    ***If no, what information was missing?*** *(multiple answers possible)*
    O Doctor's letter
    O Doctor's letter was there, but contents were missing/incorrect
    O Prescription for medication
    O Prescription for medical aids
    O Other problems: ________________________

14. **Do you have the impression that the carers received the most important care-related information (nursing report, level of care, amount of care required, etc.)?**
    O Yes O No
    ***If no, what information was missing?*** *(multiple answers possible)*
    O Nursing report
    O Need for care incorrectly assessed
    O Handover did not take place
    O No contact details available for consultation
    O Other problems: ________________________

15. **How good did you find the reception at your new place of residence (or at home)?**
    Please indicate on a scale from 0 to 10: 0= very bad, 10= very good
    **Note**: Please show visual scale no. 2.
    O 0 O 1 O 2 O 3 O 4 O 5 O 6 O 7 O 8 O 9 O 10
    **What did you find particularly good? Please briefly describe the most important point.**
    ______________________________________________
    ______________________________________________

    **What did you find particularly bad? Please briefly describe the most important point.**
    ______________________________________________
    ______________________________________________

## 22. Follow-up questions participants - health status [T1, T2, T3]

**Status**
O Questionnaire completely answered
O Questionnaire partially answered or survey cancelled
O Participant refuses to answer the questions
O Questions could not be answered
O Other/comment: ________________

1. **Has there been a change in your care level since our last contact?**
   O Yes O No
   ***If yes, what care level do you currently have?***
   O Care level 1 O Care level 2 O Care level 3 O Care level 4 O Care level 5

2. **How well can you concentrate at the moment**
   Please indicate on a scale from 0 to 10: 0= very poor ability to concentrate, 10= very good ability to concentrate
   **Note**: Please show visual scale no. 2.
   O 0 O 1 O 2 O 3 O 4 O 5 O 6 O 7 O 8 O 9 O 10

3. **Has your ability to concentrate deteriorated since our last contact?** O
   Yes O No

4. **Do you currently feel or have you at any time since our last contact felt more tired than usual?**
   O Yes O No

5. **Do you currently feel or have you felt anxious at any time since our last contact?**
   O Yes O No

6. **Do you currently or at any time since our last contact find structured thinking more difficult than usual?**
   O Yes O No

7. **How do you currently sleep**
   Please indicate on a scale from 0 to 10: 0= very poor sleep quality, 10= very good sleep quality
   **Note**: Please show visual scale no. 2.
   O 0 O 1 O 2 O 3 O 4 O 5 O 6 O 7 O 8 O 9 O 10
   ***If rather poor or poor quality of sleep, what are your problems with?***
   (multiple answers possible)
   O Sleeping through the night
   O Falling asleep
   O Orientation during the night
   O Hallucinations during the night
   O Other: _________________________

8. **Have you fallen since our last contact?**
   O Yes O No
   ***If yes, how often?***
   _____ times

9. **Has your state of health changed since our last contact?**
   O Yes, improved O Yes, worsened O No
   ***If yes, improved: how much***
   Please indicate on a scale from 0 to 10: 0= not improved at all, 10= very much improved
   **Note**: Please show visual scale no. 2.
   O 0 O 1 O 2 O 3 O 4 O 5 O 6 O 7 O 8 O 9 O 10
   ***If yes, deteriorated: how much***
   Please indicate on a scale from 0 to 10: 0= not deteriorated at all, 10= very much deteriorated
   **Note**: Please show visual scale no. 1
   O 0 O 1 O 2 O 3 O 4 O 5 O 6 O 7 O 8 O 9 O 10
   ***If your state of health has changed, what do you attribute the change to?*** (multiple answers possible)
   O Communication/interpersonal skills

O Mobility
O Activity
O Drive
O Appetite
O Satisfaction
O Interest
O Mood/emotion
O Concentration
O Need for support
O Pain
O Shortness of breath
O Infection (e.g. bladder infection or pneumonia)
O Other: ____________________

**[T3] 10. ONLY at timepoint T3: Have you been hospitalised again since the beginning of the study?**
O Yes O No

**[T3] ONLY at timepoint T3: If yes, please specify facility, reason for stay and time period:**

| | Facility | Reason | From [dd.mm.yyyy] | To [dd.mm.yyyy] |
|---|---|---|---|---|
| 1. | ____________________ | ____________________ | ___.___._____ | ___.___._____ |
| 2. | ____________________ | ____________________ | ___.___._____ | ___.___._____ |
| 3. | ____________________ | ____________________ | ___.___._____ | ___.___._____ |

For further stays: please specify in the comment field

**[T3] 11. ONLY at timepoint T3: Are there any new diagnoses since discharge from the hospital?**
O Yes O No
***If yes, which one(s)?***

______________________________________________
______________________________________________
______________________________________________
______________________________________________

**[T3] 12. <u>ONLY at timepoint T3</u>: In the following you are asked about your social contacts: With whom and how often do you have contact in your familiar surroundings?**

| | Not available | Daily | Several times a week | Once a week | Several times a month | Once a month | Rarer |
|---|---|---|---|---|---|---|---|
| Partner | O | O | O | O | O | O | O |
| Sister | O | O | O | O | O | O | O |
| Brother | O | O | O | O | O | O | O |
| Daughter | O | O | O | O | O | O | O |
| Son | O | O | O | O | O | O | O |
| Daughter-in-law | O | O | O | O | O | O | O |
| Son-in-law | O | O | O | O | O | O | O |
| Granddaughter | O | O | O | O | O | O | O |
| Grandson | O | O | O | O | O | O | O |
| Niece | O | O | O | O | O | O | O |
| Nephew | O | O | O | O | O | O | O |
| Friend | O | O | O | O | O | O | O |
| Neighbour | O | O | O | O | O | O | O |
| Other | O | O | O | O | O | O | O |

If other or more, please specify person(s) and frequency:

______________________________________________________

______________________________________________________

[T3] ***Please update the medication list with the study participant now (under Nurse/Medical file)***

## C. Questions for the CAREGIVER

**[T1]** ***Note: Please check whether <u>the IADL questionnaire</u> was conducted at T0. If not yet carried out, please carry out the IADL questionnaire if the consent form of the caregiver is available.***

## 23. Instrumental activities of daily living according to Lawton (IADL) [T0, T3]

**Status**
O Questionnaire completely answered
O Questionnaire partially answered or survey cancelled
O Caregiver refuses to answer the questions
O Caregiver not available
O No caregiver available
O Other/comment: ________________

**[T0] Date and time of interview**
___.___.______ ____:____ dd.mm.yyyy hh:mm

***The Lawton IADL scale questions and instructions can be found in references (20) and (21).***

**The following questions concern the social contacts of Mr/Mrs ...: With whom and how often does Mr/Mrs ... have contact in his/her familiar surroundings?**

| | Not available | Daily | Several times a week | Once a week | Several times a month | Once a month | Rarer |
|---|---|---|---|---|---|---|---|
| Partner | O | O | O | O | O | O | O |
| Sister | O | O | O | O | O | O | O |
| Brother | O | O | O | O | O | O | O |
| Daughter | O | O | O | O | O | O | O |
| Son | O | O | O | O | O | O | O |
| Daughter-in-law | O | O | O | O | O | O | O |
| Son-in-law | O | O | O | O | O | O | O |
| Granddaughter | O | O | O | O | O | O | O |
| Grandson | O | O | O | O | O | O | O |
| Niece | O | O | O | O | O | O | O |
| Nephew | O | O | O | O | O | O | O |
| Friend | O | O | O | O | O | O | O |
| Neighbour | O | O | O | O | O | O | O |
| Other | O | O | O | O | O | O | O |

If other or more, please specify person(s) and frequency:

______________________________________________

______________________________________________

## 24. Follow-up questions caregiver - Discharge and new place of residence [T1]

**Status**
O Questionnaire completely answered
O Questionnaire partially answered or survey cancelled
O Caregiver refuses to answer the questions
O Caregiver not available
O No caregiver available
O Other/comment: ________________

**[T0] Date and time of interview**
___.___.______ ____:____ dd.mm.yyyy hh:mm

**Start interview from here**

1. **Did you know where Mr/Mrs ... was being transferred to?**
O Yes O No

2. **When did you know the exact discharge date (date and whether discharge was in the morning, at midday, in the afternoon or in the evening)?**
O Not at all
O Very shortly before < 30 minutes
O A few hours before
O 1 day before
O Several days before

3. **Was anything lost or not taken with Mr/Mrs … during the transfer?**
O Yes O No
If yes, what? ____________________________________________

4. **Were you spoken to in the last few days before discharge about Mr/Mrs …'s discharge and further care?**
O Yes O No
***If yes, when were you spoken to?***
_____ days before
_____ hours before
***If yes, who did you speak to? (multiple answers possible)***
O Discharge manager
O Case manager
O Nurse
O Doctor
O Other professional group: ____________________________________________

5. **How well did you feel about the discharge of Mr/Mrs ... from hospital?**
Please indicate on a scale from 0 to 10: 0= very bad, 10= very good
O 0 O 1 O 2 O 3 O 4 O 5 O 6 O 7 O 8 O 9 O 10

**What did you find particularly good? Please briefly describe the most important point.**
______________________________________________________________________
______________________________________________________________________
______________________________________________________________________

**What did you find particularly bad? Please briefly describe the most important point.**
______________________________________________________________________
______________________________________________________________________
______________________________________________________________________

6. **By which means of transport did Mr/Mrs ... get from the hospital to the new current location (or home)?**

O Ambulance O Taxi O Car (with relatives) O Car (self-driven) O Bus/train
O Transport for disabled persons O Other: ________________________________

**7. Was anyone with Mr/Mrs ... <u>during discharge</u> (before transport) from hospital?**

O Yes O No

***If yes, who was with him/her?***

| | |
|---|---|
| O Partner | O Granddaughter |
| O Sister | O Grandson |
| O Brother | O Niece |
| O Daughter | O Nephew |
| O Son | O Friend |
| O Daughter-in-law | O Neighbour |
| O Son-in-law | O Other: ______________________ |

**8. Was there the possibility of an accompaniment <u>during the transport</u>?**

O Yes O No

**Did anyone accompany Mr/Mrs ... ?**

| | |
|---|---|
| O No accompaniment | O Granddaughter |
| O Partner | O Grandson |
| O Sister | O Niece |
| O Brother | O Nephew |
| O Daughter | O Friend |
| O Son | O Neighbour |
| O Daughter-in-law | O Other: ______________________ |
| O Son-in-law | |

***If Mr/Mrs … was not accompanied during the transport, what was the reason?***

O No time due to work
O Information about discharge was too short notice
O Too far away from the hospital
O Not aware of the possibility of an accompaniment
O Other: ______________________

***If there had been the option of accompanying Mr/Mrs … during transport, would you have used it?***

O Yes O No

Project: TRADE observational study | Date ___.___.______

Timepoint: O T0 O T1 O T2 O T3 | Interviewer code number: _____

Centre: _______ | Participant ID: _____

**9. Has anyone been in this current facility (or at home) with Mr/Mrs ... since admission?**

O Yes O No

*If yes, who and for how long in total has/have the person(s) been with Mr/Mrs ... in the last few days?*

| | Not available/ was not there | Very short, < 30 min. | 30 min. to 2 hours | 2 to 4 hours | 4 to 8 hours | 8 to 24 hours | over 24 hours | Was there, time unclear |
|---|---|---|---|---|---|---|---|---|
| Partner | O | O | O | O | O | O | O | O |
| Sister | O | O | O | O | O | O | O | O |
| Brother | O | O | O | O | O | O | O | O |
| Daughter | O | O | O | O | O | O | O | O |
| Son | O | O | O | O | O | O | O | O |
| Daughter-in-law | O | O | O | O | O | O | O | O |
| Son-in-law | O | O | O | O | O | O | O | O |
| Granddaughter | O | O | O | O | O | O | O | O |
| Grandson | O | O | O | O | O | O | O | O |
| Niece | O | O | O | O | O | O | O | O |
| Nephew | O | O | O | O | O | O | O | O |
| Friend | O | O | O | O | O | O | O | O |
| Neighbour | O | O | O | O | O | O | O | O |
| Other | O | O | O | O | O | O | O | O |

If other or more, please specify person(s) and duration:

______________________________________________________________

*If yes, at what time of day did the person(s) named above visit Mr/Mrs ...? visit?*

| | Not avai-lable/ was not there | Before noon | Lunch-time | After-noon | Evening/ At night | Almost always during the day | Almost always at night | Almost always days and at night | Was there, time un-clear |
|---|---|---|---|---|---|---|---|---|---|
| Partner | O | O | O | O | O | O | O | O | O |
| Nurse | O | O | O | O | O | O | O | O | O |
| Brother | O | O | O | O | O | O | O | O | O |
| Daughter | O | O | O | O | O | O | O | O | O |
| Son | O | O | O | O | O | O | O | O | O |
| Daughter-in-law | O | O | O | O | O | O | O | O | O |
| Son-in-law | O | O | O | O | O | O | O | O | O |
| Granddaughter | O | O | O | O | O | O | O | O | O |
| Grandson | O | O | O | O | O | O | O | O | O |
| Niece | O | O | O | O | O | O | O | O | O |
| Nephew | O | O | O | O | O | O | O | O | O |
| Friend | O | O | O | O | O | O | O | O | O |
| Neighbour | O | O | O | O | O | O | O | O | O |
| Other | O | O | O | O | O | O | O | O | O |

If other or more, please specify person(s) and time period:

______________________________________________

**10. Where was Mr/Mrs ... discharged to?**

O Home
O To a known institution where Mr/Mrs ... was previously there for at least 6 months
O To a known facility where Mr/Mrs ... was previously there for less than 6 months
O To a new, unknown facility where Mr/Mrs ... has never been before

**11. How well is Mr/Mrs ... in the environment to which he/she has been transferred?**

Please indicate on a scale from 0 to 10: 0= not at all, 10 = very good

O 0 O 1 O 2 O 3 O 4 O 5 O 6 O 7 O 8 O 9 O 10

**12. Do you have the impression that the most important medical information (doctor's letter, medication, prescriptions for aids/medication, etc.) was given to Mr/Mrs … and/or you on his/her discharge?**

O Yes O No

***If no, what information was missing? (multiple answers possible)***

O Doctor's letter
O Doctor's letter was there, but contents were missing/incorrect
O Prescription for medication
O Prescription for medical aids
O Other problems: ________________________

**13. Do you have the impression that the carers received the most important care-related information (nursing report, level of care, amount of care required, etc.)?**

O Yes O No

***If no, what information was missing? (multiple answers possible)***

O Nursing report
O Need for care incorrectly assessed
O Handover did not take place
O No contact details available for consultation
O Other problems: ________________________

**14. How well did you find the reception of Mr/Mrs ... at his/her new current place of residence (or at home)?**

Please indicate on a scale from 0 to 10: 0= very bad, 10= very good

O 0 O 1 O 2 O 3 O 4 O 5 O 6 O 7 O 8 O 9 O 10

**What did you find particularly good? Please briefly describe the most important point.**

______________________________________________________________________
______________________________________________________________________
______________________________________________________________________

**What did you find particularly bad? Please briefly describe the most important point.**

______________________________________________________________________
______________________________________________________________________
______________________________________________________________________

Project: TRADE observational study | Date ___.___.______
Timepoint: O T0 O T1 O T2 O T3 | Interviewer code number: _____
Centre: _______ | Participant ID: _____

## 25. Follow-up questions caregiver - health status [T1, T2, T3]

**Status**
O Questionnaire completely answered
O Questionnaire partially answered or survey cancelled
O Caregiver refuses to answer the questions
O Caregiver not available
O No caregiver available
O Other/comment: ________________

**[T0] Date and time of interview**
___.___.______ ____:____ dd.mm.yyyy hh:mm

**What is the relationship between the caregiver and the participant?**
O Partner
O Sister
O Brother
O Daughter
O Son
O Daughter-in-law
O Son-in-law
O Granddaughter
O Grandson
O Niece
O Nephew
O Friend
O Neighbour
O Other: ________________________

**Has there been a change of caregiver?**
O Yes O No, same caregiver
***If yes, is a signed declaration of consent from the current caregiver available?***
O Yes O No => separate interview with the caregiver not possible!
***If yes, was the current caregiver included in the study participant identification list?***
O Yes O No => separate interview with the caregiver not possible!

**<u>Start survey from here</u>**

1. **Has Mr/Mrs ...'s state of health changed since our last contact?**
   O Yes, improved O Yes, worsened O No
   ***If yes, improved: how much***
   Please indicate on a scale from 0 to 10: 0= not at all improved, 10= very much improved
   O 0 O 1 O 2 O 3 O 4 O 5 O 6 O 7 O 8 O 9 O 10
   ***If yes, worsened: how much***
   Please indicate on a scale from 0 to 10: 0= not deteriorated at all, 10= very much deteriorated
   O 0 O 1 O 2 O 3 O 4 O 5 O 6 O 7 O 8 O 9 O 10
   ***If Mr/Mrs ...'s state of health has changed, what do you attribute the change to?*** (multiple answers possible)
   O Communication/interpersonal skills
   O Mobility
   O Activity
   O Drive
   O Appetite
   O Satisfaction
   O Interest
   O Mood/emotion
   O Concentration
   O Need for support
   O Pain
   O Shortness of breath
   O Infection (e.g. cystitis or pneumonia)
   O Other: ________________________

***2.*** **Do you have the impression that Mr/Mrs ... is/was confused since our last contact?**
O Yes O No
***If yes, to what extent?***
Please indicate on a scale from 0 to 10: 0= not confused at all, 10= very confused
O 0 O 1 O 2 O 3 O 4 O 5 O 6 O 7 O 8 O 9 O 10
***Is the confusion fluctuating (sometimes better and sometimes worse)?***
O Yes, fluctuates O No, always stays the same

**3. How would you rate Mr/Mrs ...'s ability to concentrate since our last contact?**
Please indicate on a scale from 0 to 10: 0= very poor ability to concentrate, 10= very good ability to concentrate
O 0 O 1 O 2 O 3 O 4 O 5 O 6 O 7 O 8 O 9 O 10

**4. Have you or other people noticed the following unusual behaviour in Mr/Mrs ...?** (multiple answers possible)
O Running/runaway tendency (seemingly wandering around aimlessly)
O Sleep disturbance
O Restlessness in the evening
O Screaming
O Trying to get out of bed in inappropriate situations
O Nodding
O Verbally aggressive
O Physically aggressive
O Visual hallucinations/deceptive images
O Other: _________________________

**5. Since our last contact, has Mr/Mrs ... required further hospitalisation or contact with a doctor?**
O Yes O No
***If yes, why?***
O Known medical problem worsened
O New medical problem occurred
O Change of medication
O Other: _________________________

**6. Has Mr/Mrs ... fallen since our last contact?**
O Yes O No
If yes, how often?
____ times

## 26. Family CAM (FAM-CAM) [T1, T2, T3]

**Status**
O Questionnaire completely answered
O Questionnaire partially answered or survey cancelled
O Caregiver refuses to answer the questions
O Caregiver not available
O No caregiver available
O Other/comment: ________________

***The FAM-CAM questions and instructions can be found in references (22) and (23).***

**[T3, as of 16 March 2020]**
***Attention: Please still fill in the Nu-DESC and Barthel index with the caregiver (under Nurse/Medical file)!***

## 27. Informant Questionnaire on COgnitive Decline in the Elderly (IQCODE) [T1]

**Status**
O Questionnaire completely answered
O Questionnaire partially answered or survey cancelled
O Caregiver refuses to answer the questions
O Caregiver not available
O No caregiver available
O Other/comment: ________________

**If not performed at T1, date and time of interview:**
___.___.______ _____:_____ dd.mm.yyyy hh:mm

***The IQCODE questions and instructions can be found in references (24), (25) and (26) (TRADE used the German very short form consisting of items #3, #4, #5, #8, #10, #13, #24 of the long form).***

Project: TRADE observational study | Date ___.___.______

Timepoint: O T0 O T1 O T2 O T3 | Interviewer code number: _____

Centre: _______ | Participant ID: _____

## D. Questions for NURSE/ information from the MEDICAL FILE

### 28. Nursing Delirium Screening Scale (Nu-DESC) [T0, T1, T2, T3]

[T3, from 16.03.2020] ***Attention: From 16.03.2020, the interview will be conducted with the caregiver by telephone!***

**Status**
O Questionnaire completely answered
O Questionnaire partially answered or survey cancelled
O Answering the questions by nurse not possible
O For telephone interviews: Answering the questions by caregiver not possible
O Other/comment: _________________

**Note:** Assessment refers to the period of the last 24 hours

***The Nu-DESC questions and instructions can be found in references (27) and (28).***

[T0] **Supplementary question at T0:**
**Did the participant experience acute cognitive deterioration or delirium during hospitalisation?**
O Yes O No

**Questions were answered by/collected from:**
O Nurse who knew the participant
O Nurse who did not know the participant
O Information from medical file
O Caregiver (telephone interview)

### 29. Barthel Index (according to the Hamburg Manual) [T0, T3]

[T3, from 16.03.2020] ***Attention: From 16.03.2020, the interview will be conducted by telephone by the caregiver!***

**Status**
O Questionnaire completely answered
O Questionnaire partially answered or survey cancelled
O Barthel Index not available in medical file and nurse unable to answer questions
O For telephone interviews: Answering the questions by caregiver not possible
O Other/comment: ______________________

***The Barthel Index questions and instructions can be found in references (29) and (30)***

### 30. Frailty scale (CSHA Clinical Frailty Scale) [T0]

***Please use the "Frailty Scale" assessment form***

**Participant is ... ?**
O very fit
O well
O managing well
O vulnerable
O mildly frail
O moderately frail
O severely frail
O very severely frail
O terminally ill

## 31. Follow-up questions for nurses - new location [T1]

**Status**
O Questionnaire completely answered
O Questionnaire partially answered or survey cancelled
O Answering the questions by nurse not possible
O Other/comment: ____________________

1. **Was the specified admission time correct (+/- 2h)?**
   O Yes
   O No, other day
   O No, other time

2. **Do you have the impression that the most important medical information (doctor's letter, medication, prescriptions for aids/medication, etc.) was provided on discharge?**
   O Yes O No
   *If no, what information was missing? (multiple answers possible)*
   O Doctor's letter
   O Doctor's letter was there, but contents were missing/incorrect
   O Prescription for medication
   O Prescription for medical aids
   O Other problems: ________________________

3. **Do you have the impression that the carers received the most important care-related information (nursing report, level of care, amount of care required, etc.)?**
   O Yes O No
   *If no, what information was missing? (multiple answers possible)*
   O Nursing report
   O Need for care incorrectly assessed
   O Handover did not take place
   O No contact details available for consultation
   O Other problems: ________________________

4. **How well does Mr/Mrs ... find his/her way around his/her new place of residence**
   Please indicate on a scale from 0 to 10: 0= not at all, 10 = very good
   O 0 O 1 O 2 O 3 O 4 O 5 O 6 O 7 O 8 O 9 O 10

5. **How much time do you estimate it took to admit Mr/Mrs … to this facility?**
   (incl. administrative organisation such as creating patient files etc., procuring medication and aids, setting up the room, showing the facility, providing information about the daily and weekly routine, ...)
   ____ Hours

## 32. Follow-up questions for nurses - health status [T1, T2, T3]

**Status**
O Questionnaire completely answered
O Questionnaire partially answered or survey cancelled
O Answering the questions by nurse not possible
O Other/comment: ____________________

1. **Has Mr/Mrs ...'s state of health changed since our last contact?**
   O Yes, improved O Yes, worsened O No
   ***If yes, improved: how much?***
   Please indicate on a scale from 0 to 10: 0= not improved at all, 10= very much improved
   O 0 O 1 O 2 O 3 O 4 O 5 O 6 O 7 O 8 O 9 O 10
   ***If yes, worsened: how much***
   Please indicate on a scale from 0 to 10: 0= not deteriorated at all, 10= very much deteriorated
   O 0 O 1 O 2 O 3 O 4 O 5 O 6 O 7 O 8 O 9 O 10
   ***If Mr/Mrs …'s state of health has changed, what do you think the change is due to?*** (multiple answers possible)
   O Communication/interpersonal skills
   O Mobility
   O Activity
   O Drive
   O Appetite
   O Satisfaction
   O Interest
   O Mood/emotion
   O Concentration
   O Need for support
   O Pain
   O Shortness of breath
   O Infection (e.g. bladder infection or pneumonia)
   O Other: ________________________

2. **Do you have the impression that Mr/Mrs ... is/was confused since our last contact?**
   O Yes O No
   ***If yes, how much***
   Please indicate on a scale from 0 to 10: 0= not confused at all, 10= very confused O 0
   O 1 O 2 O 3 O 4 O 5 O 6 O 7 O 8 O 9 O 10
   ***Is the confusion fluctuating (sometimes better and sometimes worse)?***
   O Yes, fluctuates O No, always stays the same

3. **How would you rate Mr/Mrs ...'s ability to concentrate since our last contact?**
   Please indicate on a scale from 0 to 10: 0= very poor ability to concentrate, 10= very good ability to concentrate
   O 0 O 1 O 2 O 3 O 4 O 5 O 6 O 7 O 8 O 9 O 10

4. **Have you or other people noticed the following unusual behaviour in Mr/Mrs ...?** (multiple answers possible)
   O Walking/runaway tendency (seemingly aimless and aimless wandering)
   O Sleep disturbance
   O Restlessness in the evening
   O Screaming
   O Attempts to get out of bed in inappropriate situations
   O Nodding
   O Verbally aggressive
   O Physically aggressive
   O Visual hallucinations/deceptive images
   O Other: ________________________

5. **Since our last contact, has Mr/Mrs ... required further hospitalisation or contact with a doctor?**
   O Yes O No

*If yes, why?*
O Known medical problem worsened
O New medical problem occurred
O Change of medication
O Other: ___________________________

6. **Has Mr/Mrs ... fallen since our last contact?**
O Yes O No
*If yes, how often?*
_____ times

## 33. Vital signs [T0]

1. **Gender**
O Male O Female

2. **Date of birth** (please enter month and year ONLY!)
___.______ mm.yyyy

3. **Body height**
_____ cm
O not measured

4. **Body weight**

**On admission / when available**
___.___.______ dd.mm.yyyy
_____ kg

O not measured

**On discharge / last value before discharge**
___.___.______ dd.mm.yyyy
_____ kg

O not measured

5. **Blood pressure**

**On admission**
___.___.______ dd.mm.yyyy
_____ / _____ syst/diast. mmHg

O not measured

**On discharge**
___.___.______ dd.mm.yyyy
_____ / _____ syst/diast. mmHg

O not measured

6. **Length of stay in the acute care hospital**

Date of admission to the hospital ___.___.______ dd.mm.yyyy
Date of discharge from the hospital ___.___.______ dd.mm.yyyy

## 34. Blood values [T0]

| *Please specify values in the appropriate unit* | **On admission / when available** | | **On discharge / last value before discharge** | |
|---|---|---|---|---|
| | If all blood values on admission or discharge were taken on the same day, only enter the date here and leave the other date fields in the column blank (enter the lowest and highest values separately). | | | |
| | ___.___.______ | dd.mm.yyyy | ___.___.______ | dd.mm.yyyy |
| **Leukocytes** | ___.___.______ | dd.mm.yyyy | ___.___.______ | dd.mm.yyyy |
| | __________ | Giga/l (10^9/l) | __________ | Giga/l (10^9/l) |
| | __________ | /µl | __________ | /µl |
| | O not measured | | O not measured | |
| **MCV (Mean Cellular Volume)** | ___.___.______ | dd.mm.yyyy | | |
| | __________ | femto-l (fl) | | |
| | O not measured | | | |
| **Haemoglobin (Hb)** | ___.___.______ | dd.mm.yyyy | ___.___.______ | dd.mm.yyyy |
| | __________ | g/dl | __________ | g/dl |
| | O not measured | | O not measured | |
| **Thrombocytes** | ___.___.______ | dd.mm.yyyy | ___.___.______ | dd.mm.yyyy |
| | __________ | Giga/l (10^9/l) | __________ | Giga/l (10^9/l) |
| | __________ | Thousand/µl (10^3/µl) | __________ | Thousand/µl (10^3/µl) |
| | O not measured | | O not measured | |
| **Sodium (Na+)** | ___.___.______ | dd.mm.yyyy | ___.___.______ | dd.mm.yyyy |
| | __________ | mmol/l | __________ | mmol/l |
| | O not measured | | O not measured | |
| **Potassium (K+)** | ___.___.______ | dd.mm.yyyy | ___.___.______ | dd.mm.yyyy |
| | __________ | mmol/l | __________ | mmol/l |
| | O not measured | | O not measured | |
| **Creatinine** | ___.___.______ | dd.mm.yyyy | ___.___.______ | dd.mm.yyyy |
| | __________ | µmol/l | __________ | µmol/l |
| | __________ | mg/dl | __________ | mg/dl |
| | O not measured | | O not measured | |
| **Urea** | ___.___.______ | dd.mm.yyyy | ___.___.______ | dd.mm.yyyy |
| | __________ | mmol/l | __________ | mmol/l |
| | __________ | mg/dl | __________ | mg/dl |
| | O not measured | | O not measured | |
| **Alkaline phosphatase (AP)** | ___.___.______ | dd.mm.yyyy | ___.___.______ | dd.mm.yyyy |
| | __________ | U/l | __________ | U/l |
| | O not measured | | O not measured | |
| **Gamma GT (GGT)** | ___.___.______ | dd.mm.yyyy | ___.___.______ | dd.mm.yyyy |
| | __________ | U/l | __________ | U/l |
| | O not measured | | O not measured | |

TRADE

Project: TRADE observational study
Timepoint: O T0 O T1 O T2 O T3
Centre: _______

Date ___.___.______
Interviewer code number: _____
Participant ID: _____

| | | | | |
|---|---|---|---|---|
| **GOT (AST)** | ___.___.______ dd.mm.yyyy | | ___.___.______ dd.mm.yyyy | |
| | __________ | U/l | __________ | U/l |
| | O not measured | | O not measured | |
| **GPT (ALT)** | ___.___.______ dd.mm.yyyy | | ___.___.______ dd.mm.yyyy | |
| | __________ | U/l | __________ | U/l |
| | O not measured | | O not measured | |
| **HbA1c** | ___.___.______ dd.mm.yyyy | | | |
| | __________ | mmol/l | | |
| | __________ | % | | |
| | O not measured | | | |
| **Total protein** | ___.___.______ dd.mm.yyyy | | | |
| | __________ | mg/dl | | |
| | __________ | g/l or mg/ml | | |
| | O not measured | | | |
| **Albumin** | ___.___.______ dd.mm.yyyy | | | |
| | __________ | g/dl | | |
| | __________ | g/l | | |
| | O not measured | | | |
| **NT-proBNP** | ___.___.______ dd.mm.yyyy | | ___.___.______ dd.mm.yyyy | |
| | __________ | pg/ml | __________ | pg/ml |
| | __________ | ng/l | __________ | ng/l |
| | O not measured | | O not measured | |
| **TSH** | ___.___.______ dd.mm.yyyy | | ___.___.______ dd.mm.yyyy | |
| | __________ | mIU/l | __________ | mIU/l |
| | __________ | mU/l | __________ | mU/l |
| | O not measured | | O not measured | |
| **Blood sugar (glucose)** | **On admission / when available** | | **On discharge / last value before discharge** | |
| | ___.___.______ dd.mm.yyyy | | ___.___.______ dd.mm.yyyy | |
| | __________ | mg/dl | __________ | mg/dl |
| | O not measured | | O not measured | |
| | **Lowest value** | | **Highest value** | |
| | ___.___.______ dd.mm.yyyy | | ___.___.______ dd.mm.yyyy | |
| | __________ | mg/dl | __________ | mg/dl |
| | O not measured | | O not measured | |
| **CRP (C-reactive protein)** | **On admission / when available** | | **On discharge / last value before discharge** | |
| | ___.___.______ dd.mm.yyyy | | ___.___.______ dd.mm.yyyy | |
| | __________ | mg/l | __________ | mg/l |
| | __________ | mg/dl | __________ | mg/dl |
| | O not measured | | O not measured | |

| | Lowest value | | Highest value | |
|---|---|---|---|---|
| | ___.___.______ dd.mm.yyyy | | ___.___.______ dd.mm.yyyy | |
| | __________ | mg/l | __________ | mg/l |
| | __________ | mg/dl | __________ | mg/dl |
| | O not measured | | O not measured | |

## 35. List of medications [T0, T1, T2, T3]

***Please list all medications. First list the medications prescribed by the participant's doctor and then the over-the-counter medications.***
**If required, use the list for recording medication (available in the download area of the homepage).**
**For brand names, please pay attention to the exact designation. Please record combination preparations as individual active ingredients.**

| | Name (active ingredient or brand name) | Type of medication | Single dose | Unit | Frequency | |
|---|---|---|---|---|---|---|
| 1 | ________________ | O long-term medication<br>O on-demand medication | _____,____ | O mg<br>O g<br>O ml<br>O µg<br>O IE<br>O Other:<br>_______ | ______times | O Daily<br>O Weekly<br>O Monthly<br>O Quarterly<br>O Half-yearly<br>O Annual<br>O Other:<br>____________ |
| 2 | ... | | | | | |

## 36. Diagnoses [T0]

**For a better assessment of the pre-existing conditions of our participants, please collect the following pre-existing conditions from the patient medical file at timepoint T0.**

| | Yes | No | Don't know |
|---|---|---|---|
| - High blood pressure | O | O | O |
| - Diabetes | O | O | O |
| - without end organ damage | O | O | O |
| - with end organ damage | O | O | O |
| - Myocardial infarction (incl. acute myocardial infarction) | O | O | O |
| - Calcification of the coronary arteries (incl. coronary heart disease (CHD)) | O | O | O |
| - Heart valve diseases | O | O | O |
| - Heart failure | O | O | O |
| - Cardiac arrhythmia | O | O | O |
| - Atrial fibrillation or flutter (incl. absolute arrhythmia, arrhythmia absoluta (AA)) | O | O | O |
| - Pacemaker | O | O | O |
| - Arterial circulatory disorders of the legs (incl. PAD, intermittent claudication) | O | O | O |
| - Other diseases of the arterial vessels | O | O | O |
| - Chronic lung disease | O | O | O |
| - COPD | O | O | O |
| - Asthma | O | O | O |
| - With home oxygen therapy | O | O | O |
| - Sleep apnoea syndrome (incl. obstructive sleep apnoea syndrome (OSAS)) | O | O | O |
| - Gastric duodenal ulcer | O | O | O |
| - Mild liver disease | O | O | O |
| - Moderately severe and severe liver disease | O | O | O |
| - Moderately severe and severe kidney disease | O | O | O |
| - Malignant tumour diseases | O | O | O |
| - Blood cancer (incl. leukaemia, multiple myeloma (MM), Waldenström's disease) | O | O | O |
| - Lymph gland cancer (incl. lymphoma, Hodgkin's disease, non-Hodgkin's lymphoma (NHL)) | O | O | O |
| - Metastasised solid tumour | O | O | O |
| - HIV | O | O | O |
| - AIDS | O | O | O |
| - Rheumatism, soft tissue and autoimmune diseases | O | O | O |
| - Osteoarthritis | O | O | O |
| - Bone fractures | O | O | O |

**Please list all other diagnoses from the doctor's letter at timepoint T0, entering the main diagnosis (even if already mentioned above) as diagnosis 1:**

| | Diagnosis | Comment |
|---|---|---|
| **1.** | | |
| **2.** | | |
| **3.** | | |

**Note: Document the medication, vital signs, blood values and diagnoses forms directly in secuTrial®.**

## E. Questions for INTERVIEWER [T0]

### 37. Interviewer's assessment [T0]

1. **Hearing ability of the participant**
   Please rate on a scale from 0 to 10: 0 = participant hears very well to 10 = participant hears very poorly
   O 0 O 1 O 2 O 3 O 4 O 5 O 6 O 7 O 8 O 9 O 10

2. **Visual acuity of the participant**
   Please rate on a scale from 0 to 10: 0 = participant has very good vision to 10 = participant has very poor vision
   O 0 O 1 O 2 O 3 O 4 O 5 O 6 O 7 O 8 O 9 O 10

3. **Do you currently have the impression that the participant has cognitive limitations?**
   O Yes O No

   **If yes, please describe briefly**

   ______________________________________________________________

   ______________________________________________________________

   ______________________________________________________________

## F. Drop-out form [in case of drop-out]

**Date of notification**
___.___.______ dd.mm.yyyy

**Time of notification**
____:____ hh:mm

**Interviewer**
O 01 O 02 O 03 O 04 O 05 O 06 O 07 O 08 O 09 O 10 O Other: _____________

**Centre**
O Ulm - Gastroenterology
O Ulm - Trauma surgery
O Heidelberg - Geriatrics
O Heidelberg - Cardiology, Angiology and Pneumology
O Tuebingen - Gastroenterology
O Tuebingen - Cardiology
O Tuebingen - Geriatrics
O Other centre/department: ____________________________________

### *Drop-out - Reason*

**1. Postponement of discharge**
Was the discharge postponed by more than 4 days after the start of the T0 survey?
O Yes O No
***If yes, please state the reason*** __________________________

**2. Postponement of follow-up visits T1, T2 and/or T3**
Was a follow-up visit T1, T2 and/or T3 postponed too far from the time frame of the study plan?
O Yes O No
***If yes, please state the reason*** __________________________

**3. Participant deceased**
Is the participant deceased?
O Yes O No
***If yes, please state date and cause of death, if known***
___.___.______ ______________________________________________

**4. Participant or authorised representative cancels study**
Was the study cancelled?
O Yes O No
***If yes, drop-out due to ...?***
O Participant O Authorised representative
***Please state the reason*** __________________________

**5. Other reasons for dropping out of the study**
Is the participant withdrawing from the study for other reasons?
O Yes O No
***If yes, please state the reason*** __________________________

**6. In exceptional cases, to be avoided if possible**
Does the participant or authorised representative cancel the study and request the deletion of the data?
O Yes O No
***If yes, please state the reason*** __________________________

**7. Which centre doctor has been informed about the study discontinuation (name)?**
__________________________

## G. Adverse events [if required]

### Adverse event - No. ___

**Timing**
O T0 O T1 O T2 O T3

**Interviewer**
O 01 O 02 O 03 O 04 O 05 O 06 O 07 O 08 O 09 O 10 O Other: _______________

**Centre**
O Ulm - Gastroenterology
O Ulm - Trauma surgery
O Heidelberg - Geriatrics
O Heidelberg - Cardiology, Angiology and Pneumology
O Tuebingen - Gastroenterology
O Tuebingen - Cardiology
O Tuebingen - Geriatrics
O Other centre/department: ________________________________________

**Please describe the event(s) that occurred during the interview:**

_______________________________________________________________
_______________________________________________________________
_______________________________________________________________
_______________________________________________________________
_______________________________________________________________
_______________________________________________________________
_______________________________________________________________
_______________________________________________________________
_______________________________________________________________

## Signature

______________________________ ______________________________________

Place, date Signature

**References:**


1. Lubben JE, Gironda MW. Centrality of Social Ties to the Health and Well-Being of Older Adults. In: B. Berkman & L. K. Harooytan, editor. Social Work and Health Care in an Ageing World. New York: Springer; 2003. p. 319–50.
2. Lubben J, Blozik E, Gillmann G, Iliffe S, Von Renteln Kruse W, Beck JC, et al. Performance of an Abbreviated Version of the Lubben Social Network Scale Among Three European Community-Dwelling Older Adult Populations. The Gerontologist. 2006 Aug;46(4):503–13.
3. Lubben Social Network Scale [Internet]. [cited 2025 Mar 4]. Available from: https://www.brandeis.edu/roybal/docs/LSNS_website_PDF.pdf
4. Nasreddine ZS, Phillips NA, Bédirian V, Charbonneau S, Whitehead V, Collin I, et al. The Montreal Cognitive Assessment, MoCA: a brief screening tool for mild cognitive impairment. J Am Geriatr Soc. 2005 Apr;53(4):695–9.
5. MoCA Cognition [Internet]. [cited 2025 Mar 4]. Available from: https://mocacognition.com/paper/
6. Pendlebury ST, Welch SJV, Cuthbertson FC, Mariz J, Mehta Z, Rothwell PM. Telephone assessment of cognition after transient ischemic attack and stroke: modified telephone interview of cognitive status and telephone Montreal Cognitive Assessment versus face-to-face Montreal Cognitive Assessment and neuropsychological battery. Stroke. 2013 Jan;44(1):227–9.
7. Katz MJ, Wang C, Nester CO, Derby CA, Zimmerman ME, Lipton RB, et al. T-MoCA: A valid phone screen for cognitive impairment in diverse community samples. Alzheimers Dement Diagn Assess Dis Monit. 2021 Jan;13(1):e12144.
8. Inouye SK. Clarifying Confusion: The Confusion Assessment Method: A New Method for Detection of Delirium. Ann Intern Med. 1990 Dec 15;113(12):941.
9. Thomas C, Kreisel SH, Oster P, Driessen M, Arolt V, Inouye SK. Diagnosing delirium in older hospitalized adults with dementia: adapting the confusion assessment method to international classification of diseases, tenth revision, diagnostic criteria. J Am Geriatr Soc. 2012 Aug;60(8):1471–7.
10. Sánchez A, Thomas C, Deeken F, Wagner S, Klöppel S, Kentischer F, et al. Patient safety, cost-effectiveness, and quality of life: reduction of delirium risk and postoperative cognitive dysfunction after elective procedures in older adults-study protocol for a stepped-wedge cluster randomized trial (PAWEL Study). Trials. 2019 Jan 21;20(1):71.
11. Deeken F, Sánchez A, Rapp MA, Denkinger M, Brefka S, Spank J, et al. Outcomes of a Delirium Prevention Program in Older Persons After Elective Surgery: A Stepped-Wedge Cluster Randomized Clinical Trial. JAMA Surg. 2022 Feb 1;157(2):e216370.
12. Collen FM, Wade DT, Robb GF, Bradshaw CM. The Rivermead Mobility Index: a further development of the Rivermead Motor Assessment. Int Disabil Stud. 1991;13(2):50–4.
13. Physiopedia: Rivermead Mobility Index [Internet]. [cited 2025 Mar 4]. Available from: https://www.physio-pedia.com/Rivermead_Mobility_Index
14. Kroenke K, Spitzer RL, Williams JBW, Löwe B. An ultra-brief screening scale for anxiety and depression: the PHQ-4. Psychosomatics. 2009;50(6):613–21.
15. The Four-Item Patient Health Questionnaire for Anxiety and Depression (PHQ-4) [Internet]. [cited 2025 Mar 4]. Available from: https://www.recoveryanswers.org/assets/phq-4.pdf
16. Kaspar R, Gabrian M, Brothers A, Wahl HW, Diehl M. Measuring Awareness of Age-Related Change: Development of a 10-Item Short Form for Use in Large-Scale Surveys. The Gerontologist. 2019 May 17;59(3):e130–40.
17. Beierlein C, Kemper CJ, Kovaleva A, Rammstedt B. Short Scale for Measuring General Self-efficacy Beliefs (ASKU). methods. 2017 Feb 6;data:28 Pages.
18. Wilson MMG, Thomas DR, Rubenstein LZ, Chibnall JT, Anderson S, Baxi A, et al. Appetite assessment: simple appetite questionnaire predicts weight loss in community-dwelling adults and nursing home residents. Am J Clin Nutr. 2005 Nov;82(5):1074–81.

19. Buysse DJ, Reynolds CF, Monk TH, Berman SR, Kupfer DJ. The Pittsburgh Sleep Quality Index: a new instrument for psychiatric practice and research. Psychiatry Res. 1989 May;28(2):193–213.

20. Lawton MP, Brody EM. Assessment of older people: self-maintaining and instrumental activities of daily living. The Gerontologist. 1969;9(3):179–86.

21. CGA Toolkit plus: Lawton IADL Scale [Internet]. [cited 2025 Mar 4]. Available from: https://www.cgakit.com/f-1-lawton-scale

22. Steis MR, Evans L, Hirschman KB, Hanlon A, Fick DM, Flanagan N, et al. Screening for delirium using family caregivers: convergent validity of the Family Confusion Assessment Method and interviewer-rated Confusion Assessment Method. J Am Geriatr Soc. 2012 Nov;60(11):2121–6.

23. Inouye S, Puelle M, Saczynski J, Steis M. The Family Confusion Assessment Method (FAM-CAM): Instrument and Training Manual. [Internet]. Hospital Elder Life Program, Boston; 2012 [cited 2025 Mar 4]. Available from: https://americandeliriumsociety.org/wp-content/uploads/2021/08/FAMCAM_TrainingManual.pdf

24. Jorm AF, Jacomb PA. The Informant Questionnaire on Cognitive Decline in the Elderly (IQCODE): socio-demographic correlates, reliability, validity and some norms. Psychol Med. 1989 Nov;19(4):1015–22.

25. Ehrensperger MM, Berres M, Taylor KI, Monsch AU. Screening properties of the German IQCODE with a two-year time frame in MCI and early Alzheimer's disease. Int Psychogeriatr. 2010 Feb;22(1):91–100.

26. Australian National University, National Centre for Epidemiology and Population Health: Informant questionnaire on cognitive decline in the elderly [Internet]. [cited 2025 Mar 4]. Available from: https://nceph.anu.edu.au/research/tools-resources/informant-questionnaire-cognitive-decline-elderly

27. Gaudreau JD, Gagnon P, Harel F, Tremblay A, Roy MA. Fast, Systematic, and Continuous Delirium Assessment in Hospitalized Patients: The Nursing Delirium Screening Scale. J Pain Symptom Manage. 2005 Apr;29(4):368–75.

28. Brich J, Baten V, Wußmann J, Heupel-Reuter M, Perlov E, Klöppel S, et al. Detecting delirium in elderly medical emergency patients: validation and subsequent modification of the German Nursing Delirium Screening Scale. Intern Emerg Med. 2019 Aug;14(5):767–76.

29. Mahoney FI, Barthel DW. Functional evaluation: The Barthel Index. Md State Med J. 1965 Feb;14:61–5.

30. The Barthel Index [Internet]. [cited 2025 Mar 4]. Available from: https://www.sralab.org/sites/default/files/2017-07/barthel.pdf
